# Burden of pulmonary arterial hypertension in children globally, regionally, and nationally (1990–2021): results from the global burden of disease study

**DOI:** 10.3389/fped.2025.1527281

**Published:** 2025-06-30

**Authors:** Lili Deng, Jingxuan Xiong, Jiaoli Xu, Qinhong Li, Zugen Cheng

**Affiliations:** ^1^ Department of Cardiology, Kunming Children's Hospital, Kunming City, Yunnan, China; ^2^ Department of Respiratory Medicine, Kunming Children's Hospital, Kunming City, Yunnan, China; ^3^ Department of Cardiology, The First Affiliated Hospital of Kunming Medical University, Kunming City, Yunnan, China

**Keywords:** pulmonary arterial hypertension (PAH), public health, GBD (2021) database, prevalence, mortaltity, DALYs—disability-adjusted life years

## Abstract

**Introduction:**

Pediatric pulmonary arterial hypertension (PAH) is a rare and severe disorder characterized by obstructive vascular changes that can lead to right heart failure. The clinical presentation and underlying causes of pediatric PAH differ significantly from those in adults, often involving congenital heart disease and developmental lung disorders, such as bronchopulmonary dysplasia (BPD). Despite advances in treatment, pediatric PAH remains underrecognized globally.

**Methods:**

This study analyzed global, regional, and national trends in pediatric PAH from 1990 to 2021 using data from the Global Burden of Disease (GBD) database.

**Results:**

The findings indicate a stable prevalence rate globally, with a slight increase in the absolute number of cases. Significantly, reductions were observed in both mortality and disability-adjusted life years (DALYs) associated with pediatric PAH, with mortality decreasing by 57.66% and DALYs by 63.59% over the study period, indicating progress in mitigating the disease burden. Substantial regional disparities were identified, with low-income regions, particularly Low Socio-Demographic Index (SDI) areas, experiencing the highest mortality and DALY rates. In contrast, high-middle SDI regions showed the greatest reductions in disease burden. The highest prevalence and burden were observed in South Asia, the Caribbean, and parts of Sub-Saharan Africa, with China, India, and Haiti bearing the greatest national burdens.

**Discussion:**

These findings highlight the necessity for targeted health interventions, especially in low-resource settings, to improve early diagnosis, intervention, and treatment.

## Introduction

Pulmonary arterial hypertension (PAH) is a rare and severe disease characterized by progressive obstructive vascular lesions in the distal pulmonary arterial circulation, ultimately leading to right heart failure and death (1, 2). Recent studies have reported a mortality rate of 0.27 per 100,000 population in 2021 (3). The pathophysiological mechanisms of PAH are complex, often involving endothelial dysfunction, vascular remodeling, and inflammation (4). The presentation of this disease in children differs significantly from that in adults, with more diverse etiologies often associated with various underlying conditions (5). In contrast, PAH in adults is more commonly secondary to left heart disease or chronic lung disease, highlighting the distinct challenges in the management of pediatric PAH (6, 7). Pediatric PAH can occur at any age and is frequently associated with developmental or genetic disorders, with an etiological distribution distinct from that seen in adults (8, 9).

The major causes of pediatric PAH include idiopathic pulmonary arterial hypertension (IPAH), PAH associated with congenital heart disease (PAH-CHD) (10), and PAH related to developmental lung disease (5). Among children, PAH-CHD is one of the most common forms, particularly prevalent in those with congenital heart defects who have not undergone timely surgical intervention (11). Developmental lung diseases, such as bronchopulmonary dysplasia (BPD) and prematurity, also contribute significantly to the etiology of pediatric PAH (12).

Despite significant advances in the treatment of PAH in recent years, the prognosis for pediatric PAH remains poor, and its global burden has not been fully recognized (3). Utilizing data from the Global Burden of Disease (GBD) database, evaluating the global, regional, and national burden of pediatric PAH is crucial for understanding its epidemiology, formulating effective public health strategies, and improving patient care and management. This study aims to systematically assess the global, regional, and national burden of pediatric PAH from 1990 to 2021 using the GBD database, analyze its epidemiological trends, and provide a scientific basis for future interventions.

Globally, there are significant disparities in the disease burden between different regions, influenced not only by genetic factors but also by differences in access to healthcare resources and diagnostic capabilities (13). In many low- and middle-income countries, early identification and intervention for pediatric PAH remain challenging due to a lack of adequate diagnostic and treatment facilities (14). Additionally, the nonspecific symptoms of pediatric PAH frequently result in misdiagnosis or diagnostic delays, with most cases identified only during advanced disease stages (15, 16). Therefore, increasing awareness of pediatric PAH and enhancing early screening and diagnosis are critical for improving outcomes in affected children.

## Methods

### Overview and data collection

This study was conducted with approval from Kunming Children's Hospital. The Ethics Committee of Kunming Children's Hospital granted a waiver of informed consent, as the study involved only secondary data analysis of anonymized information without any personally identifiable data. Data on pediatric PAH in children aged 0–14 years were obtained from the Global Health Data Exchange (GHDx) query tool, developed by the GBD collaborators, using standardized disease definitions and prevalence estimates. To access similar data, visit the GHDx website at: https://ghdx.healthdata.org/.

The 2021 GBD study assessed prevalence, mortality, and disability-adjusted life years (DALYs), along with corresponding rates and uncertainty intervals, for 369 diseases and injuries across 204 countries and territories from 1990 to 2021 (17). The data on the prevalence and number of PAH cases, PAH-related mortality, and the burden of pediatric PAH-related comorbidities, as well as corresponding rates at the global, regional, and national levels, were collected.

The GBD database did not provide information on racial or ethnic data for participants, as race and ethnicity were not assigned during data collection. Linear regression analysis was performed to estimate the annual percentage change (EAPC) (18). This study adhered to the Strengthening the Reporting of Observational Studies in Epidemiology (STROBE) guidelines (19).

### Sociodemographic Index

The Socio-demographic Index (SDI) is a key metric that integrates fertility rate, educational attainment, and per capita income to gauge the level of development of a country or region, ranging from 0 to 1, with higher values indicating greater socio-economic advancement (20). Previous studies have demonstrated a significant association between SDI and both disease prevalence and mortality (21). In this study, countries and geographic regions are categorized into five SDI levels (low, low-middle, middle, high-middle, and high) to explore the relationship between socioeconomic development and the burden of pediatric PAH.

### Definitions

Historically, PAH in children has been defined using the same criteria as in adults: a mean pulmonary arterial pressure (mPAP) of ≥25 mmHg (22, 23). In normal fetal circulation, pulmonary arterial pressure is comparable to systemic pressure, but it declines rapidly after birth, reaching levels similar to those of adults by the age of 2–3 months. Given the variability in pulmonary hemodynamics during the postnatal transition, pediatric PAH is defined as an mPAP of ≥25 mmHg after 3 months of age (5). Estimation of prevalence, incidence, and mortality followed the standard GBD workflow but incorporated paediatric-specific assumptions to address data sparsity. Briefly, DisMod-MR 2.1 Bayesian meta-regression pooled all available hospital discharge, administrative claims, and registry data (21 countries, 11 paediatric cohorts) across ages and imposed age-specific random effects. A zero-prevalence prior was applied from birth to 3 months, with a spline-based ramp from 3 months to 15 years to reflect the biological onset of disease. Covariates capturing national CHD prevalence, neonatal survival, HIV prevalence, and the SDI were introduced to borrow strength where pediatric data were scarce. Cause-specific mortality was modelled with CODEm, constrained to the all-cause mortality envelope, and uncertainty was propagated through 1,000 posterior draws. All estimates are presented with 95% uncertainty intervals (UIs) (24).

### Statistical analysis

Prevalence, mortality, DALYs, and their respective ratios are key indicators of the burden of pediatric PAH. Using the GBD methodology, we report prevalence rates per 100,000 population, along with the corresponding 95% UI. To analyze temporal trends in pediatric PAH, we used the EAPC to assess shifts in disease burden over time, with linear modeling employed to derive the 95% confidence interval (CI) for the EAPC. If the upper bound of the EAPC and its 95% CI are negative, this suggests a declining trend in prevalence; conversely, if the lower bound of the EAPC and its 95% CI are positive, this indicates an increasing trend (25).

## Results

### PAH in children: global trends

#### Prevalence

In 2021, the estimated global prevalence of pediatric PAH was 8,973.59 cases (95% UI, 6,312.64–11,961.61), compared to 7,614.65 cases (95% UI, 5,374.55–10,085.40) in 1990. From 1990 to 2021, the number of prevalent cases increased by 17.85% (95% UI, 0.22–3.37). Despite this increase, the prevalence rate showed minimal change, rising slightly from 0.44 (95% UI, 0.31–0.58) per 100,000 in 1990 to 0.45 (95% UI, 0.31–0.59) per 100,000 in 2021. EAPC was −0.01 (95% CI, −0.05–0.03) (Table 1).

**Table 1:** Prevalence of pediatric pulmonary arterial hypertension at global and regional levels from 1990 to 2021.

Location	1990		2021		1990–2021	
	Prevalent Cases	Prevalent Rate	Prevalent Cases	Prevalent Rate	Cases change	EAPC^a^
Global	7,614.65 (5,374.55, 10,085.40)	0.44 (0.31, 0.58)	8,973.59 (6,312.64, 11,961.61)	0.45 (0.31, 0.59)	17.85 (15.94, 19.58)	−0.01 (−0.05, 0.03)
High SDI	1,024.32 (750.81, 1,321.37)	0.55 (0.40, 0.71)	961.34 (703.95, 1,236.32)	0.56 (0.41, 0.72)	−6.15 (−7.37, −4.89)	−0.00 (−0.02, 0.01)
High-middle SDI	1,554.51 (1,131.59, 2,010.16)	0.57 (0.41, 0.73)	1,382.97 (1,002.64, 1,794.11)	0.60 (0.43, 0.78)	−11.04 (−12.71, −9.48)	0.08 (0.04, 0.12)
Middle SDI	2,596.63 (1,815.16, 3,445.67)	0.45 (0.31, 0.60)	2,792.06 (1,961.37, 3,718.50)	0.49 (0.35, 0.66)	7.53 (6.32, 8.86)	0.17 (0.06, 0.28)
Low-middle SDI	1,615.61 (1,109.94, 2,174.20)	0.34 (0.24, 0.46)	2,221.20 (1,538.65, 3,003.34)	0.38 (0.27, 0.52)	37.48 (34.95, 40.10)	0.38 (0.35, 0.40)
Low SDI	815.15 (561.52, 1,101.96)	0.36 (0.25, 0.48)	1,608.63 (1,103.08, 2,180.62)	0.35 (0.24, 0.47)	97.34 (92.80, 102.07)	−0.07 (−0.14, −0.00)
Regions						
Andean Latin America	82.05 (57.74, 109.81)	0.55 (0.39, 0.74)	97.27 (68.34, 131.44)	0.54 (0.38, 0.73)	18.55 (12.82, 25.24)	−0.16 (−0.28, −0.04)
Australasia	25.75 (18.56, 32.88)	0.56 (0.40, 0.72)	31.98 (23.41, 41.68)	0.56 (0.41, 0.73)	24.20 (16.01, 32.65)	−0.07 (−0.11, −0.03)
Caribbean	61.58 (44.52, 80.14)	0.54 (0.39, 0.70)	55.76 (39.60, 74.25)	0.48 (0.34, 0.65)	−9.44 (−13.99, −4.68)	−0.44 (−0.55, −0.33)
Central Asia	149.60 (108.50,193.03)	0.60 (0.43, 0.77)	165.42 (119.16, 215.61)	0.60 (0.43, 0.78)	10.57 (6.31, 14.87)	−0.08 (−0.13, −0.04)
Central Europe	236.02 (176.01, 298.99)	0.80 (0.60, 1.01)	138.01 (101.39, 175.12)	0.78 (0.57, 0.99)	−41.53 (−42.88, −39.83)	−0.15 (−0.23, −0.08)
Central Latin America	370.97 (263.46, 490.77)	0.58 (0.41, 0.76)	441.10 (319.84, 573.04)	0.69 (0.50, 0.90)	18.90 (14.83, 22.80)	0.33 (0.16, 0.49)
Central Sub-Saharan Africa	115.57 (79.42, 154.67)	0.46 (0.31, 0.61)	183.54 (124.55, 251.32)	0.31 (0.21, 0.43)	58.82 (45.76, 73.91)	−1.12 (−1.27, −0.97)
East Asia	1,496.70 (1,049.87, 1,993.79)	0.45 (0.32, 0.60)	1,347.51 (948.34, 1,782.43)	0.50 (0.35, 0.67)	−9.97 (−11.85, −8.10)	0.22 (0.10, 0.33)
Eastern Europe	425.22 (316.94, 540.13)	0.83 (0.62, 1.05)	297.64 (221.34, 378.54)	0.84 (0.62, 1.07)	−30.00 (−32.00, −27.97)	−0.32 (−0.49, −0.14)
Eastern Sub-Saharan Africa	369.16 (252.41, 498.55)	0.41 (0.28, 0.55)	645.58 (442.89, 877.36)	0.36 (0.25, 0.49)	74.88 (69.63, 80.11)	−0.63 (−0.80, −0.46)
High-income Asia Pacific	226.60 (167.56, 289.41)	0.64 (0.48, 0.82)	140.41 (103.79, 179.43)	0.63 (0.46, 0.80)	−38.03 (−39.58, −36.29)	−0.04 (−0.10, 0.03)
High-income North America	236.72 (172.30, 306.20)	0.38 (0.28, 0.50)	250.03 (179.59, 328.28)	0.38 (0.27, 0.50)	5.62 (2.08, 9.26)	−0.04 (−0.09, 0.01)
North Africa and Middle East	575.99 (398.38, 774.50)	0.41 (0.28, 0.55)	742.89 (512.40, 1,002.73)	0.41 (0.28, 0.55)	28.98 (25.63, 32.76)	−0.17 (−0.41, 0.08)
Oceania	10.44 (7.18, 14.04)	0.39 (0.27, 0.52)	17.84 (12.02, 24.31)	0.35 (0.24, 0.48)	70.94 (59.64, 82.46)	0.07 (−0.07, 0.21)
South Asia	1,326.95 (909.97, 1,803.94)	0.31 (0.21, 0.42)	1,714.39 (1,176.68, 2,351.03)	0.34 (0.23, 0.46)	29.20 (25.76, 32.69)	0.35 (0.33, 0.37)
Southeast Asia	653.29 (452.56, 872.42)	0.38 (0.27, 0.51)	705.20 (489.82, 949.57)	0.41 (0.28, 0.55)	7.95 (5.40, 10.29)	0.20 (0.01, 0.39)
Southern Latin America	74.19 (53.35, 96.25)	0.50 (0.36, 0.64)	78.25 (57.10, 101.94)	0.54 (0.39, 0.70)	5.47 (−0.63, 11.62)	0.13 (0.07, 0.19)
Southern Sub-Saharan Africa	95.55 (65.56, 131.05)	0.46 (0.32,0.63)	120.88 (83.93, 163.74)	0.50 (0.35,0.68)	26.50 (22.27, 30.74)	0.01 (−0.09, 0.11)
Tropical Latin America	287.30 (204.89, 379.74)	0.54 (0.38, 0.71)	273.54 (196.36, 360.57)	0.54 (0.39, 0.72)	−4.79 (−7.03, −2.50)	−0.05 (−0.21, 0.10)
Western Europe	452.69 (329.77, 590.38)	0.64 (0.46, 0.83)	485.45 (359.78, 622.94)	0.71 (0.53, 0.91)	7.24 (3.40, 11.41)	0.31 (0.27, 0.34)
Western Sub-Saharan Africa	342.32 (235.52, 460.72)	0.39 (0.27, 0.52)	1,040.91 (713.43, 1,395.59)	0.48 (0.33, 0.65)	204.08 (188.66, 220.35)	1.02 (0.84, 1.20)

EAPC, estimated annual percentage change; SDI, Sociodemographic Index; UI, uncertainty interval.

^a^ EAPC is expressed as 95% CIs.

#### Mortality

In 2021, an estimated 1,714.56 deaths (95% UI, 1,357.56–2,102.27) were attributed to pediatric PAH globally, marking a substantial decline from 4,049.32 deaths (95% UI, 2,365.91–5,629.40) reported in 1990. From 1990 to 2021, PAH-related mortality in children decreased by 57.66% (95% UI, −68.78 to −38.89). Similarly, the PAH-related mortality rate declined from 0.23 (95% UI, 0.14–0.32) per 100,000 in 1990 to 0.09 (95% UI, 0.07–0.10) per 100,000 in 2021, with an EAPC of −2.60 (95% CI, −2.83 to −2.39) (Supplementary Table S1).

#### DALYs

The burden of pediatric PAH, measured by DALYs, showed a marked reduction. In 2021, the number of DALYs attributable to pediatric PAH was 151,098.12 (95% UI, 119,751.94–185,128.35), significantly lower than the 358,741.23 (95% UI, 209,032.75–498,948.49) reported in 1990, representing a 63.59% reduction (95% UI, −73.18 to −47.38). The DALY rate also decreased from 20.63 (95% UI, 12.02–28.69) per 100,000 in 1990 to 7.51 (95% UI, 5.95–9.20) per 100,000 in 2021, with an EAPC of −2.62 (95% CI, −2.84 to −2.40) (Supplementary Table S2).

### PAH in children: SDI regional trends

#### Prevalence

**Figure 1:**
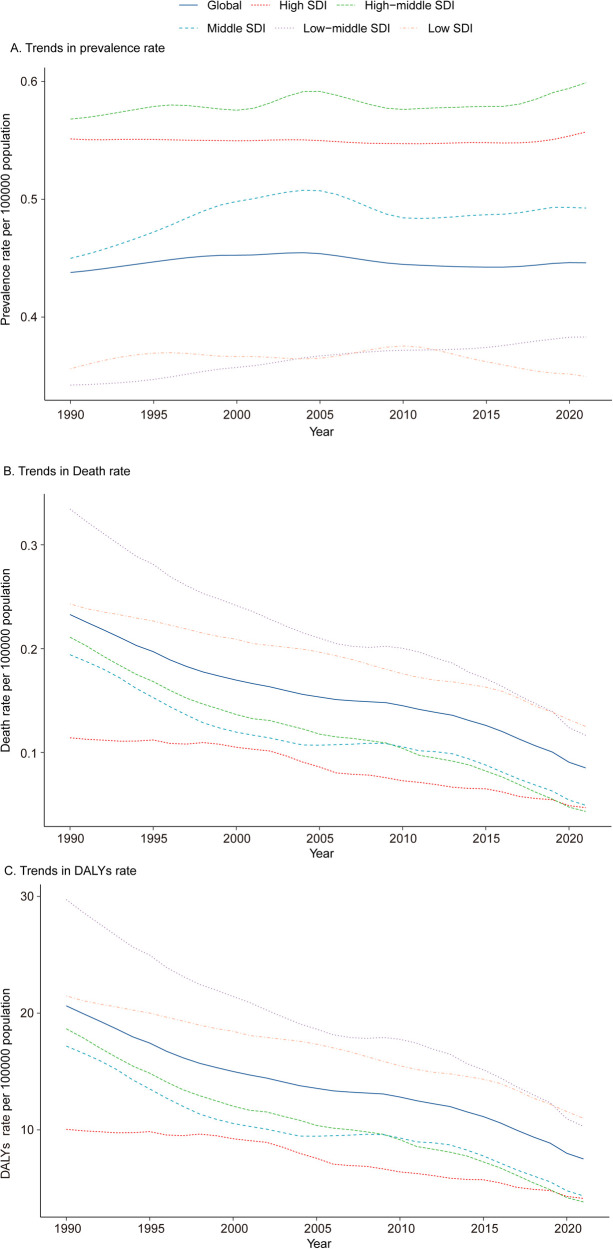
Epidemiologic trends in prevalence, mortality, and disability-adjusted life years (DALYs) of pediatric pulmonary arterial hypertension across five sociodemographic Index (SDI) regions from 1990 to 2021. **(A)** Trends in Prevalence Rate. **(B)** Trends in Mortality. **(C)** Trends in DALY Rate. DALYs, disability-adjusted life-years.

In 1990, the Middle SDI region reported the highest number of pediatric PAH cases, totaling 2,596.63 (95% UI, 1,815.16–3,445.67). This trend persisted in 2021, with the number of cases rising to 2,792.06 (95% UI, 1,961.37–3,718.50). Despite the increase in absolute case numbers, the prevalence rate in the Middle SDI region showed minimal change, shifting from 0.45 (95% UI, 0.31–0.60) per 100,000 in 1990 to 0.49 (95% UI, 0.35–0.66) per 100,000 in 2021. The Low-middle SDI region experienced the most pronounced increase in prevalence rate (EAPC, 0.38; 95% CI, 0.35–0.40), whereas the Low SDI region exhibited the largest decrease (EAPC, −0.07; 95% CI, −0.14 to −0.00) (Table 1 and Figure 1A).

#### Mortality

**Figure 2:**
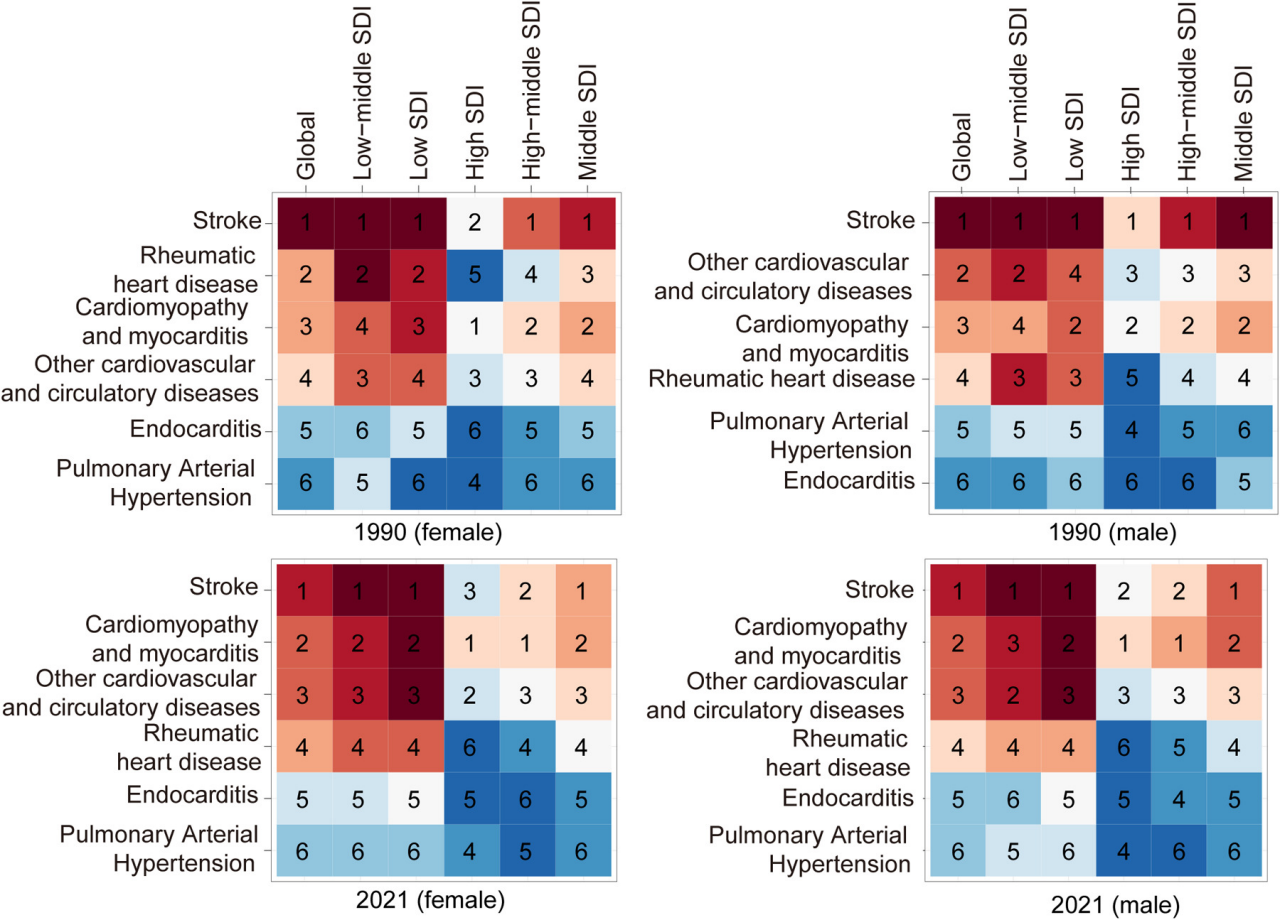
Changes in causes of cardiovascular mortality among children globally and across different sociodemographic Index (SDI) regions: a comparison Between 1990 and 2021. Heatmap representing a 6 × 6 data matrix with inverse intensity scaling (1 = highest value, 6 = lowest value).

Among the five SDI regions, only the Low SDI region exhibited an increase in mortality, rising by 3.48%. In 2021, the Low SDI region also had the highest number of PAH-related deaths, totaling 575.91 (95% UI, 420.09–796.75). In contrast, the High-middle SDI region demonstrated the largest decline in mortality, decreasing by 82.69%, and had the fewest PAH-related deaths in 2021, with 81.01 (95% UI, 73.34–87.79). The pediatric PAH-related mortality rate in 2021 was highest in the Low SDI region, at 0.13 (95% UI, 0.09–0.17) per 100,000, while the High-middle SDI region reported the lowest rate, at 0.04 (95% UI, 0.04–0.06) per 100,000. The most significant decline in mortality rate, as indicated by the lowest EAPC, was observed in the High-middle SDI region (−4.17; 95% CI, −4.54 to −3.80) (Supplementary Table S1 and Figure 1B). A heatmap further illustrates the shifts in the ranking of cardiovascular disease causes of death among children under 14 years old between 1990 and 2021 (per 100,000 population) (Figure 2).

#### DALYs

In 2021, the Low SDI region recorded the highest number of DALYs attributable to PAH, with 50,592.43 (95% UI, 36,915.79–69,903.99), representing a 48.83% reduction from 1990. The DALY rate for pediatric PAH in 2021 was also highest in the Low SDI region, at 10.99 (95% UI, 8.02–15.19) per 100,000, while the High-middle SDI region had the lowest DALY rate, at 3.83 (95% UI, 3.15–4.86) per 100,000. Notably, the High-middle SDI region exhibited the most significant reduction in DALY rate, as indicated by the highest EAPC (−1.89; 95% CI, −2.03 to −1.75) (Supplementary Table S2 and Figure 1C).

### PAH in children: geographic regional trends

#### Prevalence

**Figure 3:**
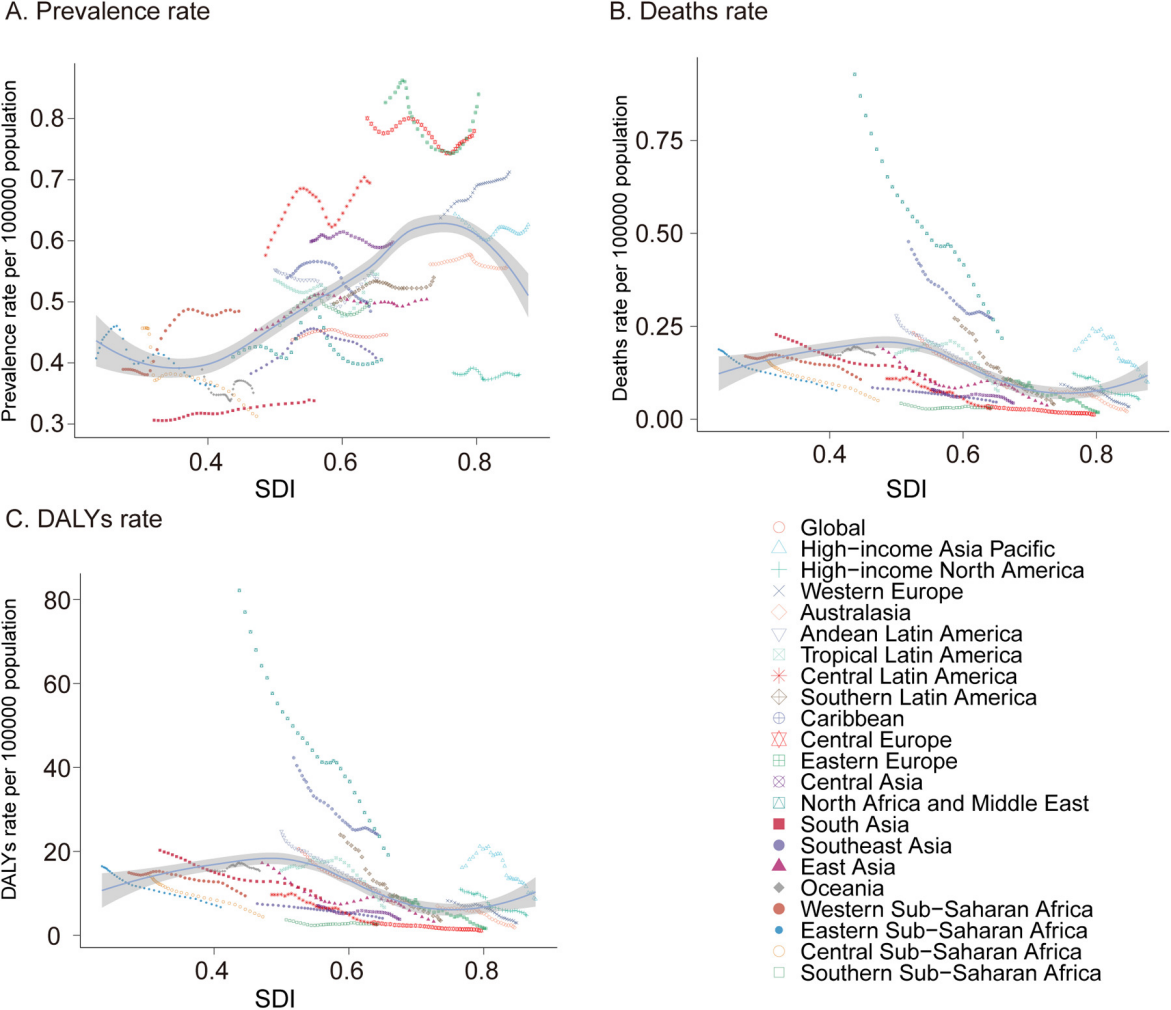
Prevalence, mortality, and disability-adjusted life years (DALYs) of pediatric pulmonary arterial hypertension from 1990 to 2021. **(A)** Prevalence rate. **(B)** Mortality. **(C)** DALYs rate. DALYs, disability-adjusted life-years.

In 2021, South Asia reported the highest number of pediatric PAH cases among the 21 global regions, with an estimated 1,714.39 cases (95% UI, 1,176.68–2,351.03), while Oceania had the lowest number of cases, at 17.84 (95% UI, 12.02–24.31). The highest prevalence rate was observed in South Asia, whereas Oceania had the lowest. From 1990 to 2021, the most pronounced increases in pediatric PAH prevalence rates were seen in Western Sub-Saharan Africa (EAPC, 1.02; 95% CI, 0.84–1.20), South Asia (EAPC, 0.35; 95% CI, 0.16–0.49), and Central Latin America (EAPC, 0.33; 95% CI, −0.09–0.11). By contrast, Southern Sub-Saharan Africa exhibited the smallest increase in prevalence rate (EAPC, 0.01; 95% CI, −0.09–0.11) (Table 1 and Figure 3A).

#### Mortality

In 2021, South Asia also recorded the highest number of pediatric PAH-related deaths, with 508.77 cases (95% UI, 297.82–784.85), whereas Australasia had the lowest number, with 1.203 cases (95% UI, 0.98–1.48). The highest mortality rate was observed in the Caribbean (0.27; 95% UI, 0.10–0.49), while Central Europe had the lowest mortality rate (0.01; 95% UI, 0.01–0.02). Notably, Oceania was the only region that experienced an increase in the pediatric PAH-related mortality rate (EAPC, 0.16; 95% CI, −0.01–0.33), whereas Southern Sub-Saharan Africa showed the smallest decline (EAPC, −0.29; 95% CI, −0.67–0.09), and Southern Latin America exhibited the most substantial decrease (EAPC, −5.62; 95% CI, −5.90 to −5.34) (Supplementary Table S1 and Figure 3B).

#### DALYs

In terms of DALYs attributable to pediatric PAH in 2021, South Asia had the highest burden, with 45,121.64 DALYs (95% UI, 26,371.51–69,880.78), while Australasia had the lowest, with 107.39 DALYs (95% UI, 87.40–132.29). The Caribbean reported the highest DALY rate (24.09; 95% UI, 9.28–43.47), whereas Central Europe had the lowest rate (1.13; 95% UI, 0.99–1.47). Between 1990 and 2021, Southern Sub-Saharan Africa had the smallest decline in DALY rate (EAPC, −0.3; 95% CI, −0.68–0.08), while East Asia experienced the most marked reduction (EAPC, −5.65; 95% CI, −5.93 to −5.37) (Supplementary Table S2 and Figure 3C). These findings highlight significant regional disparities in the prevalence, mortality, and overall disease burden of pediatric PAH, with the greatest burden observed in South Asia and the Caribbean and the least in Australasia and Central Europe. The variations in trends across regions underscore the need for targeted interventions to address the specific challenges faced by each region.

### PAH in children: national trends

#### Prevalence

**Figure 4:**
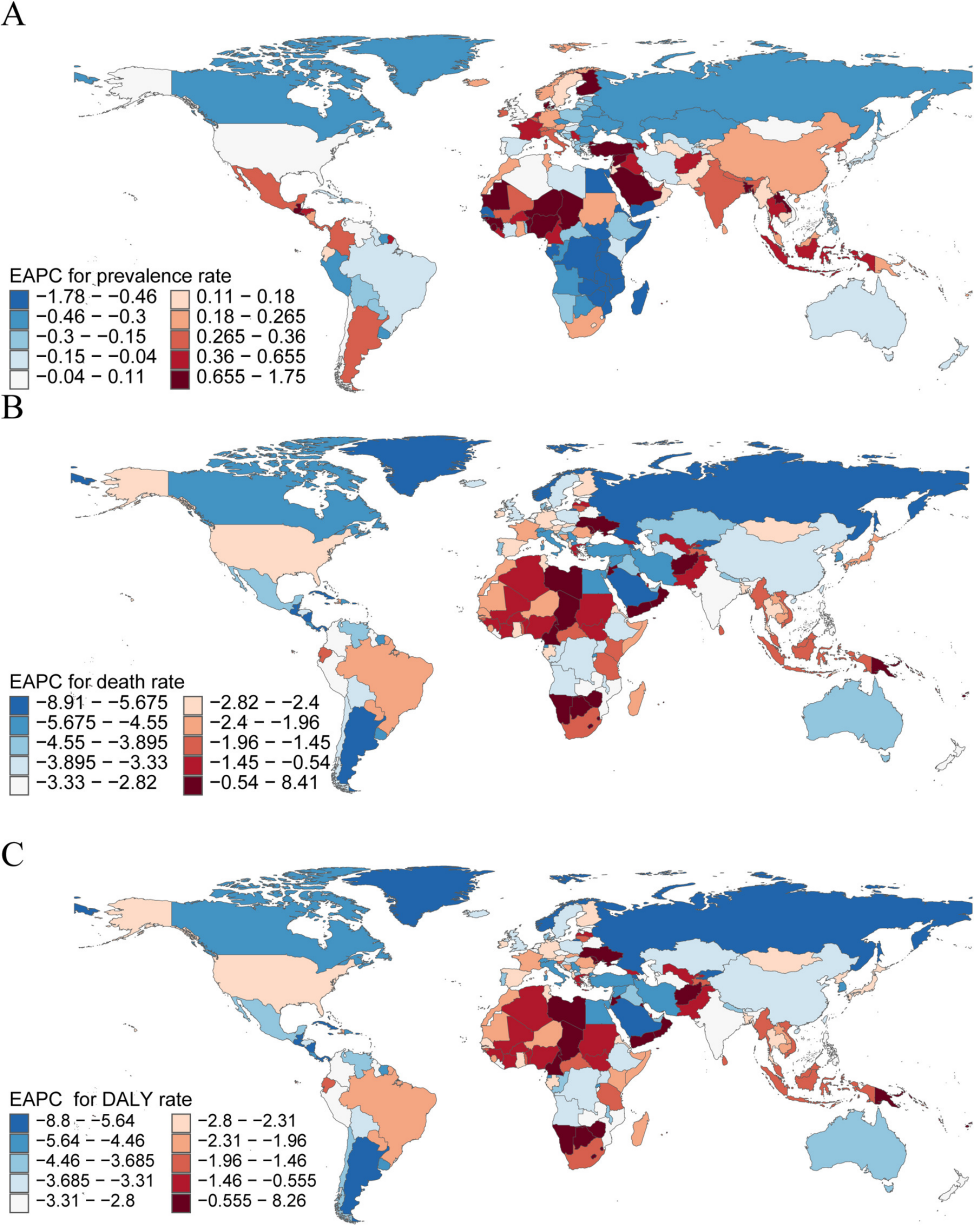
The national burden of pediatric pulmonary arterial hypertension in 204 countries and territories. **(A)** EAPC for Prevalence rate. **(B)** EAPC for Mortality. **(C)** EAPC for DALYs rate. disability-adjusted life-years; EAPC, estimated annual percentage change.

In 2021, China reported the highest number of pediatric PAH cases among 204 countries, with an estimated 1,308 cases (95% UI, 920–1,730), reflecting a decrease of 8.95% since 1990 (95% UI, −10.81 to −7.04). In contrast, Niue and Tokelau reported no pediatric PAH cases. Switzerland had the highest prevalence rate (1.45 per 100,000; 95% UI, 1.03–1.88), whereas Angola had the lowest (0.26 per 100,000; 95% UI, 0.18–0.37). Nigeria experienced the largest increase in prevalence rate (EAPC, 1.75; 95% CI, 1.51–1.98), while Chile had the smallest increase (EAPC, 0.01; 95% CI, −0.14 to 0.15). Egypt demonstrated the most substantial decrease in prevalence rate (EAPC, −1.78; 95% CI, −2.10 to −1.47) (Supplementary Table S3, Figure 4A, and Supplementary Figure S1A). In 2021, Switzerland (SDI, 0.93) had the highest prevalence rate, while Angola (SDI, 0.45) had the lowest (Supplementary Table S3A).

#### Mortality

In 2021, India recorded the highest number of pediatric PAH-related deaths, with 295 cases (95% UI, 178–472), representing a decrease of 57.86% since 1990 (95% UI, −70.00 to −32.43). San Marino reported the fewest deaths, with only 0.0002 cases (95% UI, 0.0001–0.0005). Haiti had the highest mortality rate (0.61 per 100,000; 95% UI, 0.20–1.17). The Republic of Mauritius experienced the largest increase in mortality rate (EAPC, 8.41; 95% CI, 5.76–11.13), while Puerto Rico (EAPC, −8.21; 95% CI, −9.36 to −7.05), Belize (EAPC, −8.91; 95% CI, −9.51 to −8.30), and Norway (EAPC, −8.38; 95% CI, −9.33 to −7.42) had the most significant declines (Supplementary Table S4, Figure 4B, and Supplementary Figure S1B). In 2021, Haiti (SDI, 0.45) had the highest mortality rate, while Slovenia (SDI, 0.84) had the lowest (Supplementary Figure S3B).

#### DALYs

In 2021, India had the highest number of DALYs attributable to pediatric PAH, with 26,219 DALYs (95% UI, 15,801–42,145), reflecting a decrease of 57.93% since 1990 (95% UI, −70.02 to −32.47). San Marino had the fewest DALYs, with 0.03 (95% UI, 0.01–0.05). Haiti reported the highest DALY rate (54.18 per 100,000; 95% UI, 17.26–103.28). Mauritius experienced the largest increase in DALY rate (EAPC, 8.26; 95% CI, 1.98–3.82), whereas Puerto Rico (EAPC, −8.80; 95% CI, −9.40 to −8.20) and Belize (EAPC, −8.42; 95% CI, −8.95 to −7.89) showed the most significant declines (Supplementary Table S5, Figure S3C, and Figure S1C). In 2021, Haiti (SDI, 0.45) had the highest DALY rate, while Slovenia (SDI, 0.84) had the lowest (Supplementary Figure S3C). These findings underscore substantial disparities in pediatric PAH prevalence, mortality, and disease burden across countries, with the highest burdens observed in China, India, and Haiti. The notable variations across countries and regions highlight the importance of targeted health interventions tailored to the specific challenges of each region.

## Discussion

Over the past three decades, the prevalence of PAH among children aged 0–14 has steadily increased worldwide, reflecting an urgent public health concern given the associated medical and social costs. This study leverages data from the GBD database from 1990 to 2021 to examine the prevalence, mortality, and DALYs attributable to pediatric PAH across different regions and countries. The findings provide critical insights into the burden of pediatric PAH in countries with varying income levels, highlighting a consistent rise in prevalence in specific areas over the past thirty years. A comprehensive global assessment of the epidemiological trends of pediatric PAH offers valuable guidance for policymakers and clinicians to develop more effective prevention and management strategies, ultimately enhancing health outcomes for affected children.

Global analyses of pediatric PAH over the past three decades reveal that the overall prevalence of the disease has remained essentially unchanged, consistent with improved survival and earlier detection, even as mortality and disease burden have markedly decreased. Prevalence rates in 2021 were similar to those in 1990, indicating that the prevalence of pediatric PAH and the pool of affected children have not been drastically reduced over time. The number of pediatric PAH cases grew (reflecting population growth and improved detection), yet mortality and DALYs dropped by over 50%. This divergence suggests that while children continue to develop PAH at comparable rates, fewer are dying from it, and those affected are living longer, healthier lives. The data underscore a major success in pediatric PAH management: improved survival and reduced health losses, despite no substantial change in how often the condition occurs. These trends largely reflect advances in medical care, rather than the primary prevention of PAH. The stable prevalence implies that the root causes—congenital heart disease, heritable mutations, neonatal conditions, and other pediatric risk factors—remain prevalent worldwide. However, the steep reduction in mortality and DALYs signals that modern interventions are altering the disease's trajectory. Children with PAH today experience a more chronic course and longer survival than in the 1990s, when the diagnosis was often swiftly fatal. Notably, in the pre-prostacyclin era (before ∼1995), untreated idiopathic PAH in children carried a dismal median survival of only about 10 months, compared to ∼2.8 years in adults (26). By contrast, in the current era of targeted treatments, pediatric registries report 5-year survival rates on the order of 70%–85%—a remarkable improvement attributable to better therapies and multidisciplinary care (27). In essence, PAH has evolved from an acutely lethal pediatric disease to a more manageable chronic condition for many patients, thanks to progress in treatment and supportive care.

Regional differences in PAH etiologies significantly contribute to these observed disparities. For instance, in low-SDI regions, limited healthcare resources impede timely surgical interventions for PAH-CHD. Consequently, many pediatric patients develop severe pulmonary hypertension, including conditions such as Eisenmenger syndrome, greatly amplifying disease burden and mortality risk (28). In contrast, high-income regions, equipped with advanced pediatric cardiac surgical facilities, can perform timely interventions soon after birth, markedly reducing the progression of PAH. IPAH appears more frequently in high-SDI regions, likely reflecting enhanced diagnostic capabilities and increased clinical awareness. Robust pediatric cardiopulmonary specialty care systems in these regions facilitate early identification and diagnosis of IPAH, elevating its proportion among total PAH cases. Additionally, children in these areas benefit from prompt access to advanced pharmacological therapies, such as targeted therapies, significantly improving survival rates and reducing mortality (29). Developmental PAH predominantly emerges in regions with superior healthcare infrastructure, correlating with increased survival rates among premature infants and advancements in neonatal intensive care unit technologies. Due to the complexity and high medical costs associated with diagnosing and treating developmental PAH, its epidemiological characteristics remain inadequately captured in low-SDI regions.

Collectively, the significant correlation between pediatric PAH mortality and SDI is not coincidental; rather, it is the outcome of combined differences in etiology distribution and healthcare resource accessibility influenced by varied socioeconomic contexts. Hence, strengthening pediatric cardiac disease screening, diagnosis, and treatment capacities in low-SDI regions—especially enhancing early surgical intervention rates for congenital heart diseases—is critical to narrowing global disparities in pediatric PAH burdens. Furthermore, fostering international collaboration to standardize diagnosis and treatment approaches for idiopathic and developmental PAH and promoting equitable access to targeted pharmacological therapies are essential strategies for improving global pediatric PAH prognosis and disease burden.

In conclusion, despite considerable progress in reducing pediatric PAH mortality and morbidity globally, significant healthcare inequities persist, particularly affecting low-SDI regions. Continued efforts to enhance healthcare accessibility, coupled with targeted interventions, are necessary to ensure equitable and effective PAH treatment for all children, regardless of geographical location. Such initiatives are pivotal for achieving global health equity and meeting Sustainable Development Goals.

### Limitations

This analysis inherits several methodological constraints intrinsic to GBD modelling, particularly salient for rare pediatric conditions such as PAH. First, primary data for PAH are sparse and geographically uneven: many low- and middle-income countries contribute few or no direct observations, compelling GBD to rely on Bayesian hierarchies and multiple-imputation procedures to borrow strength across covariates, locations, and time (13, 30). Although these techniques represent the current standard, they risk perpetuating undetected biases, and their 95% UIs may fail to adequately account for potential errors, particularly in regions with limited diagnostic infrastructure. Second, the GBD cause hierarchy aggregates all forms of PAH into a single category, precluding stratification by etiology (e.g., PAH secondary to congenital heart disease, idiopathic PAH, or developmentally mediated PAH). Because the necessary subtype-specific data were not available, our results reflect an averaged signal across heterogeneous phenotypes that differ in pathogeny, prognosis, and treatment response. This unavoidable aggregation limits the clinical granularity of the findings and should be considered when extrapolating the estimates to policy or practice.

## Conclusions

The burden of pediatric PAH in 2021—and its trajectory since 1990—varies sharply by SDI quintile and region. Progress in high SDI settings contrasts with persistent or rising burden in low SDI regions, reflecting unequal access to diagnosis and care. Closing this gap requires: (1) universal neonatal and early childhood screening; (2) regional PAH registries with open data sharing; and (3) pooled procurement and capacity building to secure PAH specific drugs and multidisciplinary care. Implementing these measures would translate therapeutic advances into equitable health gains worldwide.

## Data Availability

The datasets presented in this study can be found in online repositories. The names of the repository/repositories and accession number(s) can be found in the article/Supplementary Material.

## Supplementary material

Supplementary Figure S1 Prevalence, Mortality, and Disability-Adjusted Life Year (DALY) Cases of Pediatric Pulmonary Arterial Hypertension Across 204 Countries and Territories. (A) Prevalent cases. (B) Death cases. (C) DALYs cases. DALYs=disability-adjusted life-years.

Supplementary Figure S2 The Prevalence, Mortality, and DALY Rates of Pediatric Pulmonary Arterial Hypertension in 204 Countries and Territories. (A) Disease burden based on Prevalence rate. (B) Disease burden based on Mortality rate. (C) Disease burden based on DALY rate. DALY=Disability-Adjusted Life Year.

Supplementary Figure S3 Prevalence, Mortality, and DALY Rates of Pediatric Pulmonary Arterial Hypertension Across 204 Countries by Sociodemographic Index (SDI) in 2021. (A) Prevalence rate. (B) Mortality. (C) DALY rate. DALY, disability-adjusted life year; SDI, socio-demographic index.
