# Supplementary figures and images for: Burden of pulmonary arterial hypertension in children globally, regionally, and nationally (1990–2021): results from the global burden of disease study
Source: Front Pediatr. 2025 Jun 30;13:1527281. doi: 10.3389/fped.2025.1527281 (PMC12256471; doi:10.3389/fped.2025.1527281)

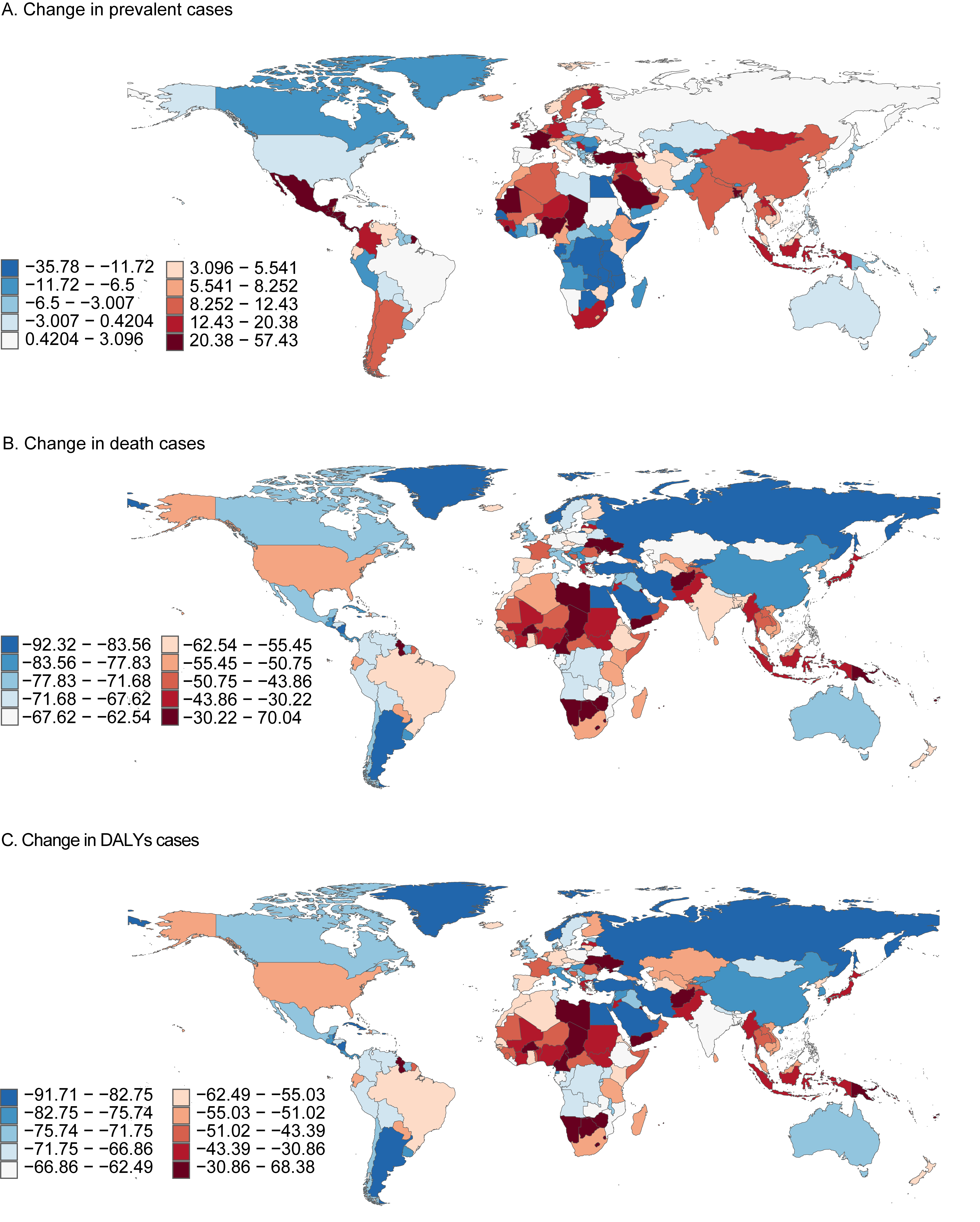

Supplement: Supplementary Figure S1 — Prevalence, Mortality, and Disability-Adjusted Life Year (DALY) Cases of Pediatric Pulmonary Arterial Hypertension Across 204 Countries and Territories. (A) Prevalent cases. (B) Death cases. (C) DALYs cases. DALYs=disability-adjusted life-years. [file Image1.tif]

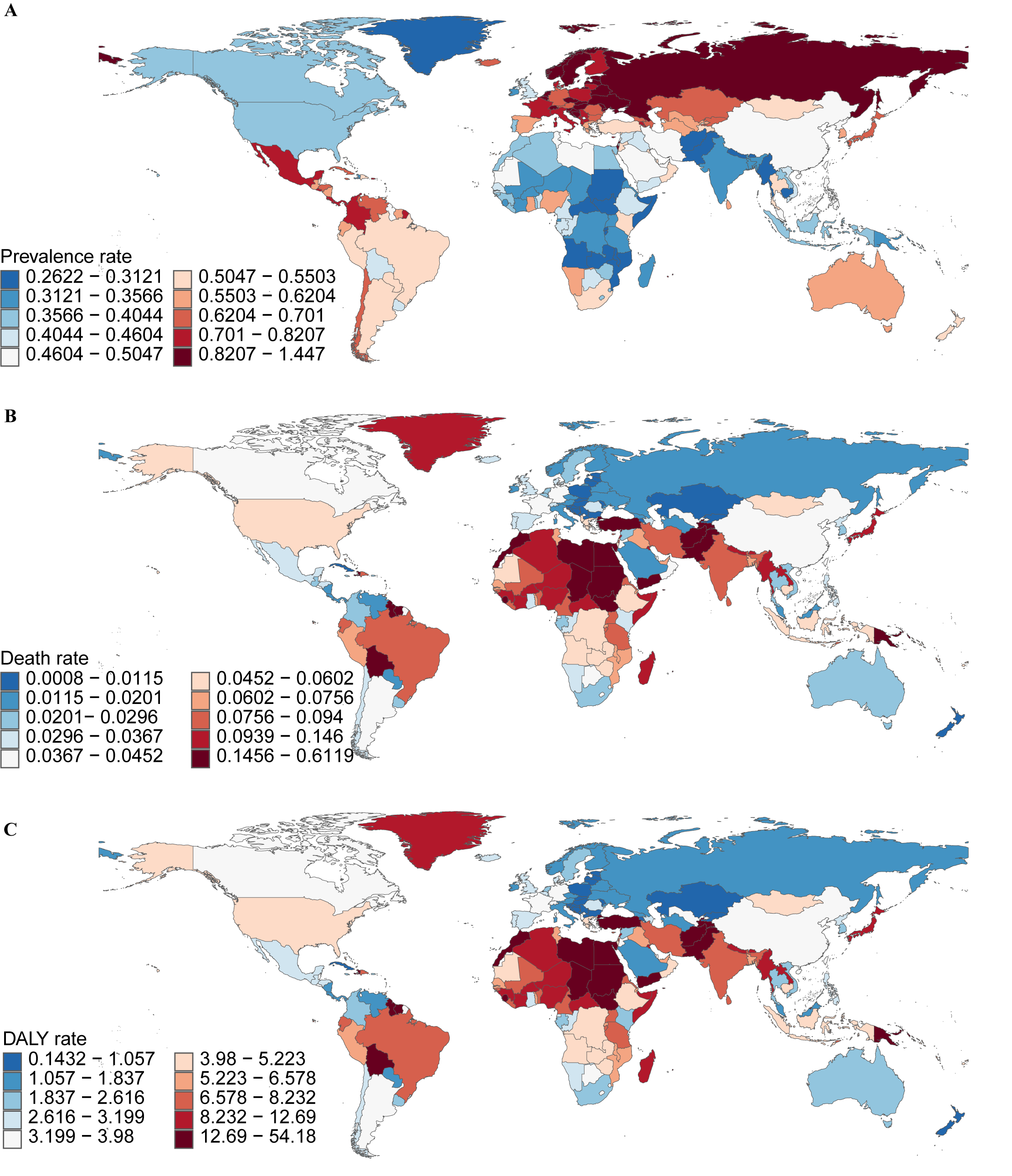

Supplement: Supplementary Figure S2 — The Prevalence, Mortality, and DALY Rates of Pediatric Pulmonary Arterial Hypertension in 204 Countries and Territories. (A) Disease burden based on Prevalence rate. (B) Disease burden based on Mortality rate. (C) Disease burden based on DALY rate. DALY=Disability-Adjusted Life Year. [file Image3.tif]

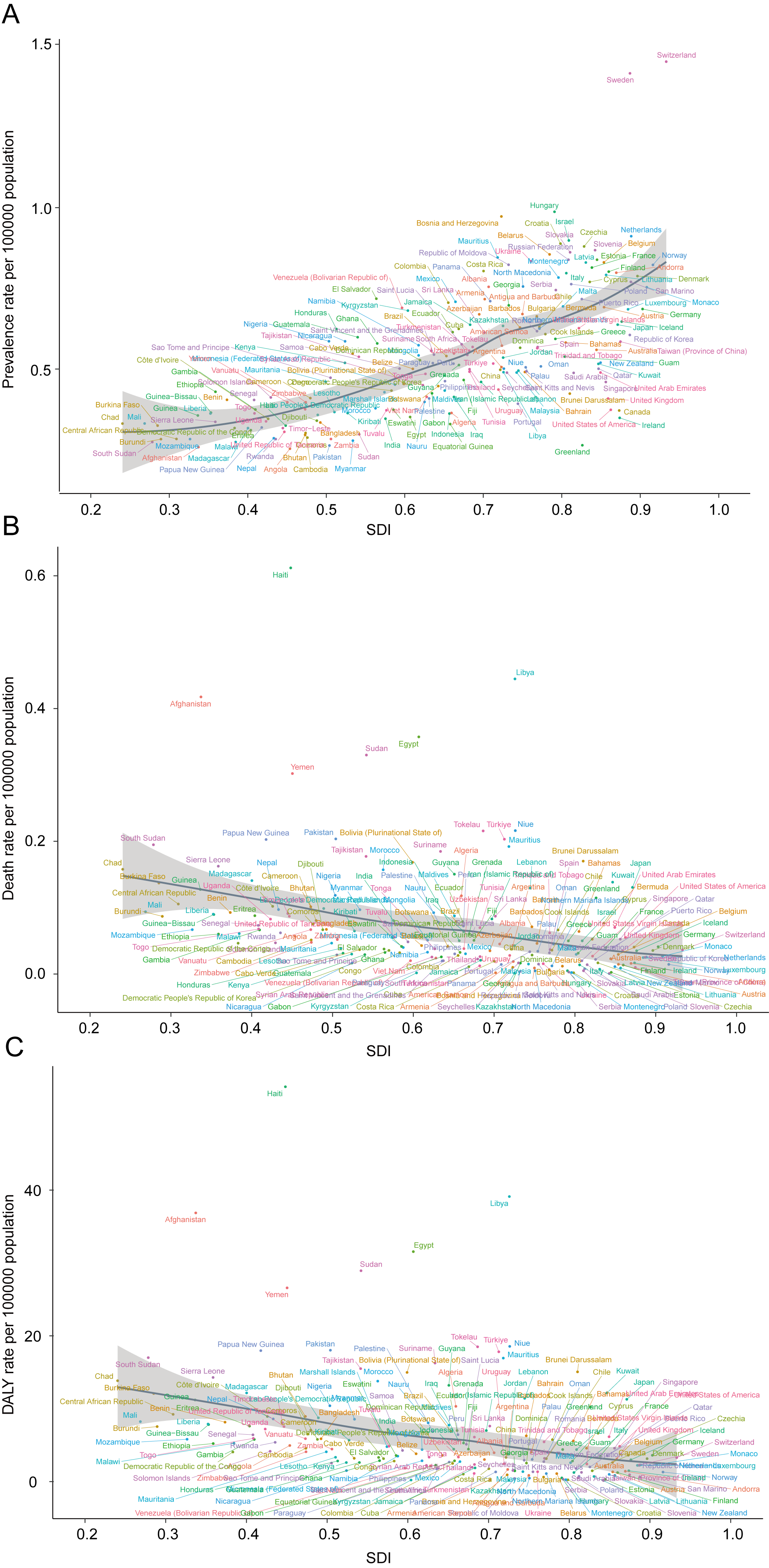

Supplement: Supplementary Figure S3 — Prevalence, Mortality, and DALY Rates of Pediatric Pulmonary Arterial Hypertension Across 204 Countries by Sociodemographic Index (SDI) in 2021. (A) Prevalence rate. (B) Mortality. (C) DALY rate. DALY, disability-adjusted life year; SDI, socio-demographic index. [file Image6.tif]
