# Supplementary material for: Burden of pulmonary arterial hypertension in children globally, regionally, and nationally (1990–2021): results from the global burden of disease study
Source: Front Pediatr. 2025 Jun 30;13:1527281. doi: 10.3389/fped.2025.1527281 (PMC12256471; doi:10.3389/fped.2025.1527281)
Supplement: Supplementary file 4 [file Table1.docx]

Table S1. Death of Pediatric Pulmonary Arterial Hypertension at Global and Regional Levels from 1990 to 2021

|  | 1990 |  |  | 2021 |  |  |  |  |
| --- | --- | --- | --- | --- | --- | --- | --- | --- |
| location | Deaths Cases | Deaths Rate |  | Deaths Cases | Deaths Rate |  | Cases change | EAPC |
| Global | 4049.315(2365.909,5629.395) | 0.233(0.136,0.324) |  | 1714.557(1357.561,2102.268) | 0.085(0.067,0.104) |  | -57.658(-68.782,-38.894) | -2.609(-2.832,-2.385) |
| High SDI | 212.223(194.719,238.577) | 0.114(0.105,0.128) |  | 81.010(73.342,87.785) | 0.047(0.043,0.051) |  | -61.828(-67.232,-57.331) | -2.862(-3.100,-2.623) |
| High-middle SDI | 577.534(397.943,861.931) | 0.211(0.145,0.315) |  | 99.986(82.396,127.021) | 0.043(0.036,0.055) |  | -82.687(-89.058,-71.885) | -4.171(-4.542,-3.799) |
| Middle SDI | 1120.850(777.095,1594.399) | 0.194(0.135,0.276) |  | 279.254(228.275,372.158) | 0.049(0.040,0.066) |  | -75.086(-83.728,-59.147) | -3.360(-3.747,-2.971) |
| Low-middle SDI | 1578.744(700.350,2225.781) | 0.334(0.148,0.471) |  | 676.279(479.768,879.709) | 0.117(0.083,0.152) |  | -57.163(-68.238,-29.249) | -2.784(-2.989,-2.579) |
| Low SDI | 556.550(309.216,1005.989) | 0.243(0.135,0.439) |  | 575.914(420.092,796.751) | 0.125(0.091,0.173) |  | 3.479(-25.258,53.603) | -1.871(-2.010,-1.732) |
| Regions |  |  |  |  |  |  |  |  |
| Andean Latin America | 41.422(21.409,66.124) | 0.279(0.144,0.445) |  | 16.770(12.448,21.436) | 0.093(0.069,0.118) |  | -59.515(-74.832,-21.225) | -3.008(-3.253,-2.762) |
| Australasia | 3.661(2.882,4.546) | 0.080(0.063,0.099) |  | 1.203(0.978,1.480) | 0.021(0.017,0.026) |  | -67.134(-75.618,-56.050) | -3.871(-4.522,-3.215) |
| Caribbean | 54.511(22.102,93.393) | 0.478(0.194,0.818) |  | 31.291(12.014,56.456) | 0.272(0.104,0.491) |  | -42.597(-61.922,-18.685) | -1.779(-1.965,-1.592) |
| Central Asia | 20.027(14.343,27.894) | 0.080(0.057,0.112) |  | 12.034(9.154,15.799) | 0.043(0.033,0.057) |  | -39.911(-58.394,-11.828) | -1.381(-1.677,-1.084) |
| Central Europe | 10.402(8.832,12.056) | 0.035(0.030,0.041) |  | 2.247(1.953,2.943) | 0.013(0.011,0.017) |  | -78.394(-82.752,-73.704) | -3.021(-3.204,-2.839) |
| Central Latin America | 70.217(59.000,86.725) | 0.109(0.092,0.135) |  | 17.936(13.949,22.944) | 0.028(0.022,0.036) |  | -74.457(-81.876,-66.013) | -4.311(-4.781,-3.837) |
| Central Sub-Saharan Africa | 40.872(16.964,93.327) | 0.162(0.067,0.369) |  | 29.673(19.172,50.708) | 0.051(0.033,0.086) |  | -27.400(-58.192,44.890) | -3.330(-3.682,-2.976) |
| East Asia | 641.715(430.925,949.581) | 0.195(0.131,0.288) |  | 99.329(60.613,153.151) | 0.037(0.023,0.057) |  | -84.521(-92.854,-72.510) | -3.653(-4.314,-2.987) |
| Eastern Europe | 55.325(52.057,58.449) | 0.108(0.101,0.114) |  | 6.483(5.918,6.993) | 0.018(0.017,0.020) |  | -88.282(-89.487,-87.214) | -5.320(-5.987,-4.649) |
| Eastern Sub-Saharan Africa | 170.208(72.615,402.380) | 0.188(0.080,0.444) |  | 137.977(80.951,256.432) | 0.077(0.045,0.144) |  | -18.937(-48.186,39.514) | -2.719(-2.822,-2.615) |
| High-income Asia Pacific | 65.247(60.162,72.211) | 0.185(0.171,0.205) |  | 22.280(20.256,24.380) | 0.099(0.090,0.109) |  | -65.853(-70.449,-61.374) | -2.518(-2.986,-2.047) |
| High-income North America | 76.304(69.545,85.233) | 0.124(0.113,0.138) |  | 35.179(31.660,38.891) | 0.054(0.048,0.059) |  | -53.896(-60.557,-47.120) | -2.760(-2.988,-2.531) |
| North Africa and Middle East | 1303.515(555.577,1989.220) | 0.928(0.395,1.416) |  | 400.039(296.496,509.201) | 0.218(0.162,0.278) |  | -69.311(-81.204,-43.871) | -3.721(-4.036,-3.406) |
| Oceania | 4.627(2.584,7.830) | 0.173(0.096,0.292) |  | 8.803(5.078,15.452) | 0.173(0.100,0.304) |  | 90.244(24.662,181.463) | 0.162(-0.005,0.328) |
| South Asia | 986.777(391.366,1442.164) | 0.228(0.090,0.333) |  | 508.772(297.824,784.851) | 0.100(0.059,0.155) |  | -48.441(-61.878,-19.346) | -2.234(-2.383,-2.084) |
| Southeast Asia | 145.345(78.697,354.143) | 0.085(0.046,0.207) |  | 79.376(52.697,156.709) | 0.046(0.031,0.091) |  | -45.388(-63.993,-15.173) | -1.788(-1.913,-1.664) |
| Southern Latin America | 40.521(35.788,46.231) | 0.271(0.240,0.310) |  | 5.884(4.934,6.921) | 0.041(0.034,0.048) |  | -85.478(-88.639,-81.774) | -5.618(-5.895,-5.339) |
| Southern Sub-Saharan Africa | 8.778(6.267,11.766) | 0.042(0.030,0.057) |  | 7.422(5.459,9.800) | 0.031(0.023,0.041) |  | -15.441(-40.438,22.774) | -0.287(-0.666,0.093) |
| Tropical Latin America | 94.187(82.000,107.954) | 0.176(0.153,0.201) |  | 38.474(31.132,46.846) | 0.077(0.062,0.093) |  | -59.151(-68.772,-48.988) | -2.125(-2.883,-1.361) |
| Western Europe | 66.739(60.069,76.907) | 0.094(0.085,0.108) |  | 22.932(20.690,25.132) | 0.034(0.030,0.037) |  | -65.640(-70.840,-60.959) | -3.077(-3.499,-2.654) |
| Western Sub-Saharan Africa | 148.914(58.550,366.289) | 0.169(0.067,0.417) |  | 230.452(150.915,373.486) | 0.107(0.070,0.174) |  | 54.755(2.245,175.151) | -1.113(-1.385,-0.840) |

EAPC=estimated annual percentage change.
