# Supplementary material for: Burden of pulmonary arterial hypertension in children globally, regionally, and nationally (1990–2021): results from the global burden of disease study
Source: Front Pediatr. 2025 Jun 30;13:1527281. doi: 10.3389/fped.2025.1527281 (PMC12256471; doi:10.3389/fped.2025.1527281)
Supplement: Supplementary file 5 [file Table2.docx]

Table S2. DALYs of Pediatric Pulmonary Arterial Hypertension at Global and Regional Levels from 1990 to 2021

|  | 1990 | |  | 2021 | |  | 1990-2021 |  |
| --- | --- | --- | --- | --- | --- | --- | --- | --- |
| Location | DALYs cases | DALYs rate |  | DALYs cases | DALYs rate |  | Cases change | EAPC |
| Global | 358741.23(209032.75,498948.49) | 20.63(12.02,28.69) |  | 151098.12(119751.94,185128.35) | 7.51(5.95,9.20) |  | -63.59(-73.18,-47.38) | -2.62(-2.84,-2.40) |
| High SDI | 18672.73(17129.88,20967.02) | 10.05(9.22,11.28) |  | 7122.99(6443.36,7726.35) | 4.13(3.73,4.48) |  | -58.92(-64.79,-54.09) | -2.86(-3.10,-2.62) |
| High-middle SDI | 51059.72(35119.50,76298.49) | 18.66(12.83,27.88) |  | 8831.56(7284.48,11214.69) | 3.83(3.15,4.86) |  | -79.50(-87.07,-66.81) | -4.17(-4.54,-3.80) |
| Middle SDI | 99163.75(68836.02,141146.78) | 17.18(11.93,24.45) |  | 24614.71(20109.89,32781.54) | 4.34(3.55,5.78) |  | -74.72(-83.46,-58.55) | -3.36(-3.75,-2.97) |
| Low-middle SDI | 140367.83(62206.70,197848.21) | 29.73(13.18,41.91) |  | 59749.55(42265.85,77882.00) | 10.30(7.29,13.43) |  | -65.34(-74.29,-42.68) | -2.80(-3.01,-2.60) |
| Low SDI | 49174.84(27278.47,88786.62) | 21.48(11.92,38.79) |  | 50592.43(36915.79,69903.99) | 10.99(8.02,15.19) |  | -48.83(-62.93,-24.03) | -1.89(-2.03,-1.75) |
| Regions |  |  |  |  |  |  |  |  |
| Andean Latin America | 3683.49(1905.99,5884.10) | 24.80(12.83,39.62) |  | 1491.10(1107.48,1907.22) | 8.24(6.12,10.54) |  | -66.77(-79.30,-35.49) | -3.01(-3.25,-2.77) |
| Australasia | 322.19(253.01,400.14) | 7.03(5.52,8.73) |  | 107.39(87.40,132.29) | 1.87(1.52,2.31) |  | -73.33(-80.20,-64.46) | -3.82(-4.46,-3.17) |
| Caribbean | 4829.67(1958.31,8266.29) | 42.32(17.16,72.43) |  | 2771.97(1067.98,5000.74) | 24.09(9.28,43.47) |  | -43.07(-62.30,-19.49) | -1.78(-1.96,-1.59) |
| Central Asia | 1763.81(1265.98,2463.89) | 7.06(5.07,9.86) |  | 1058.40(802.69,1392.60) | 3.82(2.90,5.03) |  | -45.81(-62.53,-20.44) | -1.37(-1.67,-1.07) |
| Central Europe | 909.02(777.06,1050.41) | 3.08(2.64,3.56) |  | 200.79(175.23,259.89) | 1.13(0.99,1.47) |  | -63.21(-70.43,-55.37) | -2.95(-3.13,-2.78) |
| Central Latin America | 6242.78(5245.58,7703.92) | 9.70(8.15,11.97) |  | 1611.08(1257.55,2054.50) | 2.54(1.98,3.24) |  | -73.83(-81.34,-65.42) | -4.29(-4.75,-3.82) |
| Central Sub-Saharan Africa | 3611.01(1497.42,8225.00) | 14.27(5.92,32.51) |  | 2595.06(1685.07,4446.53) | 4.42(2.87,7.58) |  | -69.02(-82.05,-38.30) | -3.36(-3.72,-3.01) |
| East Asia | 56910.23(38248.18,84313.56) | 17.25(11.60,25.56) |  | 8801.20(5431.83,13437.71) | 3.29(2.03,5.03) |  | -80.92(-91.14,-66.24) | -3.65(-4.31,-2.98) |
| Eastern Europe | 4835.48(4536.06,5108.67) | 9.40(8.81,9.93) |  | 580.86(530.05,625.07) | 1.64(1.50,1.76) |  | -82.56(-84.23,-80.96) | -5.24(-5.89,-4.58) |
| Eastern Sub-Saharan Africa | 14907.04(6298.06,35336.59) | 16.46(6.95,39.02) |  | 11979.17(6901.35,22386.64) | 6.71(3.87,12.55) |  | -59.21(-73.84,-29.68) | -2.74(-2.85,-2.64) |
| High-income Asia Pacific | 5677.40(5233.05,6281.66) | 16.13(14.87,17.85) |  | 1930.36(1753.68,2116.81) | 8.61(7.82,9.44) |  | -46.63(-53.83,-39.62) | -2.53(-3.01,-2.05) |
| High-income North America | 6764.14(6166.50,7554.86) | 10.97(10.00,12.25) |  | 3098.53(2793.04,3421.22) | 4.72(4.26,5.21) |  | -56.94(-63.16,-50.65) | -2.78(-3.00,-2.55) |
| North Africa and Middle East | 115496.02(49092.32,176539.36) | 82.21(34.94,125.66) |  | 35166.90(26052.24,44742.32) | 19.18(14.21,24.41) |  | -76.67(-85.70,-57.19) | -3.74(-4.05,-3.42) |
| Oceania | 411.47(230.41,696.75) | 15.35(8.60,26.00) |  | 780.10(449.43,1370.21) | 15.35(8.85,26.97) |  | -0.00(-34.46,48.45) | 0.15(-0.01,0.32) |
| South Asia | 87898.03(35012.79,128766.85) | 20.28(8.08,29.71) |  | 45121.64(26371.51,69880.78) | 8.90(5.20,13.78) |  | -56.12(-67.58,-31.45) | -2.25(-2.40,-2.10) |
| Southeast Asia | 12879.43(6958.85,31484.22) | 7.54(4.08,18.44) |  | 7026.43(4672.07,13831.77) | 4.07(2.71,8.01) |  | -46.05(-64.47,-16.39) | -1.80(-1.92,-1.67) |
| Southern Latin America | 3578.09(3161.93,4084.02) | 23.97(21.18,27.36) |  | 513.03(429.48,603.97) | 3.54(2.96,4.17) |  | -85.24(-88.45,-81.47) | -5.65(-5.93,-5.37) |
| Southern Sub-Saharan Africa | 765.57(546.20,1028.40) | 3.70(2.64,4.97) |  | 640.62(473.46,843.48) | 2.66(1.97,3.50) |  | -28.06(-49.16,4.22) | -0.30(-0.68,0.08) |
| Tropical Latin America | 8279.66(7219.73,9495.78) | 15.44(13.47,17.71) |  | 3368.93(2728.07,4106.83) | 6.71(5.44,8.18) |  | -56.54(-66.84,-45.59) | -2.14(-2.89,-1.38) |
| Western Europe | 5853.61(5269.75,6740.24) | 8.24(7.42,9.49) |  | 2027.85(1830.18,2217.80) | 2.98(2.69,3.26) |  | -63.88(-69.41,-58.81) | -3.04(-3.46,-2.63) |
| Western Sub-Saharan Africa | 13123.10(5132.30,32311.57) | 14.93(5.84,36.77) |  | 20226.72(13223.05,32870.77) | 9.42(6.16,15.31) |  | -36.93(-58.26,12.38) | -1.12(-1.40,-0.85) |

DALYs= disability-adjusted life-years; EAPC=estimated annual percentage change.
