# Supplementary material for: Burden of pulmonary arterial hypertension in children globally, regionally, and nationally (1990–2021): results from the global burden of disease study
Source: Front Pediatr. 2025 Jun 30;13:1527281. doi: 10.3389/fped.2025.1527281 (PMC12256471; doi:10.3389/fped.2025.1527281)
Supplement: Supplementary file 6 [file Table3.docx]

Table S3 Prevalence of Pediatric Pulmonary Arterial Hypertension at the national level

| location | 1990 | |  | 2021 | |  | 1990-2021 | |
| --- | --- | --- | --- | --- | --- | --- | --- | --- |
|  | prevalence case | prevalence rate |  | prevalence case | prevalence rate |  | Cases change | EAPC |
| Afghanistan | 11.20(7.57,15.35) | 0.26(0.18,0.36) |  | 37.92(25.52,52.65) | 0.27(0.18,0.37) |  | 238.70(205.33,272.08) | 0.413210794 |
| Albania | 8.96(6.55,11.67) | 0.80(0.59,1.04) |  | 3.36(2.40,4.43) | 0.76(0.54,1.00) |  | -62.48(-65.77,-58.89) | -0.139204462 |
| Algeria | 36.47(25.18,48.96) | 0.34(0.23,0.46) |  | 49.44(34.52,67.01) | 0.37(0.26,0.50) |  | 35.55(23.40,48.62) | 0.071888623 |
| American Samoa | 0.10(0.07,0.13) | 0.52(0.37,0.69) |  | 0.08(0.06,0.11) | 0.58(0.41,0.77) |  | -17.13(-24.88,-9.83) | 0.647999817 |
| Andorra | 0.07(0.05,0.09) | 0.74(0.53,0.96) |  | 0.08(0.06,0.10) | 0.80(0.59,1.03) |  | 16.56(8.14,27.95) | 0.405551214 |
| Angola | 13.98(9.61,19.23) | 0.30(0.20,0.41) |  | 39.98(26.75,55.75) | 0.26(0.18,0.37) |  | 185.87(152.67,218.96) | -0.389654512 |
| Antigua and Barbuda | 0.13(0.09,0.16) | 0.69(0.51,0.90) |  | 0.11(0.08,0.15) | 0.67(0.49,0.87) |  | -9.13(-16.95,-1.83) | -0.061539408 |
| Argentina | 47.19(33.43,61.68) | 0.47(0.33,0.61) |  | 52.15(37.91,68.07) | 0.51(0.37,0.67) |  | 10.50(1.03,18.60) | 0.270041917 |
| Armenia | 7.21(5.32,9.22) | 0.69(0.51,0.88) |  | 4.27(3.13,5.56) | 0.72(0.53,0.94) |  | -40.84(-45.78,-35.36) | -0.190986214 |
| Australia | 21.36(15.42,27.31) | 0.56(0.41,0.72) |  | 26.82(19.68,34.86) | 0.56(0.41,0.73) |  | 25.57(15.56,35.83) | -0.061735785 |
| Austria | 8.84(6.42,11.39) | 0.66(0.48,0.84) |  | 9.02(6.64,11.57) | 0.70(0.51,0.89) |  | 2.00(-5.63,10.74) | 0.209736961 |
| Azerbaijan | 12.68(9.12,16.44) | 0.52(0.38,0.68) |  | 15.90(11.68,20.42) | 0.67(0.49,0.87) |  | 25.45(14.66,38.52) | 0.61695525 |
| Bahamas | 0.52(0.38,0.67) | 0.64(0.47,0.83) |  | 0.48(0.34,0.63) | 0.59(0.42,0.78) |  | -8.31(-18.02,2.54) | -0.160632026 |
| Bahrain | 0.76(0.54,0.99) | 0.46(0.33,0.61) |  | 1.47(1.06,1.94) | 0.50(0.36,0.65) |  | 94.99(78.21,111.98) | 0.180268273 |
| Bangladesh | 109.74(75.03,152.19) | 0.22(0.15,0.31) |  | 156.71(110.15,212.10) | 0.34(0.24,0.46) |  | 42.80(28.48,58.00) | 1.515337175 |
| Barbados | 0.40(0.29,0.52) | 0.65(0.47,0.83) |  | 0.30(0.22,0.39) | 0.63(0.46,0.83) |  | -25.92(-32.45,-19.57) | -0.064065916 |
| Belarus | 20.56(15.39,26.46) | 0.86(0.64,1.10) |  | 13.52(10.06,17.34) | 0.86(0.64,1.10) |  | -34.22(-39.92,-28.56) | -0.329485147 |
| Belgium | 13.80(9.99,17.65) | 0.76(0.55,0.98) |  | 15.92(11.66,20.37) | 0.83(0.61,1.07) |  | 15.35(7.16,23.29) | 0.273662302 |
| Belize | 0.39(0.28,0.52) | 0.47(0.34,0.63) |  | 0.62(0.43,0.85) | 0.51(0.35,0.69) |  | 60.46(44.98,76.57) | 0.042613595 |
| Benin | 9.62(6.63,13.14) | 0.40(0.27,0.54) |  | 25.05(17.09,33.95) | 0.41(0.28,0.56) |  | 160.43(134.10,189.85) | 0.942120433 |
| Bermuda | 0.08(0.06,0.10) | 0.66(0.49,0.84) |  | 0.06(0.04,0.07) | 0.68(0.50,0.89) |  | -26.75(-32.81,-20.47) | 0.06038229 |
| Bhutan | 0.88(0.61,1.21) | 0.34(0.23,0.46) |  | 0.57(0.39,0.78) | 0.30(0.21,0.42) |  | -35.11(-41.86,-28.16) | -0.418695221 |
| Bolivia (Plurinational State of) | 12.60(8.74,16.96) | 0.47(0.33,0.63) |  | 16.05(11.05,22.02) | 0.46(0.32,0.63) |  | 27.42(16.60,38.87) | -0.173165101 |
| Bosnia and Herzegovina | 10.88(8.19,14.04) | 0.99(0.75,1.28) |  | 4.77(3.47,6.08) | 0.97(0.71,1.24) |  | -56.15(-59.96,-52.39) | -0.16920373 |
| Botswana | 3.04(2.05,4.17) | 0.51(0.35,0.71) |  | 3.17(2.14,4.38) | 0.45(0.31,0.63) |  | 4.26(-4.99,14.61) | -0.434412874 |
| Brazil | 278.59(198.48,368.12) | 0.54(0.38,0.71) |  | 263.02(188.84,346.81) | 0.55(0.39,0.72) |  | -5.59(-7.92,-3.22) | -0.042942666 |
| Brunei Darussalam | 0.38(0.27,0.50) | 0.42(0.30,0.55) |  | 0.41(0.29,0.54) | 0.43(0.31,0.57) |  | 7.54(0.09,16.63) | 0.272680816 |
| Bulgaria | 13.37(10.10,16.80) | 0.77(0.58,0.97) |  | 6.51(4.75,8.49) | 0.67(0.49,0.87) |  | -51.26(-56.03,-46.72) | -0.375907096 |
| Burkina Faso | 14.99(10.16,20.67) | 0.32(0.22,0.44) |  | 34.53(23.83,46.41) | 0.33(0.23,0.45) |  | 130.31(105.38,160.29) | 0.369684869 |
| Burundi | 10.42(7.19,14.31) | 0.40(0.27,0.55) |  | 17.06(11.39,23.57) | 0.29(0.19,0.40) |  | 63.71(48.96,80.10) | -1.369833603 |
| Cabo Verde | 0.65(0.44,0.88) | 0.41(0.28,0.56) |  | 0.79(0.57,1.06) | 0.55(0.40,0.74) |  | 22.33(11.90,34.09) | 1.183086177 |
| Cambodia | 13.71(9.26,18.77) | 0.29(0.20,0.40) |  | 15.88(10.81,21.76) | 0.31(0.21,0.43) |  | 15.86(5.67,28.63) | 0.1740103 |
| Cameroon | 19.23(13.26,25.96) | 0.39(0.27,0.53) |  | 56.17(38.38,75.92) | 0.42(0.28,0.56) |  | 192.03(163.41,226.42) | 0.614553936 |
| Canada | 23.71(17.45,30.66) | 0.41(0.30,0.53) |  | 23.40(16.73,30.95) | 0.38(0.27,0.50) |  | -1.32(-9.75,8.60) | -0.363708504 |
| Central African Republic | 3.75(2.54,5.17) | 0.31(0.21,0.42) |  | 6.67(4.56,9.24) | 0.29(0.20,0.40) |  | 77.77(62.86,95.84) | -0.225925514 |
| Chad | 7.74(5.34,10.81) | 0.26(0.18,0.37) |  | 30.58(20.80,41.82) | 0.34(0.23,0.46) |  | 294.87(252.34,338.15) | 1.247932306 |
| Chile | 23.15(16.91,29.68) | 0.58(0.43,0.75) |  | 23.17(17.07,30.02) | 0.63(0.47,0.82) |  | 0.11(-7.90,8.17) | 0.006050124 |
| China | 1436.58(1006.69,1918.66) | 0.45(0.32,0.60) |  | 1308.02(920.25,1730.26) | 0.50(0.35,0.67) |  | -8.95(-10.81,-7.04) | 0.226616271 |
| Colombia | 72.33(51.88,94.70) | 0.62(0.44,0.81) |  | 75.27(55.08,98.77) | 0.71(0.52,0.93) |  | 4.06(-3.41,12.14) | 0.265065757 |
| Comoros | 0.72(0.50,0.97) | 0.34(0.23,0.45) |  | 0.71(0.48,1.00) | 0.30(0.20,0.42) |  | -0.82(-10.35,11.14) | -0.60030469 |
| Congo | 5.22(3.56,7.04) | 0.50(0.34,0.67) |  | 8.49(5.82,11.63) | 0.44(0.30,0.60) |  | 62.61(47.68,80.56) | -0.30274645 |
| Cook Islands | 0.04(0.03,0.05) | 0.61(0.44,0.79) |  | 0.02(0.02,0.03) | 0.61(0.44,0.81) |  | -41.85(-46.40,-36.92) | 0.335848573 |
| Costa Rica | 7.51(5.38,9.83) | 0.67(0.48,0.87) |  | 8.20(5.99,10.65) | 0.81(0.59,1.05) |  | 9.12(0.70,19.74) | 0.322921299 |
| Croatia | 21.77(14.99,29.93) | 0.38(0.26,0.52) |  | 40.92(27.23,54.94) | 0.35(0.24,0.47) |  | -41.33(-45.80,-36.41) | -0.081980671 |
| Cuba | 9.06(6.79,11.72) | 0.92(0.69,1.19) |  | 5.31(3.92,6.82) | 0.89(0.66,1.14) |  | -28.56(-34.43,-22.80) | -0.138324287 |
| Cyprus | 15.65(11.62,20.40) | 0.62(0.46,0.81) |  | 11.18(8.13,14.71) | 0.63(0.46,0.83) |  | 24.18(12.85,38.23) | -0.11828676 |
| Czechia | 1.36(1.00,1.76) | 0.69(0.51,0.89) |  | 1.69(1.25,2.15) | 0.77(0.57,0.98) |  | -26.89(-32.79,-20.85) | 0.443114693 |
| C么te d'Ivoire | 20.69(15.36,26.49) | 0.94(0.70,1.20) |  | 15.13(11.05,19.47) | 0.88(0.64,1.13) |  | 87.97(69.33,109.93) | -0.354817428 |
| Democratic People's Republic of Korea | 25.71(18.43,33.54) | 0.43(0.31,0.56) |  | 22.11(15.27,29.71) | 0.46(0.32,0.62) |  | -14.03(-20.63,-5.28) | 0.318315489 |
| Democratic Republic of the Congo | 89.72(61.65,120.44) | 0.51(0.35,0.68) |  | 123.67(83.72,169.74) | 0.33(0.22,0.45) |  | 37.84(23.25,53.11) | -1.291223087 |
| Denmark | 5.84(4.19,7.66) | 0.66(0.47,0.87) |  | 7.54(5.61,9.58) | 0.79(0.59,1.00) |  | 29.15(16.87,45.42) | 0.772846253 |
| Djibouti | 0.56(0.38,0.77) | 0.32(0.22,0.44) |  | 1.44(0.98,1.96) | 0.35(0.24,0.47) |  | 155.39(133.67,178.90) | -0.066367088 |
| Dominica | 0.12(0.08,0.16) | 0.49(0.34,0.65) |  | 0.08(0.05,0.11) | 0.57(0.40,0.78) |  | -35.23(-40.86,-29.00) | 0.412854392 |
| Dominican Republic | 15.31(10.83,20.06) | 0.57(0.40,0.74) |  | 15.66(11.06,21.04) | 0.53(0.38,0.72) |  | 2.27(-7.11,10.75) | -0.242330309 |
| Ecuador | 22.98(16.39,30.42) | 0.59(0.42,0.79) |  | 31.39(22.17,41.42) | 0.62(0.44,0.82) |  | 36.57(24.95,50.03) | 0.133135811 |
| Egypt | 117.65(79.90,160.20) | 0.53(0.36,0.72) |  | 132.42(90.96,181.75) | 0.36(0.25,0.49) |  | 12.55(2.37,24.75) | -1.784767117 |
| El Salvador | 11.24(7.83,15.40) | 0.52(0.36,0.71) |  | 13.12(9.65,17.00) | 0.72(0.53,0.93) |  | 16.70(3.15,31.13) | 1.185527955 |
| Equatorial Guinea | 0.61(0.42,0.84) | 0.31(0.21,0.43) |  | 1.98(1.33,2.73) | 0.34(0.23,0.47) |  | 223.33(191.79,258.88) | 0.122098951 |
| Eritrea | 6.04(4.13,8.37) | 0.38(0.26,0.53) |  | 8.05(5.42,11.15) | 0.32(0.21,0.44) |  | 33.24(18.35,48.24) | -0.693432711 |
| Estonia | 2.92(2.19,3.74) | 0.84(0.63,1.07) |  | 1.82(1.37,2.30) | 0.84(0.63,1.06) |  | -37.85(-43.03,-32.86) | -0.268990312 |
| Eswatini | 1.43(0.97,1.95) | 0.37(0.25,0.51) |  | 1.57(1.06,2.17) | 0.38(0.26,0.53) |  | 10.32(1.65,20.81) | 0.061807992 |
| Ethiopia | 98.41(68.46,132.48) | 0.40(0.28,0.54) |  | 193.57(133.80,262.83) | 0.44(0.30,0.59) |  | 96.70(87.50,106.85) | -0.237278856 |
| Fiji | 1.48(1.02,1.97) | 0.52(0.36,0.70) |  | 1.29(0.87,1.72) | 0.47(0.32,0.63) |  | -12.69(-19.49,-4.56) | 0.243549317 |
| Finland | 6.52(4.71,8.48) | 0.68(0.49,0.88) |  | 6.81(5.03,8.65) | 0.80(0.59,1.02) |  | 4.42(-4.34,14.77) | 0.673959397 |
| France | 77.63(54.89,103.34) | 0.66(0.47,0.88) |  | 94.79(70.07,120.87) | 0.82(0.60,1.04) |  | 22.10(11.36,34.53) | 0.650929849 |
| Gabon | 2.28(1.56,3.14) | 0.56(0.38,0.77) |  | 2.76(1.89,3.78) | 0.43(0.30,0.59) |  | 21.00(10.98,33.93) | -0.602737137 |
| Gambia | 1.91(1.32,2.58) | 0.41(0.29,0.56) |  | 3.80(2.58,5.16) | 0.38(0.26,0.52) |  | 98.70(80.69,118.84) | 0.129615683 |
| Georgia | 9.52(7.04,12.32) | 0.70(0.51,0.90) |  | 5.16(3.79,6.71) | 0.70(0.51,0.91) |  | -45.83(-50.43,-41.51) | -0.271223383 |
| Germany | 75.32(54.28,99.06) | 0.58(0.42,0.77) |  | 82.53(60.89,105.62) | 0.69(0.51,0.88) |  | 9.56(-0.39,20.39) | 0.25976603 |
| Ghana | 40.71(28.41,53.53) | 0.61(0.42,0.80) |  | 75.29(53.17,100.65) | 0.58(0.41,0.78) |  | 84.94(64.68,103.59) | 0.256929749 |
| Greece | 12.77(9.32,16.60) | 0.63(0.46,0.82) |  | 8.35(6.10,11.11) | 0.60(0.44,0.80) |  | -34.64(-40.54,-28.96) | -0.273681879 |
| Greenland | 0.04(0.03,0.06) | 0.31(0.22,0.41) |  | 0.03(0.02,0.04) | 0.27(0.19,0.37) |  | -26.28(-33.88,-18.36) | -0.398532865 |
| Grenada | 0.18(0.13,0.24) | 0.55(0.40,0.72) |  | 0.11(0.08,0.15) | 0.52(0.36,0.70) |  | -37.93(-43.59,-31.74) | -0.063755942 |
| Guam | 0.23(0.16,0.30) | 0.54(0.38,0.71) |  | 0.20(0.14,0.27) | 0.54(0.38,0.73) |  | -11.87(-19.32,-4.15) | 0.336313003 |
| Guatemala | 18.44(12.58,25.38) | 0.45(0.31,0.63) |  | 29.78(20.85,39.98) | 0.60(0.42,0.81) |  | 61.43(44.43,78.45) | 0.697799676 |
| Guinea | 8.44(5.84,11.62) | 0.31(0.21,0.42) |  | 22.00(15.07,29.99) | 0.36(0.25,0.50) |  | 160.71(132.95,191.22) | 1.046771477 |
| Guinea-Bissau | 1.49(1.00,2.09) | 0.31(0.21,0.43) |  | 3.35(2.28,4.70) | 0.37(0.25,0.52) |  | 124.13(103.02,151.42) | 0.991970534 |
| Guyana | 1.48(1.05,1.99) | 0.50(0.36,0.68) |  | 1.04(0.73,1.44) | 0.49(0.34,0.67) |  | -29.96(-36.05,-23.23) | -0.101898617 |
| Haiti | 9.58(6.63,13.15) | 0.35(0.24,0.48) |  | 14.49(9.77,19.93) | 0.33(0.22,0.46) |  | 51.34(37.12,65.56) | -0.389234887 |
| Honduras | 10.90(7.56,14.80) | 0.49(0.34,0.67) |  | 20.59(14.57,27.35) | 0.63(0.44,0.83) |  | 88.93(72.45,108.34) | 0.543449883 |
| Hungary | 22.51(17.04,29.06) | 1.06(0.80,1.36) |  | 13.71(10.17,17.57) | 0.99(0.73,1.27) |  | -39.08(-43.23,-34.34) | -0.114674443 |
| Iceland | 0.39(0.29,0.50) | 0.62(0.45,0.79) |  | 0.44(0.32,0.56) | 0.65(0.48,0.84) |  | 12.55(4.77,21.60) | 0.197988382 |
| India | 1047.98(718.00,1420.50) | 0.32(0.22,0.44) |  | 1297.57(891.42,1784.07) | 0.35(0.24,0.49) |  | 23.82(20.40,27.09) | 0.277984091 |
| Indonesia | 216.50(149.77,290.90) | 0.32(0.22,0.43) |  | 248.82(171.02,338.76) | 0.37(0.25,0.50) |  | 14.93(10.73,18.59) | 0.456451558 |
| Iran (Islamic Republic of) | 112.22(79.09,148.29) | 0.44(0.31,0.58) |  | 94.15(66.58,124.74) | 0.47(0.33,0.62) |  | -16.11(-17.86,-14.29) | -0.088474159 |
| Iraq | 29.28(20.10,39.34) | 0.36(0.24,0.48) |  | 56.62(39.29,78.10) | 0.42(0.29,0.58) |  | 93.42(76.85,112.65) | 0.375876633 |
| Ireland | 3.07(2.24,4.10) | 0.31(0.23,0.42) |  | 3.56(2.56,4.67) | 0.36(0.26,0.47) |  | 15.67(5.50,27.71) | 0.34491275 |
| Israel | 13.92(10.05,18.16) | 0.91(0.66,1.18) |  | 23.64(17.54,30.38) | 0.90(0.67,1.16) |  | 69.81(56.64,84.60) | 0.014030076 |
| Italy | 70.54(51.42,91.34) | 0.76(0.56,0.99) |  | 60.77(44.96,78.18) | 0.80(0.59,1.03) |  | -13.85(-16.15,-11.47) | 0.267683287 |
| Jamaica | 5.19(3.82,6.68) | 0.62(0.46,0.80) |  | 3.75(2.71,4.82) | 0.64(0.46,0.83) |  | -27.81(-33.56,-22.15) | 0.091798614 |
| Japan | 155.25(115.45,198.51) | 0.67(0.50,0.86) |  | 99.14(73.34,126.82) | 0.64(0.47,0.82) |  | -36.14(-37.26,-34.97) | -0.090517495 |
| Jordan | 8.00(5.82,10.56) | 0.49(0.36,0.65) |  | 19.65(13.77,26.36) | 0.54(0.38,0.73) |  | 145.63(124.70,167.32) | 0.152587044 |
| Kazakhstan | 34.18(24.96,44.88) | 0.66(0.48,0.86) |  | 34.63(25.12,44.98) | 0.64(0.46,0.83) |  | 1.31(-6.34,12.11) | -0.381467616 |
| Kenya | 55.77(38.35,75.72) | 0.50(0.34,0.68) |  | 97.99(67.26,133.82) | 0.52(0.36,0.72) |  | 75.70(71.19,79.81) | -0.117473396 |
| Kiribati | 0.11(0.07,0.15) | 0.37(0.25,0.50) |  | 0.16(0.11,0.22) | 0.37(0.25,0.51) |  | 43.84(30.04,58.27) | 0.340426853 |
| Kuwait | 2.72(1.98,3.54) | 0.49(0.36,0.64) |  | 4.41(3.12,5.81) | 0.52(0.37,0.69) |  | 62.38(51.21,76.15) | 0.180055408 |
| Kyrgyzstan | 10.03(7.28,12.84) | 0.60(0.43,0.77) |  | 15.58(11.46,20.02) | 0.68(0.50,0.88) |  | 55.29(42.66,72.05) | 0.122560828 |
| Lao People's Democratic Republic | 5.65(3.92,7.76) | 0.31(0.21,0.42) |  | 8.40(5.62,11.75) | 0.37(0.24,0.51) |  | 48.70(35.14,61.67) | 0.687055299 |
| Latvia | 4.62(3.43,5.94) | 0.81(0.60,1.04) |  | 2.47(1.85,3.14) | 0.83(0.62,1.06) |  | -46.54(-50.93,-41.65) | -0.158727259 |
| Lebanon | 4.94(3.51,6.54) | 0.47(0.34,0.63) |  | 6.41(4.53,8.53) | 0.50(0.35,0.67) |  | 29.78(20.72,39.93) | 0.148046747 |
| Lesotho | 2.41(1.61,3.30) | 0.35(0.24,0.48) |  | 2.36(1.62,3.36) | 0.37(0.26,0.53) |  | -2.14(-10.89,6.63) | 0.167109749 |
| Liberia | 5.36(3.68,7.27) | 0.47(0.33,0.64) |  | 8.09(5.56,11.04) | 0.37(0.25,0.51) |  | 51.01(34.99,69.76) | 0.466410817 |
| Libya | 8.98(6.17,12.04) | 0.50(0.34,0.66) |  | 7.40(5.15,10.08) | 0.50(0.35,0.68) |  | -17.57(-24.30,-9.80) | -0.141105041 |
| Lithuania | 6.64(4.94,8.59) | 0.80(0.60,1.03) |  | 3.24(2.41,4.19) | 0.79(0.59,1.03) |  | -51.23(-55.47,-47.12) | -0.252957698 |
| Luxembourg | 0.39(0.28,0.52) | 0.59(0.42,0.78) |  | 0.70(0.51,0.90) | 0.69(0.51,0.89) |  | 78.05(61.90,96.88) | 0.542569668 |
| Madagascar | 20.05(13.73,27.31) | 0.37(0.25,0.50) |  | 38.17(26.18,52.78) | 0.33(0.22,0.45) |  | 90.38(71.16,111.66) | -0.807546046 |
| Malawi | 15.75(10.72,21.47) | 0.35(0.24,0.47) |  | 22.96(15.39,32.25) | 0.28(0.19,0.40) |  | 45.78(29.72,64.72) | -1.106069541 |
| Malaysia | 31.47(22.15,41.65) | 0.48(0.34,0.63) |  | 37.71(26.62,50.51) | 0.50(0.35,0.66) |  | 19.84(9.34,30.87) | 0.194239685 |
| Maldives | 0.36(0.25,0.48) | 0.34(0.24,0.46) |  | 0.44(0.31,0.60) | 0.44(0.31,0.60) |  | 22.89(11.28,37.20) | 0.367714344 |
| Mali | 12.90(8.76,17.61) | 0.31(0.21,0.43) |  | 39.29(27.23,53.37) | 0.34(0.24,0.46) |  | 204.58(171.27,240.59) | 0.326471919 |
| Malta | 0.54(0.39,0.70) | 0.61(0.44,0.81) |  | 0.45(0.33,0.58) | 0.70(0.51,0.90) |  | -16.83(-24.31,-8.40) | 0.355923648 |
| Marshall Islands | 0.10(0.07,0.13) | 0.44(0.31,0.59) |  | 0.08(0.05,0.10) | 0.44(0.30,0.59) |  | -21.93(-28.29,-14.97) | 0.261485416 |
| Mauritania | 2.75(1.87,3.75) | 0.30(0.20,0.41) |  | 8.66(5.92,11.65) | 0.47(0.32,0.63) |  | 215.59(177.33,254.13) | 1.123015606 |
| Mauritius | 2.83(1.99,3.79) | 0.86(0.60,1.15) |  | 1.76(1.22,2.36) | 0.85(0.59,1.14) |  | -37.82(-43.19,-32.42) | 0.253827986 |
| Mexico | 190.49(134.96,251.91) | 0.57(0.40,0.75) |  | 228.28(165.48,295.40) | 0.71(0.52,0.92) |  | 19.84(15.24,24.64) | 0.327281968 |
| Micronesia (Federated States of) | 0.22(0.15,0.29) | 0.47(0.33,0.63) |  | 0.14(0.10,0.19) | 0.46(0.31,0.63) |  | -34.76(-40.10,-28.48) | 0.273777842 |
| Monaco | 0.02(0.02,0.03) | 0.70(0.51,0.91) |  | 0.04(0.03,0.05) | 0.76(0.56,0.98) |  | 52.28(40.08,65.64) | 0.358619147 |
| Mongolia | 4.22(3.05,5.60) | 0.47(0.34,0.62) |  | 5.95(4.22,7.84) | 0.55(0.39,0.72) |  | 40.99(29.13,52.79) | 0.054013121 |
| Montenegro | 1.28(0.95,1.66) | 0.79(0.59,1.02) |  | 0.88(0.64,1.12) | 0.79(0.58,1.00) |  | -31.51(-36.67,-25.98) | -0.008958716 |
| Morocco | 35.77(24.72,49.13) | 0.37(0.25,0.50) |  | 37.79(25.91,51.14) | 0.39(0.26,0.52) |  | 5.65(-3.33,14.93) | 0.204835841 |
| Mozambique | 20.79(14.18,29.28) | 0.34(0.23,0.47) |  | 41.60(27.90,58.28) | 0.29(0.20,0.41) |  | 100.14(82.48,123.45) | -0.619728791 |
| Myanmar | 41.73(27.93,57.01) | 0.28(0.19,0.39) |  | 44.80(30.19,62.02) | 0.29(0.19,0.40) |  | 7.37(-2.25,17.80) | 0.128116854 |
| Namibia | 3.45(2.40,4.74) | 0.57(0.40,0.79) |  | 4.78(3.30,6.43) | 0.58(0.40,0.78) |  | 38.38(24.65,53.65) | -0.203564905 |
| Nauru | 0.02(0.01,0.02) | 0.41(0.28,0.55) |  | 0.02(0.01,0.02) | 0.41(0.28,0.56) |  | -6.73(-14.97,1.55) | 0.304576155 |
| Nepal | 22.58(15.39,30.77) | 0.27(0.18,0.37) |  | 27.16(18.10,37.63) | 0.29(0.20,0.41) |  | 20.25(8.84,32.53) | 0.359550659 |
| Netherlands | 22.72(16.70,29.46) | 0.83(0.61,1.08) |  | 24.48(17.78,31.21) | 0.91(0.66,1.16) |  | 7.76(0.66,17.06) | 0.439057886 |
| New Zealand | 4.39(3.19,5.71) | 0.55(0.40,0.71) |  | 5.16(3.68,6.80) | 0.53(0.37,0.69) |  | 17.56(10.68,24.33) | -0.11910801 |
| Nicaragua | 8.34(5.96,11.08) | 0.46(0.33,0.61) |  | 11.70(8.30,15.52) | 0.59(0.42,0.78) |  | 40.38(28.62,52.97) | 0.254126251 |
| Niger | 10.90(7.30,14.88) | 0.27(0.18,0.37) |  | 40.78(27.18,56.00) | 0.32(0.21,0.44) |  | 274.28(235.35,315.32) | 0.906274931 |
| Nigeria | 152.70(104.91,207.08) | 0.39(0.27,0.53) |  | 600.49(415.80,811.19) | 0.59(0.41,0.80) |  | 293.24(272.11,313.97) | 1.74704044 |
| Niue | 0.00(0.00,0.01) | 0.52(0.37,0.69) |  | 0.00(0.00,0.00) | 0.51(0.36,0.69) |  | -53.63(-57.87,-49.56) | 0.172509594 |
| North Macedonia | 4.17(3.05,5.32) | 0.79(0.58,1.01) |  | 2.49(1.81,3.21) | 0.76(0.55,0.98) |  | -40.38(-45.96,-35.83) | -0.181370818 |
| Northern Mariana Islands | 0.07(0.05,0.10) | 0.59(0.43,0.79) |  | 0.07(0.05,0.09) | 0.63(0.46,0.83) |  | -1.73(-9.50,5.69) | 0.695372234 |
| Norway | 6.28(4.65,8.07) | 0.79(0.58,1.01) |  | 7.62(5.67,9.73) | 0.82(0.61,1.05) |  | 21.39(18.29,24.33) | 0.21162445 |
| Oman | 4.09(2.85,5.41) | 0.49(0.34,0.64) |  | 6.29(4.46,8.41) | 0.51(0.36,0.69) |  | 53.67(41.88,65.07) | 0.115148574 |
| Pakistan | 145.77(100.11,198.10) | 0.30(0.20,0.40) |  | 232.38(159.38,320.71) | 0.27(0.19,0.38) |  | 59.42(49.79,67.97) | 0.139276341 |
| Palau | 0.02(0.02,0.03) | 0.52(0.36,0.70) |  | 0.02(0.01,0.02) | 0.51(0.36,0.70) |  | -29.51(-35.12,-23.37) | 0.303662598 |
| Palestine | 4.16(3.02,5.43) | 0.43(0.31,0.56) |  | 7.77(5.42,10.44) | 0.42(0.29,0.56) |  | 86.59(65.52,107.05) | 0.530916666 |
| Panama | 4.94(3.53,6.63) | 0.59(0.42,0.79) |  | 8.23(5.91,10.64) | 0.71(0.51,0.92) |  | 66.50(53.25,81.10) | 0.363631065 |
| Papua New Guinea | 5.84(4.02,8.05) | 0.34(0.24,0.47) |  | 12.82(8.64,17.68) | 0.33(0.22,0.45) |  | 119.54(100.80,139.79) | 0.231910219 |
| Paraguay | 8.72(6.28,11.31) | 0.52(0.38,0.68) |  | 10.52(7.45,13.70) | 0.52(0.37,0.68) |  | 20.76(11.62,30.91) | -0.284260476 |
| Peru | 46.47(32.66,62.47) | 0.56(0.39,0.75) |  | 49.83(35.13,67.67) | 0.52(0.37,0.71) |  | 7.24(-1.64,16.65) | -0.328033394 |
| Philippines | 121.41(84.34,162.85) | 0.48(0.33,0.65) |  | 159.45(110.67,214.79) | 0.47(0.33,0.63) |  | 31.33(28.32,34.07) | -0.194483235 |
| Poland | 75.03(55.46,94.97) | 0.78(0.58,0.99) |  | 45.08(33.10,57.09) | 0.77(0.56,0.97) |  | -39.91(-41.61,-38.26) | -0.213828368 |
| Portugal | 8.43(6.01,11.33) | 0.40(0.28,0.54) |  | 5.45(3.90,7.28) | 0.40(0.29,0.53) |  | -35.32(-40.84,-29.71) | 0.054697274 |
| Puerto Rico | 6.79(4.97,8.74) | 0.68(0.50,0.88) |  | 3.20(2.32,4.11) | 0.72(0.52,0.93) |  | -52.88(-56.46,-48.93) | 0.131590283 |
| Qatar | 0.60(0.43,0.79) | 0.48(0.34,0.63) |  | 2.50(1.79,3.24) | 0.51(0.36,0.66) |  | 315.13(287.01,351.95) | 0.042702054 |
| Republic of Korea | 67.60(49.51,86.53) | 0.59(0.44,0.76) |  | 37.08(27.21,48.10) | 0.61(0.45,0.79) |  | -45.15(-48.95,-40.92) | 0.087481292 |
| Republic of Moldova | 9.55(7.07,12.21) | 0.77(0.57,0.99) |  | 4.31(3.18,5.58) | 0.83(0.61,1.07) |  | -54.89(-59.21,-50.34) | 0.108728304 |
| Romania | 37.88(27.61,48.42) | 0.68(0.50,0.87) |  | 18.72(13.29,24.17) | 0.62(0.44,0.80) |  | -50.58(-54.49,-45.26) | -0.372884605 |
| Russian Federation | 286.71(213.35,361.59) | 0.83(0.61,1.04) |  | 219.22(162.68,277.99) | 0.84(0.62,1.07) |  | -23.54(-25.10,-21.90) | -0.324835505 |
| Rwanda | 14.77(10.03,20.14) | 0.44(0.30,0.59) |  | 14.49(9.86,20.10) | 0.29(0.20,0.40) |  | -1.85(-14.29,10.66) | -1.518897932 |
| Saint Kitts and Nevis | 0.08(0.06,0.11) | 0.57(0.42,0.76) |  | 0.05(0.03,0.07) | 0.50(0.35,0.68) |  | -39.29(-44.41,-34.17) | -0.415035271 |
| Saint Lucia | 0.29(0.21,0.37) | 0.55(0.40,0.72) |  | 0.17(0.13,0.23) | 0.58(0.43,0.77) |  | -39.59(-44.62,-34.21) | 0.18589316 |
| Saint Vincent and the Grenadines | 0.21(0.15,0.28) | 0.52(0.37,0.69) |  | 0.14(0.10,0.19) | 0.55(0.40,0.74) |  | -35.82(-41.03,-31.01) | -0.012242388 |
| Samoa | 0.39(0.28,0.51) | 0.55(0.39,0.71) |  | 0.41(0.29,0.54) | 0.51(0.36,0.67) |  | 4.11(-4.79,13.40) | 0.116093124 |
| San Marino | 0.03(0.02,0.04) | 0.73(0.52,0.95) |  | 0.03(0.03,0.04) | 0.77(0.57,1.00) |  | 14.35(5.99,23.77) | 0.331285735 |
| Sao Tome and Principe | 0.21(0.15,0.28) | 0.37(0.26,0.50) |  | 0.37(0.26,0.51) | 0.48(0.34,0.66) |  | 79.09(61.43,95.09) | 1.136913528 |
| Saudi Arabia | 26.80(18.83,36.62) | 0.41(0.29,0.56) |  | 37.39(26.43,50.32) | 0.49(0.35,0.67) |  | 39.50(26.53,53.43) | 0.800546014 |
| Senegal | 18.12(13.00,24.61) | 0.50(0.36,0.67) |  | 26.17(18.04,35.87) | 0.41(0.28,0.56) |  | 44.38(29.06,60.96) | -1.040862955 |
| Serbia | 14.00(10.32,18.05) | 0.65(0.48,0.83) |  | 9.93(7.32,12.66) | 0.75(0.55,0.95) |  | -29.06(-34.41,-22.17) | 0.541000617 |
| Seychelles | 0.11(0.08,0.15) | 0.48(0.34,0.64) |  | 0.12(0.08,0.16) | 0.50(0.35,0.67) |  | 2.46(-6.59,12.39) | 0.152767227 |
| Sierra Leone | 5.17(3.49,7.04) | 0.28(0.19,0.39) |  | 12.32(8.23,16.98) | 0.34(0.23,0.47) |  | 138.47(113.52,165.95) | 0.956037653 |
| Singapore | 3.37(2.42,4.41) | 0.52(0.37,0.68) |  | 3.79(2.75,4.88) | 0.47(0.34,0.60) |  | 12.42(2.97,22.68) | -0.309503631 |
| Slovakia | 10.64(7.84,13.64) | 0.80(0.59,1.03) |  | 7.40(5.48,9.55) | 0.86(0.64,1.11) |  | -30.48(-36.73,-24.83) | -0.003792062 |
| Slovenia | 3.79(2.83,4.88) | 0.92(0.69,1.18) |  | 2.72(2.02,3.47) | 0.87(0.65,1.11) |  | -28.40(-34.73,-22.61) | -0.35927325 |
| Solomon Islands | 0.67(0.47,0.90) | 0.43(0.30,0.58) |  | 1.09(0.75,1.46) | 0.42(0.29,0.56) |  | 62.22(48.67,76.79) | 0.273116314 |
| Somalia | 12.63(8.56,17.33) | 0.32(0.22,0.44) |  | 27.48(19.03,37.50) | 0.27(0.18,0.36) |  | 117.53(98.51,139.65) | -0.747220324 |
| South Africa | 66.49(46.14,90.98) | 0.49(0.34,0.67) |  | 83.55(57.60,112.73) | 0.55(0.38,0.74) |  | 25.65(20.57,30.60) | 0.201206409 |
| South Sudan | 7.99(5.47,10.98) | 0.30(0.21,0.42) |  | 12.14(8.30,17.13) | 0.28(0.19,0.40) |  | 51.94(37.89,67.91) | -0.602093722 |
| Spain | 44.02(31.48,57.98) | 0.56(0.40,0.74) |  | 37.16(26.73,48.25) | 0.57(0.41,0.74) |  | -15.57(-22.14,-9.29) | -0.072728939 |
| Sri Lanka | 32.44(22.70,43.51) | 0.59(0.41,0.79) |  | 31.53(22.26,41.69) | 0.62(0.44,0.82) |  | -2.81(-11.15,5.55) | 0.204000421 |
| Sudan | 26.46(17.87,36.02) | 0.30(0.20,0.41) |  | 50.81(34.03,69.93) | 0.31(0.21,0.42) |  | 92.05(75.13,112.31) | 0.230050458 |
| Suriname | 0.77(0.54,1.02) | 0.59(0.41,0.79) |  | 0.80(0.56,1.09) | 0.56(0.39,0.76) |  | 5.03(-3.73,13.24) | -0.299450085 |
| Sweden | 19.58(14.41,24.84) | 1.27(0.93,1.61) |  | 25.69(18.91,32.75) | 1.41(1.04,1.80) |  | 31.21(22.88,40.22) | 0.175847643 |
| Switzerland | 15.73(11.43,20.45) | 1.36(0.99,1.77) |  | 19.28(14.16,25.06) | 1.45(1.06,1.88) |  | 22.57(13.54,31.35) | 0.331592642 |
| Syrian Arab Republic | 23.77(16.58,31.72) | 0.40(0.28,0.54) |  | 16.73(11.34,23.00) | 0.46(0.31,0.63) |  | -29.61(-36.02,-23.09) | 0.655743052 |
| Taiwan (Province of China) | 34.41(24.78,44.68) | 0.62(0.45,0.81) |  | 17.38(12.53,22.92) | 0.59(0.43,0.78) |  | -49.47(-53.95,-44.73) | -0.102275564 |
| Tajikistan | 12.25(8.80,16.16) | 0.53(0.38,0.70) |  | 19.48(13.82,25.74) | 0.54(0.39,0.72) |  | 59.09(46.10,71.58) | -0.042679773 |
| Thailand | 78.59(54.15,105.08) | 0.47(0.32,0.62) |  | 49.37(34.82,66.54) | 0.51(0.36,0.68) |  | -37.17(-41.99,-32.11) | 0.367509721 |
| Timor-Leste | 0.96(0.65,1.29) | 0.29(0.20,0.39) |  | 1.69(1.16,2.29) | 0.33(0.22,0.44) |  | 75.98(59.71,94.29) | 0.493472835 |
| Togo | 7.66(5.30,10.38) | 0.43(0.30,0.59) |  | 12.26(8.55,16.82) | 0.37(0.26,0.51) |  | 60.07(45.43,76.55) | -0.184989964 |
| Tokelau | 0.00(0.00,0.00) | 0.49(0.34,0.66) |  | 0.00(0.00,0.00) | 0.57(0.40,0.77) |  | -24.44(-30.35,-17.69) | 1.129665652 |
| Tonga | 0.21(0.15,0.29) | 0.51(0.36,0.69) |  | 0.19(0.13,0.26) | 0.49(0.35,0.66) |  | -10.43(-18.25,-1.96) | 0.184438329 |
| Trinidad and Tobago | 2.13(1.51,2.81) | 0.52(0.37,0.69) |  | 1.55(1.11,2.05) | 0.57(0.41,0.75) |  | -27.12(-32.58,-21.17) | -0.089524529 |
| Tunisia | 14.19(9.88,19.03) | 0.46(0.32,0.61) |  | 13.77(9.76,18.40) | 0.50(0.35,0.67) |  | -2.95(-11.05,4.84) | 0.191609995 |
| Turkey | 73.87(50.83,101.72) | 0.36(0.25,0.50) |  | 96.84(67.98,130.13) | 0.52(0.37,0.70) |  | 31.08(18.83,44.89) | 1.316804568 |
| Turkmenistan | 8.16(5.78,10.81) | 0.54(0.38,0.72) |  | 8.57(6.08,11.43) | 0.56(0.40,0.75) |  | 4.96(-3.90,13.53) | 0.143006271 |
| Tuvalu | 0.01(0.01,0.02) | 0.38(0.26,0.52) |  | 0.01(0.01,0.02) | 0.38(0.26,0.52) |  | 6.76(-2.62,17.46) | 0.380336107 |
| Uganda | 43.06(28.79,60.29) | 0.51(0.34,0.72) |  | 68.61(46.40,93.55) | 0.35(0.23,0.47) |  | 59.35(44.81,78.47) | -1.192678773 |
| Ukraine | 94.20(70.50,121.37) | 0.83(0.62,1.07) |  | 53.05(38.63,67.98) | 0.84(0.61,1.07) |  | -43.68(-48.93,-38.72) | -0.349139704 |
| United Arab Emirates | 2.64(1.86,3.45) | 0.45(0.32,0.59) |  | 6.63(4.67,8.75) | 0.50(0.35,0.65) |  | 151.56(129.46,173.17) | 0.338772067 |
| United Kingdom | 44.50(32.17,58.01) | 0.41(0.29,0.53) |  | 48.99(35.28,63.75) | 0.42(0.30,0.54) |  | 10.10(8.02,12.03) | 0.023900891 |
| United Republic of Tanzania | 47.25(32.33,64.52) | 0.39(0.27,0.53) |  | 76.58(53.04,104.41) | 0.31(0.22,0.43) |  | 62.06(45.47,81.40) | -0.588108189 |
| United States of America | 212.96(155.03,276.93) | 0.38(0.28,0.50) |  | 226.59(162.62,297.61) | 0.38(0.27,0.50) |  | 6.40(3.03,10.06) | -0.009333445 |
| United States Virgin Islands | 0.20(0.15,0.26) | 0.63(0.46,0.81) |  | 0.08(0.06,0.11) | 0.63(0.46,0.82) |  | -57.74(-61.14,-54.15) | -0.015531463 |
| Uruguay | 3.84(2.81,5.01) | 0.47(0.34,0.61) |  | 2.92(2.12,3.79) | 0.44(0.32,0.58) |  | -23.96(-30.55,-18.35) | -0.34272337 |
| Uzbekistan | 51.35(37.07,66.70) | 0.60(0.43,0.78) |  | 55.89(39.88,73.73) | 0.55(0.40,0.73) |  | 8.83(-0.20,19.43) | -0.060132552 |
| Vanuatu | 0.26(0.19,0.35) | 0.38(0.27,0.51) |  | 0.45(0.31,0.61) | 0.38(0.27,0.53) |  | 70.64(56.24,87.06) | 0.31991075 |
| Venezuela (Bolivarian Republic of) | 46.77(33.71,61.25) | 0.66(0.48,0.86) |  | 45.93(33.22,60.04) | 0.69(0.50,0.91) |  | -1.80(-8.48,6.47) | 0.105478175 |
| Viet Nam | 106.59(74.71,140.16) | 0.40(0.28,0.53) |  | 104.23(73.57,141.06) | 0.42(0.30,0.57) |  | -2.21(-10.41,6.57) | 0.113393115 |
| Yemen | 31.09(21.10,42.24) | 0.44(0.30,0.60) |  | 55.76(37.72,76.55) | 0.40(0.27,0.56) |  | 79.34(62.34,95.69) | -0.534530796 |
| Zambia | 14.68(9.98,20.31) | 0.39(0.27,0.54) |  | 24.15(16.05,33.91) | 0.29(0.19,0.41) |  | 64.49(45.86,84.12) | -1.176373547 |
| Zimbabwe | 18.73(12.61,25.85) | 0.39(0.26,0.54) |  | 25.45(17.70,35.57) | 0.40(0.28,0.57) |  | 35.87(22.46,51.37) | -0.486904888 |
