# Supplementary material for: Burden of pulmonary arterial hypertension in children globally, regionally, and nationally (1990–2021): results from the global burden of disease study
Source: Front Pediatr. 2025 Jun 30;13:1527281. doi: 10.3389/fped.2025.1527281 (PMC12256471; doi:10.3389/fped.2025.1527281)
Supplement: Supplementary file 7 [file Table4.docx]

Table S4 Death of Pediatric Pulmonary Arterial Hypertension at the national level

| location | 1990 | |  | 2021 | |  | 1990-2021 | |
| --- | --- | --- | --- | --- | --- | --- | --- | --- |
|  | death case | death rate |  | death case | death rate |  | Cases change | EAPC |
| Afghanistan | 19.15(8.93,32.80) | 0.44(0.21,0.76) |  | 59.29(29.19,90.18) | 0.42(0.21,0.64) |  | 209.57(37.02,505.48) | 0.47(0.18,0.76) |
| Albania | 1.47(0.54,2.02) | 0.13(0.05,0.18) |  | 0.20(0.12,0.31) | 0.05(0.03,0.07) |  | -86.35(-91.92,-64.78) | -2.75(-3.04,-2.46) |
| Algeria | 28.01(15.61,49.48) | 0.26(0.15,0.46) |  | 15.68(9.87,22.89) | 0.12(0.07,0.17) |  | -44.02(-76.78,6.08) | -1.03(-1.59,-0.46) |
| American Samoa | 0.01(0.00,0.01) | 0.03(0.02,0.06) |  | 0.00(0.00,0.01) | 0.02(0.01,0.05) |  | -56.02(-73.95,-32.75) | -1.60(-1.77,-1.42) |
| Andorra | 0.01(0.01,0.02) | 0.12(0.06,0.20) |  | 0.00(0.00,0.00) | 0.02(0.01,0.02) |  | -86.09(-92.72,-68.95) | -6.28(-6.62,-5.94) |
| Angola | 8.66(2.93,23.69) | 0.18(0.06,0.50) |  | 8.63(5.21,16.40) | 0.06(0.03,0.11) |  | -0.29(-45.03,140.36) | -3.56(-3.99,-3.14) |
| Antigua and Barbuda | 0.01(0.01,0.01) | 0.05(0.04,0.07) |  | 0.00(0.00,0.00) | 0.01(0.01,0.01) |  | -83.05(-87.22,-77.98) | -6.56(-7.21,-5.91) |
| Argentina | 34.00(29.51,39.51) | 0.34(0.29,0.39) |  | 4.39(3.59,5.24) | 0.04(0.04,0.05) |  | -87.10(-90.07,-83.56) | -6.11(-6.41,-5.81) |
| Armenia | 0.27(0.22,0.35) | 0.03(0.02,0.03) |  | 0.01(0.01,0.01) | 0.00(0.00,0.00) |  | -95.64(-96.78,-94.17) | -7.56(-8.53,-6.58) |
| Australia | 3.44(2.67,4.34) | 0.09(0.07,0.11) |  | 1.09(0.88,1.36) | 0.02(0.02,0.03) |  | -68.26(-76.97,-56.73) | -3.93(-4.56,-3.31) |
| Austria | 0.56(0.49,0.64) | 0.04(0.04,0.05) |  | 0.19(0.17,0.22) | 0.01(0.01,0.02) |  | -65.72(-72.24,-58.35) | -2.52(-3.18,-1.86) |
| Azerbaijan | 2.37(1.08,4.16) | 0.10(0.04,0.17) |  | 0.83(0.59,1.14) | 0.04(0.02,0.05) |  | -65.14(-82.22,-7.75) | -3.11(-3.27,-2.95) |
| Bahamas | 0.35(0.29,0.43) | 0.44(0.36,0.53) |  | 0.05(0.04,0.07) | 0.06(0.05,0.08) |  | -85.17(-89.23,-79.77) | -7.04(-7.64,-6.43) |
| Bahrain | 0.21(0.14,0.37) | 0.13(0.09,0.23) |  | 0.14(0.07,0.23) | 0.05(0.02,0.08) |  | -34.43(-71.72,13.52) | -1.69(-2.80,-0.58) |
| Bangladesh | 87.72(30.19,158.28) | 0.18(0.06,0.32) |  | 30.79(17.49,46.94) | 0.07(0.04,0.10) |  | -64.90(-81.31,-25.59) | -2.71(-2.90,-2.52) |
| Barbados | 0.21(0.17,0.25) | 0.33(0.27,0.40) |  | 0.03(0.02,0.05) | 0.07(0.05,0.10) |  | -83.77(-88.44,-78.03) | -5.43(-5.91,-4.96) |
| Belarus | 0.90(0.57,1.39) | 0.04(0.02,0.06) |  | 0.19(0.15,0.24) | 0.01(0.01,0.01) |  | -79.11(-87.76,-65.20) | -2.94(-3.32,-2.57) |
| Belgium | 1.53(1.09,2.09) | 0.08(0.06,0.12) |  | 0.56(0.47,0.67) | 0.03(0.02,0.04) |  | -63.01(-73.90,-46.93) | -3.18(-3.54,-2.83) |
| Belize | 0.41(0.32,0.49) | 0.50(0.39,0.60) |  | 0.05(0.04,0.06) | 0.04(0.03,0.05) |  | -87.00(-90.21,-82.65) | -8.43(-8.96,-7.90) |
| Benin | 3.57(1.49,8.17) | 0.15(0.06,0.34) |  | 5.64(3.45,8.82) | 0.09(0.06,0.15) |  | 57.93(-4.69,217.14) | -1.00(-1.21,-0.80) |
| Bermuda | 0.05(0.04,0.06) | 0.40(0.33,0.50) |  | 0.00(0.00,0.01) | 0.05(0.03,0.07) |  | -90.80(-93.63,-86.79) | -6.82(-7.16,-6.47) |
| Bhutan | 0.76(0.28,1.18) | 0.29(0.11,0.45) |  | 0.19(0.12,0.30) | 0.10(0.06,0.16) |  | -74.93(-86.31,-36.47) | -3.51(-3.77,-3.26) |
| Bolivia (Plurinational State of) | 14.60(5.83,25.75) | 0.54(0.22,0.96) |  | 5.87(3.78,8.68) | 0.17(0.11,0.25) |  | -59.78(-75.39,-14.32) | -3.55(-3.64,-3.47) |
| Bosnia and Herzegovina | 0.21(0.11,0.53) | 0.02(0.01,0.05) |  | 0.05(0.03,0.12) | 0.01(0.01,0.02) |  | -76.35(-86.75,-54.35) | -2.04(-2.31,-1.76) |
| Botswana | 0.25(0.16,0.37) | 0.04(0.03,0.06) |  | 0.29(0.18,0.45) | 0.04(0.03,0.07) |  | 15.02(-34.39,93.58) | 0.74(0.32,1.16) |
| Brazil | 93.49(81.26,107.41) | 0.18(0.16,0.21) |  | 38.08(30.89,46.39) | 0.08(0.06,0.10) |  | -59.27(-68.87,-49.00) | -2.10(-2.87,-1.33) |
| Brunei Darussalam | 0.33(0.18,0.45) | 0.36(0.19,0.50) |  | 0.16(0.10,0.23) | 0.17(0.10,0.24) |  | -51.01(-67.64,-22.87) | -2.28(-2.47,-2.10) |
| Bulgaria | 0.38(0.32,0.45) | 0.02(0.02,0.03) |  | 0.06(0.05,0.08) | 0.01(0.01,0.01) |  | -83.12(-87.40,-77.27) | -2.82(-3.40,-2.24) |
| Burkina Faso | 8.73(3.35,17.89) | 0.18(0.07,0.38) |  | 14.23(7.94,22.99) | 0.14(0.08,0.22) |  | 63.03(0.53,196.56) | -0.55(-0.74,-0.37) |
| Burundi | 7.29(3.34,17.14) | 0.28(0.13,0.65) |  | 5.09(2.65,9.21) | 0.09(0.05,0.16) |  | -30.15(-65.78,38.07) | -3.07(-3.50,-2.64) |
| Cabo Verde | 0.22(0.11,0.42) | 0.14(0.07,0.27) |  | 0.05(0.02,0.11) | 0.04(0.01,0.08) |  | -76.55(-88.45,-52.41) | -4.51(-4.70,-4.33) |
| Cambodia | 4.90(1.96,13.66) | 0.11(0.04,0.29) |  | 2.63(1.57,4.87) | 0.05(0.03,0.10) |  | -46.31(-71.97,16.70) | -2.24(-2.29,-2.19) |
| Cameroon | 5.54(2.58,12.47) | 0.11(0.05,0.26) |  | 10.86(6.98,18.00) | 0.08(0.05,0.13) |  | 96.00(25.08,242.38) | -0.34(-0.84,0.16) |
| Canada | 9.44(8.50,10.33) | 0.16(0.15,0.18) |  | 2.51(2.12,3.01) | 0.04(0.03,0.05) |  | -73.41(-77.70,-67.86) | -5.04(-5.54,-4.53) |
| Central African Republic | 2.34(0.90,5.88) | 0.19(0.07,0.48) |  | 2.41(1.35,4.53) | 0.11(0.06,0.20) |  | 2.76(-38.05,107.75) | -1.71(-1.83,-1.58) |
| Chad | 5.21(1.59,12.07) | 0.18(0.05,0.41) |  | 14.23(6.15,27.59) | 0.16(0.07,0.31) |  | 172.88(80.67,389.18) | -0.18(-0.35,-0.02) |
| Chile | 5.36(4.77,5.97) | 0.13(0.12,0.15) |  | 1.31(1.10,1.54) | 0.04(0.03,0.04) |  | -75.51(-80.50,-70.12) | -3.79(-4.19,-3.40) |
| China | 636.24(426.25,943.30) | 0.20(0.13,0.30) |  | 97.33(59.31,149.60) | 0.04(0.02,0.06) |  | -84.70(-92.94,-72.82) | -3.69(-4.36,-3.01) |
| Colombia | 10.47(8.45,13.56) | 0.09(0.07,0.12) |  | 2.97(2.18,4.06) | 0.03(0.02,0.04) |  | -71.60(-81.40,-58.86) | -3.18(-3.68,-2.68) |
| Comoros | 0.48(0.21,0.95) | 0.22(0.10,0.45) |  | 0.18(0.09,0.38) | 0.08(0.04,0.16) |  | -62.10(-78.28,-29.63) | -3.65(-3.85,-3.45) |
| Congo | 1.05(0.48,2.61) | 0.10(0.05,0.25) |  | 0.62(0.42,1.15) | 0.03(0.02,0.06) |  | -41.05(-63.55,11.40) | -3.66(-4.13,-3.18) |
| Cook Islands | 0.01(0.01,0.02) | 0.13(0.08,0.23) |  | 0.00(0.00,0.01) | 0.07(0.02,0.16) |  | -70.01(-90.77,-29.22) | -4.29(-5.02,-3.55) |
| Costa Rica | 1.29(1.15,1.43) | 0.11(0.10,0.13) |  | 0.20(0.16,0.24) | 0.02(0.02,0.02) |  | -84.80(-88.01,-80.98) | -4.77(-5.54,-3.99) |
| Croatia | 8.36(3.91,18.31) | 0.15(0.07,0.32) |  | 11.81(7.74,19.46) | 0.10(0.07,0.17) |  | -88.39(-90.45,-85.93) | -0.58(-0.83,-0.33) |
| Cuba | 0.10(0.09,0.11) | 0.01(0.01,0.01) |  | 0.01(0.01,0.01) | 0.00(0.00,0.00) |  | -88.33(-91.06,-84.95) | -4.09(-5.14,-3.02) |
| Cyprus | 1.68(1.40,1.99) | 0.07(0.06,0.08) |  | 0.20(0.16,0.24) | 0.01(0.01,0.01) |  | -83.05(-89.68,-67.35) | -6.04(-6.59,-5.48) |
| Czechia | 0.99(0.61,1.43) | 0.50(0.31,0.72) |  | 0.17(0.11,0.23) | 0.08(0.05,0.11) |  | -68.82(-78.61,-57.67) | -5.57(-5.92,-5.22) |
| C么te d'Ivoire | 0.93(0.75,1.16) | 0.04(0.03,0.05) |  | 0.29(0.23,0.35) | 0.02(0.01,0.02) |  | 41.17(-8.81,164.40) | -2.97(-3.26,-2.67) |
| Democratic People's Republic of Korea | 5.15(3.20,9.83) | 0.09(0.05,0.17) |  | 1.70(0.76,3.31) | 0.04(0.02,0.07) |  | -67.03(-85.78,-38.24) | -2.53(-2.82,-2.24) |
| Democratic Republic of the Congo | 28.25(11.76,59.09) | 0.16(0.07,0.33) |  | 17.65(10.25,29.21) | 0.05(0.03,0.08) |  | -37.50(-69.08,31.06) | -3.44(-3.80,-3.07) |
| Denmark | 1.17(0.99,1.33) | 0.13(0.11,0.15) |  | 0.34(0.29,0.40) | 0.04(0.03,0.04) |  | -70.96(-76.57,-63.78) | -4.20(-4.58,-3.83) |
| Djibouti | 0.29(0.11,0.70) | 0.16(0.06,0.40) |  | 0.28(0.13,0.58) | 0.07(0.03,0.14) |  | -3.75(-49.42,99.39) | -2.88(-3.25,-2.50) |
| Dominica | 0.01(0.01,0.02) | 0.06(0.03,0.08) |  | 0.00(0.00,0.01) | 0.03(0.02,0.04) |  | -72.83(-84.66,-45.59) | -2.86(-3.29,-2.43) |
| Dominican Republic | 7.19(3.13,10.67) | 0.27(0.12,0.40) |  | 2.24(1.36,3.58) | 0.08(0.05,0.12) |  | -68.88(-83.33,-17.89) | -4.59(-4.88,-4.31) |
| Ecuador | 6.65(5.14,8.92) | 0.17(0.13,0.23) |  | 3.99(3.05,5.19) | 0.08(0.06,0.10) |  | -39.94(-60.25,-14.75) | -1.93(-2.57,-1.28) |
| Egypt | 596.37(201.54,907.45) | 2.69(0.91,4.09) |  | 131.70(97.49,179.73) | 0.36(0.26,0.49) |  | -77.92(-86.44,-40.99) | -5.62(-6.02,-5.22) |
| El Salvador | 4.95(2.72,7.25) | 0.23(0.13,0.34) |  | 0.69(0.42,1.30) | 0.04(0.02,0.07) |  | -86.15(-93.58,-55.06) | -6.17(-6.49,-5.84) |
| Equatorial Guinea | 0.28(0.11,0.66) | 0.14(0.05,0.33) |  | 0.19(0.10,0.34) | 0.03(0.02,0.06) |  | -32.96(-63.96,84.47) | -5.35(-5.66,-5.04) |
| Eritrea | 3.14(1.28,8.02) | 0.20(0.08,0.50) |  | 2.22(1.11,4.74) | 0.09(0.04,0.19) |  | -29.35(-59.82,34.56) | -2.51(-2.66,-2.37) |
| Estonia | 0.05(0.04,0.05) | 0.01(0.01,0.01) |  | 0.01(0.00,0.01) | 0.00(0.00,0.00) |  | -87.76(-90.26,-84.96) | -3.20(-5.18,-1.18) |
| Eswatini | 0.22(0.14,0.34) | 0.06(0.04,0.09) |  | 0.17(0.12,0.24) | 0.04(0.03,0.06) |  | -23.65(-51.15,27.69) | -0.53(-0.75,-0.31) |
| Ethiopia | 39.14(16.09,96.87) | 0.16(0.07,0.40) |  | 26.71(14.34,57.11) | 0.06(0.03,0.13) |  | -31.74(-59.83,33.73) | -3.46(-3.63,-3.28) |
| Fiji | 0.16(0.10,0.33) | 0.06(0.04,0.12) |  | 0.14(0.08,0.29) | 0.05(0.03,0.11) |  | -15.88(-48.55,36.24) | -0.37(-0.59,-0.14) |
| Finland | 0.30(0.25,0.35) | 0.03(0.03,0.04) |  | 0.12(0.10,0.13) | 0.01(0.01,0.02) |  | -60.96(-69.32,-52.15) | -2.50(-3.33,-1.65) |
| France | 8.59(6.61,11.36) | 0.07(0.06,0.10) |  | 4.62(3.98,5.35) | 0.04(0.03,0.05) |  | -46.15(-62.01,-25.19) | -2.05(-2.42,-1.67) |
| Gabon | 0.28(0.15,0.59) | 0.07(0.04,0.15) |  | 0.17(0.10,0.28) | 0.03(0.02,0.04) |  | -41.83(-67.92,17.31) | -2.51(-2.88,-2.13) |
| Gambia | 0.55(0.25,1.27) | 0.12(0.05,0.27) |  | 0.66(0.41,1.21) | 0.07(0.04,0.12) |  | 20.90(-23.94,118.02) | -1.87(-2.22,-1.51) |
| Georgia | 0.49(0.37,0.66) | 0.04(0.03,0.05) |  | 0.12(0.10,0.15) | 0.02(0.01,0.02) |  | -75.23(-82.67,-64.85) | -0.95(-3.12,1.27) |
| Germany | 14.28(12.01,16.86) | 0.11(0.09,0.13) |  | 4.92(4.16,5.64) | 0.04(0.03,0.05) |  | -65.53(-72.60,-57.02) | -2.44(-3.10,-1.77) |
| Ghana | 5.72(2.56,14.52) | 0.09(0.04,0.22) |  | 4.19(2.23,8.08) | 0.03(0.02,0.06) |  | -26.73(-51.42,23.32) | -2.75(-2.93,-2.58) |
| Greece | 1.56(1.41,1.70) | 0.08(0.07,0.08) |  | 0.70(0.58,0.82) | 0.05(0.04,0.06) |  | -55.20(-63.03,-46.58) | -1.23(-1.78,-0.69) |
| Greenland | 0.11(0.03,0.18) | 0.81(0.21,1.29) |  | 0.01(0.01,0.02) | 0.11(0.06,0.17) |  | -88.23(-95.27,-44.55) | -6.40(-6.85,-5.95) |
| Grenada | 0.13(0.10,0.16) | 0.38(0.30,0.47) |  | 0.01(0.01,0.02) | 0.06(0.05,0.07) |  | -89.51(-91.99,-86.16) | -6.21(-6.72,-5.70) |
| Guam | 0.02(0.01,0.04) | 0.04(0.03,0.09) |  | 0.01(0.01,0.03) | 0.03(0.02,0.08) |  | -37.81(-61.64,-8.17) | 0.01(-0.40,0.41) |
| Guatemala | 6.88(5.76,8.74) | 0.17(0.14,0.22) |  | 1.44(1.14,1.84) | 0.03(0.02,0.04) |  | -79.04(-85.30,-70.99) | -6.35(-6.83,-5.87) |
| Guinea | 6.28(2.08,15.20) | 0.23(0.08,0.55) |  | 7.71(4.49,12.53) | 0.13(0.07,0.21) |  | 22.78(-28.85,174.01) | -1.24(-1.50,-0.97) |
| Guinea-Bissau | 0.90(0.31,2.16) | 0.19(0.06,0.45) |  | 0.81(0.45,1.38) | 0.09(0.05,0.15) |  | -10.79(-49.46,97.14) | -1.87(-2.33,-1.42) |
| Guyana | 0.62(0.50,0.74) | 0.21(0.17,0.25) |  | 0.32(0.24,0.43) | 0.15(0.11,0.20) |  | -47.89(-62.99,-27.25) | -2.88(-4.26,-1.49) |
| Haiti | 35.39(9.15,69.95) | 1.30(0.34,2.58) |  | 26.64(8.50,50.84) | 0.61(0.20,1.17) |  | -24.73(-52.26,40.17) | -2.15(-2.33,-1.96) |
| Honduras | 2.14(1.23,3.16) | 0.10(0.06,0.14) |  | 1.03(0.54,1.97) | 0.03(0.02,0.06) |  | -51.97(-73.39,28.57) | -3.62(-3.75,-3.48) |
| Hungary | 0.55(0.50,0.61) | 0.03(0.02,0.03) |  | 0.07(0.06,0.08) | 0.00(0.00,0.01) |  | -87.54(-89.81,-84.74) | -3.68(-4.65,-2.71) |
| Iceland | 0.05(0.04,0.06) | 0.08(0.07,0.09) |  | 0.02(0.02,0.03) | 0.04(0.03,0.04) |  | -52.92(-62.99,-41.75) | -3.49(-4.25,-2.73) |
| India | 699.67(279.94,1028.37) | 0.21(0.09,0.31) |  | 294.85(178.06,471.81) | 0.08(0.05,0.13) |  | -57.86(-70.00,-32.43) | -2.87(-3.05,-2.68) |
| Indonesia | 54.63(22.11,168.47) | 0.08(0.03,0.25) |  | 33.81(19.28,72.63) | 0.05(0.03,0.11) |  | -38.12(-62.29,14.09) | -1.58(-1.72,-1.45) |
| Iran (Islamic Republic of) | 212.81(120.13,360.05) | 0.84(0.47,1.42) |  | 16.75(11.81,30.46) | 0.08(0.06,0.15) |  | -92.13(-95.37,-75.65) | -5.14(-5.96,-4.31) |
| Iraq | 20.50(8.42,31.12) | 0.25(0.10,0.38) |  | 8.60(4.43,13.41) | 0.06(0.03,0.10) |  | -58.04(-77.06,-11.66) | -4.04(-4.28,-3.79) |
| Ireland | 0.40(0.36,0.44) | 0.04(0.04,0.04) |  | 0.17(0.14,0.19) | 0.02(0.01,0.02) |  | -57.81(-65.15,-48.79) | -2.66(-3.65,-1.66) |
| Israel | 3.46(2.75,4.58) | 0.23(0.18,0.30) |  | 1.31(1.11,1.53) | 0.05(0.04,0.06) |  | -62.00(-73.17,-50.73) | -3.98(-4.51,-3.44) |
| Italy | 5.44(5.12,5.80) | 0.06(0.06,0.06) |  | 1.05(0.91,1.18) | 0.01(0.01,0.02) |  | -80.71(-83.62,-77.72) | -5.28(-5.89,-4.67) |
| Jamaica | 1.43(1.17,1.73) | 0.17(0.14,0.21) |  | 0.12(0.09,0.16) | 0.02(0.02,0.03) |  | -91.54(-94.16,-87.84) | -7.40(-7.79,-7.00) |
| Japan | 49.51(47.76,51.30) | 0.21(0.21,0.22) |  | 20.16(18.09,22.06) | 0.13(0.12,0.14) |  | -59.28(-63.43,-54.92) | -2.33(-2.92,-1.73) |
| Jordan | 1.05(0.55,3.78) | 0.06(0.03,0.23) |  | 1.55(0.68,3.96) | 0.04(0.02,0.11) |  | 47.05(-50.44,210.34) | -0.07(-0.70,0.56) |
| Kazakhstan | 0.19(0.12,0.27) | 0.00(0.00,0.01) |  | 0.07(0.05,0.09) | 0.00(0.00,0.00) |  | -61.62(-76.05,-39.42) | -4.10(-4.59,-3.60) |
| Kenya | 7.41(3.86,16.75) | 0.07(0.03,0.15) |  | 5.61(3.01,11.07) | 0.03(0.02,0.06) |  | -24.28(-53.79,33.18) | -1.93(-2.18,-1.69) |
| Kiribati | 0.03(0.02,0.08) | 0.11(0.05,0.26) |  | 0.03(0.01,0.06) | 0.06(0.03,0.13) |  | -18.48(-48.07,33.29) | -1.61(-1.72,-1.49) |
| Kuwait | 0.58(0.50,0.67) | 0.10(0.09,0.12) |  | 1.17(0.93,1.45) | 0.14(0.11,0.17) |  | 102.77(55.18,162.92) | 3.03(1.18,4.91) |
| Kyrgyzstan | 0.21(0.16,0.26) | 0.01(0.01,0.02) |  | 0.04(0.03,0.05) | 0.00(0.00,0.00) |  | -80.81(-86.05,-73.00) | -6.42(-6.98,-5.85) |
| Lao People's Democratic Republic | 3.54(1.03,11.26) | 0.19(0.06,0.61) |  | 2.27(1.19,4.92) | 0.10(0.05,0.21) |  | -35.93(-64.73,58.76) | -2.26(-2.36,-2.16) |
| Latvia | 0.07(0.06,0.08) | 0.01(0.01,0.01) |  | 0.02(0.02,0.03) | 0.01(0.01,0.01) |  | -68.68(-75.59,-60.25) | -0.70(-2.78,1.44) |
| Lebanon | 5.04(2.32,8.85) | 0.48(0.22,0.85) |  | 1.66(1.09,2.39) | 0.13(0.09,0.19) |  | -67.16(-85.55,-17.26) | -3.90(-4.24,-3.56) |
| Lesotho | 0.25(0.15,0.41) | 0.04(0.02,0.06) |  | 0.25(0.15,0.36) | 0.04(0.02,0.06) |  | -1.01(-41.19,63.71) | 0.96(0.63,1.29) |
| Liberia | 2.96(1.14,7.35) | 0.26(0.10,0.65) |  | 1.97(1.12,3.35) | 0.09(0.05,0.15) |  | -33.36(-68.99,71.59) | -2.87(-3.17,-2.58) |
| Libya | 11.51(6.28,21.66) | 0.64(0.35,1.20) |  | 6.63(2.69,11.89) | 0.44(0.18,0.80) |  | -42.36(-74.17,5.85) | 0.13(-0.29,0.56) |
| Lithuania | 0.19(0.17,0.21) | 0.02(0.02,0.03) |  | 0.04(0.03,0.04) | 0.01(0.01,0.01) |  | -80.33(-83.37,-76.62) | -1.60(-3.32,0.14) |
| Luxembourg | 0.06(0.05,0.06) | 0.08(0.07,0.09) |  | 0.03(0.02,0.03) | 0.02(0.02,0.03) |  | -54.65(-64.96,-42.75) | -4.38(-4.77,-3.98) |
| Madagascar | 16.87(7.20,43.61) | 0.31(0.13,0.80) |  | 16.52(7.84,33.73) | 0.14(0.07,0.29) |  | -2.06(-38.78,79.63) | -2.17(-2.38,-1.97) |
| Malawi | 9.64(3.93,22.26) | 0.21(0.09,0.49) |  | 4.95(2.79,8.57) | 0.06(0.03,0.11) |  | -48.65(-72.11,9.32) | -3.89(-4.12,-3.67) |
| Malaysia | 2.08(1.33,4.81) | 0.03(0.02,0.07) |  | 1.15(0.71,3.23) | 0.02(0.01,0.04) |  | -45.03(-69.42,-6.43) | -1.54(-2.31,-0.76) |
| Maldives | 0.43(0.18,0.63) | 0.40(0.17,0.60) |  | 0.09(0.06,0.12) | 0.09(0.06,0.12) |  | -79.35(-87.98,-50.12) | -4.00(-4.40,-3.59) |
| Mali | 6.44(2.12,15.20) | 0.16(0.05,0.37) |  | 10.87(5.82,19.67) | 0.09(0.05,0.17) |  | 68.67(3.77,231.31) | -1.35(-1.55,-1.15) |
| Malta | 0.03(0.03,0.03) | 0.03(0.03,0.04) |  | 0.02(0.01,0.02) | 0.03(0.02,0.04) |  | -35.91(-49.47,-18.51) | -0.42(-0.94,0.11) |
| Marshall Islands | 0.01(0.01,0.02) | 0.06(0.04,0.11) |  | 0.01(0.01,0.02) | 0.05(0.03,0.11) |  | -32.00(-59.20,10.30) | -0.57(-1.03,-0.12) |
| Mauritania | 0.83(0.32,1.99) | 0.09(0.03,0.21) |  | 0.86(0.52,1.48) | 0.05(0.03,0.08) |  | 4.02(-37.19,111.98) | -1.96(-2.41,-1.50) |
| Mauritius | 0.37(0.33,0.42) | 0.11(0.10,0.13) |  | 0.40(0.33,0.46) | 0.19(0.16,0.22) |  | 6.85(-12.20,30.86) | 8.41(5.76,11.13) |
| Mexico | 36.71(27.99,46.65) | 0.11(0.08,0.14) |  | 9.79(7.75,12.31) | 0.03(0.02,0.04) |  | -73.35(-81.08,-61.82) | -4.09(-4.80,-3.39) |
| Micronesia (Federated States of) | 0.04(0.02,0.07) | 0.08(0.05,0.15) |  | 0.01(0.01,0.03) | 0.04(0.02,0.08) |  | -66.41(-81.27,-42.27) | -2.15(-2.22,-2.08) |
| Monaco | 0.00(0.00,0.00) | 0.04(0.02,0.08) |  | 0.00(0.00,0.00) | 0.03(0.01,0.04) |  | -5.80(-69.07,99.91) | -2.87(-3.42,-2.31) |
| Mongolia | 1.37(0.66,2.54) | 0.15(0.07,0.28) |  | 0.55(0.38,0.79) | 0.05(0.03,0.07) |  | -59.46(-80.93,-9.97) | -2.61(-3.17,-2.05) |
| Montenegro | 0.01(0.00,0.09) | 0.01(0.00,0.06) |  | 0.00(0.00,0.02) | 0.00(0.00,0.02) |  | -78.88(-93.69,-62.73) | -4.03(-4.50,-3.55) |
| Morocco | 40.53(21.10,74.15) | 0.41(0.22,0.76) |  | 15.38(9.07,24.60) | 0.16(0.09,0.25) |  | -62.05(-84.09,-31.94) | -2.19(-2.82,-1.56) |
| Mozambique | 11.71(4.63,24.45) | 0.19(0.07,0.39) |  | 9.54(5.40,15.59) | 0.07(0.04,0.11) |  | -18.54(-55.11,76.98) | -3.10(-3.32,-2.89) |
| Myanmar | 25.18(8.44,76.05) | 0.17(0.06,0.51) |  | 15.13(8.69,31.28) | 0.10(0.06,0.20) |  | -39.92(-64.35,40.70) | -1.90(-2.13,-1.68) |
| Namibia | 0.25(0.16,0.38) | 0.04(0.03,0.06) |  | 0.27(0.18,0.42) | 0.03(0.02,0.05) |  | 10.05(-35.43,90.35) | 0.04(-0.40,0.48) |
| Nauru | 0.00(0.00,0.01) | 0.10(0.06,0.20) |  | 0.00(0.00,0.01) | 0.09(0.05,0.19) |  | -16.38(-47.16,29.94) | -0.40(-1.00,0.19) |
| Nepal | 28.64(11.11,44.44) | 0.34(0.13,0.53) |  | 8.96(4.98,15.59) | 0.10(0.05,0.17) |  | -68.69(-84.95,-32.79) | -4.02(-4.18,-3.86) |
| Netherlands | 1.53(1.41,1.67) | 0.06(0.05,0.06) |  | 0.68(0.60,0.77) | 0.03(0.02,0.03) |  | -55.16(-61.14,-48.16) | -2.90(-3.63,-2.17) |
| New Zealand | 0.22(0.19,0.25) | 0.03(0.02,0.03) |  | 0.11(0.09,0.13) | 0.01(0.01,0.01) |  | -49.22(-59.46,-38.03) | -3.21(-4.37,-2.04) |
| Nicaragua | 2.42(1.02,3.56) | 0.13(0.06,0.20) |  | 0.33(0.17,0.99) | 0.02(0.01,0.05) |  | -86.47(-94.16,-44.50) | -6.78(-6.94,-6.61) |
| Niger | 7.98(2.83,17.91) | 0.20(0.07,0.44) |  | 13.37(6.42,24.24) | 0.10(0.05,0.19) |  | 67.50(-16.34,248.86) | -2.15(-2.40,-1.91) |
| Nigeria | 71.41(26.61,186.30) | 0.18(0.07,0.48) |  | 120.45(71.77,205.43) | 0.12(0.07,0.20) |  | 68.69(6.96,248.49) | -1.05(-1.39,-0.71) |
| Niue | 0.00(0.00,0.00) | 0.07(0.05,0.14) |  | 0.00(0.00,0.00) | 0.22(0.13,0.45) |  | 49.16(-5.58,125.29) | 0.95(-0.03,1.94) |
| North Macedonia | 0.16(0.08,0.32) | 0.03(0.01,0.06) |  | 0.02(0.01,0.07) | 0.00(0.00,0.02) |  | -89.76(-95.80,-73.41) | -4.58(-5.18,-3.96) |
| Northern Mariana Islands | 0.00(0.00,0.01) | 0.02(0.01,0.05) |  | 0.00(0.00,0.00) | 0.01(0.01,0.03) |  | -48.73(-68.70,-18.33) | -1.80(-2.23,-1.36) |
| Norway | 1.27(1.19,1.36) | 0.16(0.15,0.17) |  | 0.13(0.12,0.15) | 0.01(0.01,0.02) |  | -89.43(-90.77,-88.01) | -8.38(-9.33,-7.42) |
| Oman | 0.68(0.33,1.83) | 0.08(0.04,0.22) |  | 0.55(0.17,0.92) | 0.05(0.01,0.08) |  | -18.36(-81.05,92.87) | 0.77(-1.08,2.65) |
| Pakistan | 170.00(66.78,262.40) | 0.35(0.14,0.53) |  | 173.98(84.05,279.17) | 0.20(0.10,0.33) |  | 2.34(-32.34,49.16) | -0.71(-1.17,-0.24) |
| Palau | 0.00(0.00,0.01) | 0.06(0.03,0.12) |  | 0.00(0.00,0.00) | 0.03(0.02,0.07) |  | -60.42(-77.21,-31.85) | -1.70(-1.91,-1.49) |
| Palestine | 2.77(1.11,4.59) | 0.29(0.11,0.47) |  | 1.46(0.57,3.04) | 0.08(0.03,0.16) |  | -47.37(-73.64,8.33) | -3.33(-3.68,-2.98) |
| Panama | 0.60(0.50,0.73) | 0.07(0.06,0.09) |  | 0.17(0.13,0.21) | 0.01(0.01,0.02) |  | -72.43(-79.66,-62.70) | -5.88(-6.61,-5.15) |
| Papua New Guinea | 3.76(2.00,6.55) | 0.22(0.12,0.39) |  | 7.94(4.48,13.91) | 0.20(0.11,0.35) |  | 111.09(36.45,226.98) | -0.12(-0.29,0.04) |
| Paraguay | 0.70(0.47,1.39) | 0.04(0.03,0.08) |  | 0.40(0.21,1.09) | 0.02(0.01,0.05) |  | -43.48(-69.36,3.63) | -2.20(-2.49,-1.91) |
| Peru | 20.18(9.65,33.28) | 0.24(0.12,0.40) |  | 6.90(4.47,9.98) | 0.07(0.05,0.10) |  | -65.78(-83.96,-16.95) | -3.20(-3.41,-3.00) |
| Philippines | 20.48(12.33,37.02) | 0.08(0.05,0.15) |  | 10.33(7.33,26.34) | 0.03(0.02,0.08) |  | -49.54(-66.28,-21.61) | -2.82(-3.03,-2.61) |
| Poland | 2.32(1.88,2.78) | 0.02(0.02,0.03) |  | 0.46(0.41,0.52) | 0.01(0.01,0.01) |  | -79.94(-84.49,-74.76) | -3.70(-4.04,-3.36) |
| Portugal | 2.36(2.13,2.62) | 0.11(0.10,0.12) |  | 0.46(0.38,0.53) | 0.03(0.03,0.04) |  | -80.61(-83.83,-76.97) | -4.44(-5.05,-3.82) |
| Puerto Rico | 2.74(2.49,3.01) | 0.28(0.25,0.30) |  | 0.10(0.08,0.12) | 0.02(0.02,0.03) |  | -96.42(-97.10,-95.54) | -8.91(-9.51,-8.30) |
| Qatar | 0.13(0.08,0.25) | 0.10(0.06,0.20) |  | 0.20(0.09,0.32) | 0.04(0.02,0.06) |  | 56.55(-48.97,205.47) | -1.24(-2.15,-0.32) |
| Republic of Korea | 13.70(8.83,20.92) | 0.12(0.08,0.18) |  | 1.53(0.95,2.52) | 0.03(0.02,0.04) |  | -88.83(-94.68,-74.60) | -4.67(-4.91,-4.43) |
| Republic of Moldova | 0.03(0.02,0.04) | 0.00(0.00,0.00) |  | 0.02(0.02,0.03) | 0.00(0.00,0.01) |  | -30.63(-49.34,-5.63) | 3.17(0.99,5.41) |
| Romania | 3.60(3.02,4.44) | 0.06(0.05,0.08) |  | 0.96(0.81,1.14) | 0.03(0.03,0.04) |  | -73.27(-80.14,-66.08) | -1.96(-2.29,-1.62) |
| Russian Federation | 51.87(49.01,54.74) | 0.15(0.14,0.16) |  | 5.22(4.70,5.64) | 0.02(0.02,0.02) |  | -89.93(-90.94,-89.03) | -6.09(-6.85,-5.33) |
| Rwanda | 8.04(3.61,20.51) | 0.24(0.11,0.60) |  | 3.09(1.56,6.22) | 0.06(0.03,0.13) |  | -61.62(-79.97,-16.45) | -4.85(-5.20,-4.51) |
| Saint Kitts and Nevis | 0.01(0.01,0.01) | 0.07(0.06,0.10) |  | 0.00(0.00,0.00) | 0.01(0.01,0.02) |  | -85.80(-90.49,-80.35) | -5.94(-6.69,-5.19) |
| Saint Lucia | 0.15(0.12,0.18) | 0.29(0.23,0.35) |  | 0.02(0.01,0.02) | 0.06(0.04,0.08) |  | -88.27(-91.85,-83.11) | -5.97(-6.44,-5.50) |
| Saint Vincent and the Grenadines | 0.03(0.02,0.04) | 0.07(0.06,0.09) |  | 0.01(0.00,0.01) | 0.02(0.01,0.03) |  | -82.94(-88.41,-75.78) | -5.73(-6.82,-4.62) |
| Samoa | 0.05(0.03,0.10) | 0.08(0.05,0.14) |  | 0.04(0.02,0.08) | 0.05(0.03,0.10) |  | -27.65(-62.88,31.45) | -1.28(-1.40,-1.17) |
| San Marino | 0.00(0.00,0.00) | 0.03(0.02,0.05) |  | 0.00(0.00,0.00) | 0.01(0.00,0.01) |  | -75.90(-89.34,-47.15) | -4.34(-4.48,-4.20) |
| Sao Tome and Principe | 0.07(0.03,0.18) | 0.12(0.05,0.31) |  | 0.02(0.01,0.06) | 0.03(0.01,0.07) |  | -66.66(-84.82,-6.63) | -4.11(-4.54,-3.67) |
| Saudi Arabia | 7.61(4.20,13.61) | 0.12(0.06,0.21) |  | 1.06(0.36,3.02) | 0.01(0.00,0.04) |  | -86.02(-96.16,-56.38) | -6.10(-6.66,-5.53) |
| Senegal | 6.26(2.69,14.23) | 0.17(0.07,0.39) |  | 4.66(2.65,8.17) | 0.07(0.04,0.13) |  | -25.52(-58.94,53.57) | -2.30(-2.79,-1.81) |
| Serbia | 0.32(0.17,1.05) | 0.01(0.01,0.05) |  | 0.04(0.01,0.26) | 0.00(0.00,0.02) |  | -87.61(-96.67,-67.33) | -5.28(-5.73,-4.82) |
| Seychelles | 0.01(0.00,0.01) | 0.02(0.01,0.06) |  | 0.00(0.00,0.01) | 0.02(0.01,0.04) |  | -24.25(-53.69,32.46) | 0.12(-0.25,0.50) |
| Sierra Leone | 5.64(2.05,13.81) | 0.31(0.11,0.76) |  | 5.81(3.33,10.82) | 0.16(0.09,0.30) |  | 2.96(-42.63,125.61) | -1.97(-2.17,-1.76) |
| Singapore | 1.71(1.52,1.91) | 0.26(0.23,0.29) |  | 0.43(0.35,0.51) | 0.05(0.04,0.06) |  | -75.03(-80.13,-69.33) | -4.33(-4.80,-3.86) |
| Slovakia | 0.18(0.10,0.66) | 0.01(0.01,0.05) |  | 0.04(0.02,0.19) | 0.00(0.00,0.02) |  | -76.64(-85.94,-62.00) | -2.55(-2.86,-2.25) |
| Slovenia | 0.01(0.01,0.01) | 0.00(0.00,0.00) |  | 0.00(0.00,0.00) | 0.00(0.00,0.00) |  | -62.15(-72.54,-45.93) | -2.61(-2.86,-2.35) |
| Solomon Islands | 0.12(0.06,0.22) | 0.08(0.04,0.14) |  | 0.12(0.06,0.24) | 0.05(0.02,0.09) |  | -1.73(-42.17,61.07) | -1.73(-1.91,-1.55) |
| Somalia | 10.10(3.67,20.63) | 0.26(0.09,0.53) |  | 13.33(4.74,27.98) | 0.13(0.05,0.27) |  | 32.07(-35.21,148.10) | -1.96(-2.21,-1.70) |
| South Africa | 6.35(4.29,9.29) | 0.05(0.03,0.07) |  | 3.44(2.55,4.57) | 0.02(0.02,0.03) |  | -45.81(-63.93,-16.42) | -1.66(-1.97,-1.34) |
| South Sudan | 8.04(2.71,19.48) | 0.31(0.10,0.74) |  | 8.37(4.16,16.53) | 0.19(0.10,0.38) |  | 4.01(-36.15,127.78) | -1.36(-1.77,-0.94) |
| Spain | 6.49(5.88,7.17) | 0.08(0.08,0.09) |  | 2.38(2.02,2.69) | 0.04(0.03,0.04) |  | -63.35(-70.07,-56.89) | -2.59(-3.25,-1.93) |
| Sri Lanka | 9.83(6.90,16.30) | 0.18(0.12,0.29) |  | 4.45(2.89,6.32) | 0.09(0.06,0.12) |  | -54.74(-80.24,-24.90) | -1.45(-2.08,-0.83) |
| Sudan | 49.13(20.43,107.00) | 0.55(0.23,1.20) |  | 54.74(26.61,82.07) | 0.33(0.16,0.49) |  | 11.43(-54.75,152.70) | -0.70(-1.31,-0.09) |
| Suriname | 0.95(0.41,1.29) | 0.73(0.31,0.99) |  | 0.26(0.17,0.40) | 0.18(0.12,0.28) |  | -72.01(-83.19,-31.55) | -4.94(-5.26,-4.61) |
| Sweden | 1.30(1.19,1.40) | 0.08(0.08,0.09) |  | 0.46(0.39,0.53) | 0.03(0.02,0.03) |  | -64.77(-70.14,-58.50) | -3.46(-3.96,-2.95) |
| Switzerland | 1.70(1.28,2.16) | 0.15(0.11,0.19) |  | 0.49(0.41,0.57) | 0.04(0.03,0.04) |  | -71.41(-79.00,-59.06) | -4.55(-4.76,-4.34) |
| Syrian Arab Republic | 6.66(3.06,20.62) | 0.11(0.05,0.35) |  | 0.94(0.39,3.80) | 0.03(0.01,0.10) |  | -85.88(-94.82,-71.85) | -4.75(-5.33,-4.16) |
| Taiwan (Province of China) | 0.32(0.29,0.35) | 0.01(0.01,0.01) |  | 0.30(0.25,0.34) | 0.01(0.01,0.01) |  | -7.81(-24.69,10.88) | 5.33(3.83,6.86) |
| Tajikistan | 7.65(3.53,12.35) | 0.33(0.15,0.53) |  | 6.35(4.14,9.08) | 0.18(0.12,0.25) |  | -16.99(-49.29,58.74) | -1.85(-2.11,-1.59) |
| Thailand | 8.95(5.06,18.85) | 0.05(0.03,0.11) |  | 2.57(1.61,5.54) | 0.03(0.02,0.06) |  | -71.32(-84.74,-49.75) | -2.40(-2.77,-2.04) |
| Timor-Leste | 0.61(0.22,1.75) | 0.18(0.07,0.53) |  | 0.47(0.27,0.94) | 0.09(0.05,0.18) |  | -21.62(-58.46,82.07) | -2.44(-2.66,-2.21) |
| Togo | 2.23(0.89,5.07) | 0.13(0.05,0.29) |  | 2.25(1.39,3.52) | 0.07(0.04,0.11) |  | 0.91(-42.14,102.34) | -1.69(-2.00,-1.38) |
| Tokelau | 0.00(0.00,0.00) | 0.06(0.03,0.11) |  | 0.00(0.00,0.00) | 0.22(0.11,0.56) |  | 143.29(25.48,345.72) | -0.25(-2.00,1.54) |
| Tonga | 0.02(0.02,0.04) | 0.06(0.04,0.10) |  | 0.01(0.01,0.03) | 0.04(0.02,0.08) |  | -38.53(-64.73,-0.29) | -1.27(-1.48,-1.05) |
| Trinidad and Tobago | 1.25(1.05,1.47) | 0.31(0.26,0.36) |  | 0.17(0.13,0.22) | 0.06(0.05,0.08) |  | -86.49(-90.50,-81.36) | -5.54(-6.13,-4.95) |
| Tunisia | 6.98(3.98,12.21) | 0.22(0.13,0.39) |  | 1.93(1.15,2.98) | 0.07(0.04,0.11) |  | -72.32(-89.04,-44.71) | -2.53(-3.02,-2.04) |
| Turkey | 259.86(95.79,465.00) | 1.27(0.47,2.27) |  | 37.63(27.07,50.64) | 0.20(0.15,0.27) |  | -85.52(-92.39,-62.86) | -5.22(-5.48,-4.96) |
| Turkmenistan | 0.43(0.23,0.64) | 0.03(0.02,0.04) |  | 0.19(0.15,0.23) | 0.01(0.01,0.02) |  | -55.83(-69.65,-23.06) | -3.33(-4.06,-2.59) |
| Tuvalu | 0.01(0.00,0.01) | 0.17(0.09,0.32) |  | 0.00(0.00,0.00) | 0.06(0.03,0.11) |  | -64.88(-81.34,-28.99) | -3.38(-3.58,-3.19) |
| Uganda | 21.08(8.00,43.12) | 0.25(0.09,0.51) |  | 16.42(9.27,31.95) | 0.08(0.05,0.16) |  | -22.12(-52.52,46.48) | -3.61(-3.82,-3.41) |
| Ukraine | 2.22(1.57,3.04) | 0.02(0.01,0.03) |  | 0.99(0.80,1.20) | 0.02(0.01,0.02) |  | -55.56(-69.04,-36.77) | -0.15(-0.51,0.22) |
| United Arab Emirates | 2.81(1.69,4.41) | 0.48(0.29,0.75) |  | 0.94(0.60,1.66) | 0.07(0.04,0.12) |  | -66.53(-81.24,-36.59) | -4.19(-4.80,-3.58) |
| United Kingdom | 13.64(10.62,17.19) | 0.12(0.10,0.16) |  | 4.09(3.54,4.56) | 0.03(0.03,0.04) |  | -70.01(-76.46,-61.31) | -3.34(-3.67,-3.01) |
| United Republic of Tanzania | 21.44(10.38,51.35) | 0.18(0.09,0.43) |  | 21.25(12.15,41.11) | 0.09(0.05,0.17) |  | -0.86(-39.43,81.74) | -1.92(-2.11,-1.73) |
| United States of America | 66.75(59.77,75.72) | 0.12(0.11,0.14) |  | 32.66(29.37,36.17) | 0.05(0.05,0.06) |  | -51.08(-58.83,-42.96) | -2.51(-2.71,-2.31) |
| United States Virgin Islands | 0.07(0.04,0.10) | 0.22(0.12,0.30) |  | 0.00(0.00,0.01) | 0.03(0.02,0.04) |  | -94.83(-97.23,-83.82) | -6.69(-7.07,-6.30) |
| Uruguay | 1.15(1.01,1.33) | 0.14(0.12,0.16) |  | 0.18(0.15,0.23) | 0.03(0.02,0.03) |  | -84.00(-87.61,-79.03) | -5.33(-5.60,-5.06) |
| Uzbekistan | 7.05(5.74,8.84) | 0.08(0.07,0.10) |  | 3.87(3.17,4.75) | 0.04(0.03,0.05) |  | -45.17(-58.79,-24.95) | -1.01(-1.76,-0.26) |
| Vanuatu | 0.07(0.04,0.13) | 0.10(0.06,0.19) |  | 0.08(0.04,0.17) | 0.07(0.04,0.14) |  | 10.92(-30.31,81.43) | -1.33(-1.71,-0.95) |
| Venezuela (Bolivarian Republic of) | 4.76(4.03,5.63) | 0.07(0.06,0.08) |  | 1.33(0.92,1.86) | 0.02(0.01,0.03) |  | -72.02(-80.40,-61.15) | -4.31(-4.95,-3.66) |
| Viet Nam | 14.12(8.79,26.15) | 0.05(0.03,0.10) |  | 5.97(3.12,12.36) | 0.02(0.01,0.05) |  | -57.75(-77.86,-21.55) | -1.68(-2.03,-1.32) |
| Yemen | 30.41(14.43,55.64) | 0.43(0.20,0.78) |  | 41.65(23.28,61.84) | 0.30(0.17,0.45) |  | 36.95(-35.89,151.81) | -0.32(-0.86,0.22) |
| Zambia | 5.43(2.37,14.56) | 0.14(0.06,0.39) |  | 4.30(2.63,7.83) | 0.05(0.03,0.09) |  | -20.85(-59.06,72.96) | -2.99(-3.30,-2.68) |
| Zimbabwe | 1.46(0.86,2.32) | 0.03(0.02,0.05) |  | 3.00(1.90,4.82) | 0.05(0.03,0.08) |  | 105.98(25.38,233.21) | 2.79(2.19,3.39) |
