# Supplementary material for: Burden of pulmonary arterial hypertension in children globally, regionally, and nationally (1990–2021): results from the global burden of disease study
Source: Front Pediatr. 2025 Jun 30;13:1527281. doi: 10.3389/fped.2025.1527281 (PMC12256471; doi:10.3389/fped.2025.1527281)
Supplement: Supplementary file 8 [file Table5.docx]

Table S5 DALYs of Pediatric Pulmonary Arterial Hypertension at the national level

| location | 1990 | |  | 2021 | |  | 1990-2021 | |
| --- | --- | --- | --- | --- | --- | --- | --- | --- |
|  | DALYs case | DALYs rate |  | DALYs case | DALYs rate |  | Cases change | EAPC |
| Afghanistan | 1695.86(787.95,2912.40) | 39.36(18.29,67.60) |  | 5235.84(2578.40,7982.23) | 36.87(18.16,56.21) |  | 208.74(36.86,508.01) | 0.45(0.17,0.74) |
| Albania | 127.78(46.76,173.85) | 11.44(4.19,15.56) |  | 17.55(10.84,27.60) | 3.96(2.44,6.22) |  | -86.27(-91.80,-64.85) | -2.77(-3.05,-2.48) |
| Algeria | 2471.43(1376.22,4376.07) | 23.04(12.83,40.80) |  | 1373.60(864.99,1998.42) | 10.33(6.50,15.02) |  | -44.42(-76.88,4.88) | -1.03(-1.60,-0.45) |
| American Samoa | 0.54(0.37,1.06) | 2.85(1.94,5.57) |  | 0.24(0.14,0.58) | 1.67(0.97,4.06) |  | -56.45(-73.67,-34.04) | -1.62(-1.80,-1.45) |
| Andorra | 1.03(0.51,1.64) | 10.80(5.34,17.30) |  | 0.14(0.09,0.21) | 1.42(0.92,2.07) |  | -85.90(-92.59,-68.73) | -6.24(-6.58,-5.90) |
| Angola | 762.94(261.04,2082.43) | 16.18(5.54,44.17) |  | 755.32(459.36,1433.91) | 4.95(3.01,9.41) |  | -1.00(-45.21,137.42) | -3.58(-4.01,-3.16) |
| Antigua and Barbuda | 0.81(0.64,1.07) | 4.45(3.52,5.85) |  | 0.14(0.12,0.17) | 0.85(0.72,0.99) |  | -82.16(-86.39,-76.80) | -6.36(-6.98,-5.73) |
| Argentina | 3001.71(2602.82,3484.61) | 29.62(25.68,34.38) |  | 381.87(310.83,456.16) | 3.75(3.05,4.48) |  | -87.28(-90.21,-83.77) | -6.15(-6.46,-5.85) |
| Armenia | 24.21(19.70,31.11) | 2.32(1.89,2.98) |  | 1.40(1.15,1.68) | 0.24(0.19,0.28) |  | -94.21(-95.79,-92.27) | -6.91(-7.76,-6.05) |
| Australia | 303.30(234.55,382.23) | 8.01(6.20,10.10) |  | 97.64(78.56,121.25) | 2.06(1.65,2.55) |  | -67.81(-76.50,-56.19) | -3.88(-4.50,-3.27) |
| Austria | 49.33(43.25,56.19) | 3.66(3.21,4.17) |  | 17.36(14.93,20.01) | 1.34(1.15,1.54) |  | -64.80(-71.41,-56.92) | -2.47(-3.10,-1.83) |
| Azerbaijan | 208.45(93.33,367.26) | 8.59(3.85,15.13) |  | 71.45(50.99,98.83) | 3.03(2.16,4.19) |  | -65.72(-82.46,-8.60) | -3.14(-3.28,-2.99) |
| Bahamas | 30.80(25.44,37.52) | 38.19(31.54,46.52) |  | 4.54(3.50,5.85) | 5.59(4.31,7.20) |  | -85.27(-89.34,-79.91) | -7.03(-7.62,-6.44) |
| Bahrain | 18.53(12.37,33.20) | 11.35(7.58,20.34) |  | 12.02(5.79,19.84) | 4.05(1.95,6.69) |  | -35.12(-71.76,11.84) | -1.72(-2.82,-0.61) |
| Bangladesh | 7775.88(2678.01,13968.43) | 15.90(5.48,28.56) |  | 2688.64(1521.42,4101.66) | 5.87(3.32,8.96) |  | -65.42(-81.62,-27.01) | -2.76(-2.95,-2.56) |
| Barbados | 17.99(14.89,21.49) | 28.84(23.88,34.46) |  | 2.96(2.18,3.97) | 6.28(4.63,8.43) |  | -83.55(-88.27,-77.73) | -5.42(-5.90,-4.94) |
| Belarus | 79.42(51.10,122.32) | 3.30(2.13,5.09) |  | 17.28(13.63,21.62) | 1.10(0.86,1.37) |  | -78.24(-87.16,-64.49) | -2.84(-3.20,-2.49) |
| Belgium | 133.75(96.30,181.60) | 7.41(5.33,10.05) |  | 50.04(41.58,59.35) | 2.62(2.17,3.10) |  | -62.59(-73.56,-46.39) | -3.14(-3.49,-2.79) |
| Belize | 35.92(28.57,43.31) | 43.88(34.90,52.90) |  | 4.70(3.77,5.71) | 3.82(3.07,4.64) |  | -86.91(-90.17,-82.51) | -8.42(-8.95,-7.89) |
| Benin | 315.10(130.15,719.69) | 13.01(5.37,29.72) |  | 495.78(301.17,779.00) | 8.15(4.95,12.81) |  | 57.34(-4.80,215.73) | -1.01(-1.21,-0.80) |
| Bermuda | 4.21(3.40,5.24) | 35.31(28.52,43.97) |  | 0.39(0.26,0.52) | 4.58(3.06,6.19) |  | -90.82(-93.62,-86.84) | -6.80(-7.15,-6.46) |
| Bhutan | 67.51(25.01,105.05) | 25.75(9.54,40.06) |  | 16.74(10.39,26.77) | 8.94(5.55,14.30) |  | -75.20(-86.42,-37.54) | -3.55(-3.80,-3.29) |
| Bolivia (Plurinational State of) | 1298.74(519.81,2291.08) | 48.35(19.35,85.30) |  | 522.32(336.70,771.86) | 14.98(9.66,22.14) |  | -59.78(-75.43,-14.49) | -3.55(-3.63,-3.47) |
| Bosnia and Herzegovina | 18.71(10.46,47.00) | 1.71(0.95,4.29) |  | 4.55(2.69,10.78) | 0.93(0.55,2.20) |  | -75.67(-85.48,-56.30) | -1.97(-2.23,-1.71) |
| Botswana | 21.87(14.16,31.93) | 3.70(2.40,5.41) |  | 25.13(15.64,39.26) | 3.60(2.24,5.62) |  | 14.92(-33.93,92.65) | 0.74(0.33,1.16) |
| Brazil | 8217.46(7146.89,9447.10) | 15.82(13.76,18.19) |  | 3333.72(2706.40,4072.12) | 6.92(5.62,8.45) |  | -59.43(-69.06,-49.08) | -2.11(-2.88,-1.35) |
| Brunei Darussalam | 28.83(15.37,39.78) | 31.82(16.97,43.91) |  | 14.25(8.42,20.58) | 15.06(8.90,21.76) |  | -50.58(-67.51,-21.57) | -2.27(-2.45,-2.08) |
| Bulgaria | 33.66(28.64,39.59) | 1.94(1.65,2.28) |  | 6.01(4.82,7.45) | 0.62(0.49,0.76) |  | -82.13(-86.39,-76.54) | -2.68(-3.24,-2.13) |
| Burkina Faso | 771.01(291.70,1583.66) | 16.34(6.18,33.56) |  | 1253.97(693.94,2025.72) | 12.09(6.69,19.53) |  | 62.64(0.48,197.20) | -0.56(-0.75,-0.38) |
| Burundi | 635.77(285.59,1501.16) | 24.26(10.90,57.27) |  | 441.96(229.74,802.46) | 7.55(3.92,13.71) |  | -30.48(-66.04,37.74) | -3.07(-3.50,-2.63) |
| Cabo Verde | 19.91(9.72,37.54) | 12.66(6.18,23.86) |  | 4.58(1.83,9.83) | 3.20(1.28,6.87) |  | -76.99(-88.40,-53.66) | -4.56(-4.73,-4.38) |
| Cambodia | 435.87(174.49,1217.00) | 9.35(3.74,26.11) |  | 233.65(139.88,433.04) | 4.57(2.73,8.46) |  | -46.39(-72.03,16.68) | -2.24(-2.29,-2.19) |
| Cameroon | 488.00(223.64,1097.80) | 10.00(4.58,22.49) |  | 951.84(608.82,1578.14) | 7.07(4.52,11.72) |  | 95.05(24.53,237.38) | -0.35(-0.85,0.15) |
| Canada | 835.21(751.70,914.75) | 14.52(13.07,15.90) |  | 223.31(188.33,268.46) | 3.62(3.05,4.35) |  | -73.26(-77.68,-67.65) | -5.00(-5.50,-4.49) |
| Central African Republic | 207.00(78.46,518.50) | 16.93(6.42,42.41) |  | 210.79(117.20,396.26) | 9.23(5.13,17.35) |  | 1.83(-38.36,105.31) | -1.73(-1.86,-1.61) |
| Chad | 459.50(139.21,1064.39) | 15.70(4.76,36.37) |  | 1247.97(532.12,2424.63) | 13.84(5.90,26.90) |  | 171.59(79.57,385.70) | -0.20(-0.37,-0.03) |
| Chile | 474.10(421.36,528.55) | 11.94(10.61,13.31) |  | 114.98(96.44,134.30) | 3.15(2.64,3.68) |  | -75.75(-80.64,-70.48) | -3.81(-4.20,-3.42) |
| China | 56420.97(37801.94,83698.93) | 17.72(11.87,26.29) |  | 8623.35(5310.91,13123.36) | 3.32(2.05,5.05) |  | -84.72(-92.89,-72.83) | -3.68(-4.35,-3.01) |
| Colombia | 928.20(751.04,1200.21) | 7.96(6.44,10.29) |  | 264.97(194.85,360.50) | 2.50(1.84,3.40) |  | -71.45(-81.09,-58.82) | -3.19(-3.68,-2.70) |
| Comoros | 41.73(18.24,84.25) | 19.62(8.57,39.61) |  | 15.65(8.01,33.51) | 6.52(3.34,13.95) |  | -62.50(-78.46,-31.32) | -3.67(-3.87,-3.48) |
| Congo | 92.95(42.32,229.93) | 8.83(4.02,21.84) |  | 54.36(37.40,100.83) | 2.82(1.94,5.23) |  | -41.51(-63.76,10.40) | -3.69(-4.16,-3.21) |
| Cook Islands | 0.74(0.46,1.35) | 11.15(6.93,20.53) |  | 0.22(0.06,0.52) | 5.76(1.48,13.79) |  | -70.34(-90.80,-30.49) | -4.32(-5.06,-3.58) |
| Costa Rica | 114.31(102.32,126.99) | 10.17(9.10,11.30) |  | 17.96(14.58,21.86) | 1.77(1.43,2.15) |  | -84.29(-87.54,-80.48) | -4.67(-5.43,-3.90) |
| Croatia | 738.14(341.75,1617.59) | 12.94(5.99,28.36) |  | 1034.76(677.31,1705.86) | 8.94(5.85,14.74) |  | -84.21(-87.03,-80.95) | -0.60(-0.85,-0.35) |
| Cuba | 9.13(8.16,10.20) | 0.93(0.83,1.03) |  | 1.44(1.19,1.71) | 0.24(0.20,0.29) |  | -87.84(-90.56,-84.34) | -3.32(-4.20,-2.43) |
| Cyprus | 148.98(124.43,176.29) | 5.95(4.97,7.04) |  | 18.12(15.05,21.74) | 1.02(0.85,1.22) |  | -83.28(-89.86,-67.59) | -5.91(-6.45,-5.37) |
| Czechia | 86.89(53.74,126.05) | 43.90(27.15,63.68) |  | 14.52(9.82,20.15) | 6.64(4.49,9.21) |  | -68.31(-77.61,-57.70) | -5.61(-5.94,-5.29) |
| C么te d'Ivoire | 80.44(65.18,100.24) | 3.65(2.96,4.55) |  | 25.49(20.69,30.90) | 1.49(1.21,1.80) |  | 40.18(-9.20,161.79) | -2.89(-3.19,-2.59) |
| Democratic People's Republic of Korea | 458.28(286.53,871.01) | 7.70(4.82,14.64) |  | 150.36(68.23,292.04) | 3.15(1.43,6.12) |  | -67.19(-85.69,-38.71) | -2.55(-2.84,-2.27) |
| Democratic Republic of the Congo | 2497.92(1043.27,5215.02) | 14.11(5.89,29.46) |  | 1543.43(894.76,2556.99) | 4.06(2.35,6.73) |  | -38.21(-69.25,28.62) | -3.47(-3.84,-3.11) |
| Denmark | 102.19(86.22,116.83) | 11.57(9.76,13.23) |  | 30.04(25.28,35.23) | 3.15(2.65,3.69) |  | -70.60(-76.22,-63.21) | -4.16(-4.52,-3.80) |
| Djibouti | 25.08(9.69,60.86) | 14.40(5.56,34.95) |  | 23.94(11.42,50.70) | 5.79(2.77,12.27) |  | -4.56(-49.68,97.76) | -2.90(-3.27,-2.52) |
| Dominica | 1.28(0.74,1.85) | 5.15(2.99,7.46) |  | 0.35(0.21,0.51) | 2.54(1.55,3.73) |  | -72.76(-84.60,-45.92) | -2.83(-3.26,-2.41) |
| Dominican Republic | 637.72(277.30,947.99) | 23.66(10.29,35.17) |  | 198.76(121.38,316.78) | 6.76(4.13,10.78) |  | -68.83(-83.35,-18.04) | -4.58(-4.86,-4.30) |
| Ecuador | 590.57(456.63,791.46) | 15.28(11.81,20.48) |  | 355.02(271.39,461.58) | 7.00(5.35,9.10) |  | -39.89(-60.31,-14.80) | -1.94(-2.58,-1.29) |
| Egypt | 53050.16(17787.15,80879.56) | 239.16(80.19,364.62) |  | 11630.65(8581.96,15876.52) | 31.56(23.29,43.08) |  | -78.08(-86.53,-41.46) | -5.63(-6.04,-5.23) |
| El Salvador | 436.61(239.78,641.06) | 20.23(11.11,29.71) |  | 60.33(37.16,113.25) | 3.32(2.04,6.23) |  | -86.18(-93.52,-55.31) | -6.17(-6.49,-5.84) |
| Equatorial Guinea | 25.07(9.43,57.81) | 12.73(4.79,29.36) |  | 16.68(9.37,29.53) | 2.85(1.60,5.05) |  | -33.48(-63.80,81.32) | -5.37(-5.68,-5.06) |
| Eritrea | 273.49(109.02,702.44) | 17.18(6.85,44.13) |  | 192.31(93.08,411.82) | 7.62(3.69,16.31) |  | -29.68(-60.01,34.01) | -2.52(-2.67,-2.38) |
| Estonia | 4.23(3.75,4.81) | 1.21(1.07,1.38) |  | 0.65(0.55,0.76) | 0.30(0.25,0.35) |  | -84.74(-87.44,-81.81) | -2.90(-4.69,-1.08) |
| Eswatini | 19.49(12.00,29.26) | 5.05(3.11,7.59) |  | 14.76(10.10,20.91) | 3.58(2.45,5.07) |  | -24.24(-51.58,26.42) | -0.55(-0.77,-0.33) |
| Ethiopia | 3418.46(1382.07,8497.18) | 14.03(5.67,34.88) |  | 2316.20(1230.82,4929.43) | 5.22(2.78,11.11) |  | -32.24(-59.76,32.27) | -3.48(-3.66,-3.31) |
| Fiji | 14.53(8.87,29.19) | 5.16(3.15,10.37) |  | 12.13(6.63,25.83) | 4.45(2.43,9.48) |  | -16.48(-48.78,34.31) | -0.37(-0.60,-0.15) |
| Finland | 26.23(22.52,31.13) | 2.72(2.33,3.23) |  | 10.47(9.01,12.04) | 1.24(1.06,1.42) |  | -60.10(-68.52,-51.58) | -2.41(-3.22,-1.60) |
| France | 755.74(581.88,997.83) | 6.45(4.97,8.52) |  | 408.99(353.51,473.37) | 3.52(3.05,4.08) |  | -45.88(-61.66,-25.22) | -2.02(-2.39,-1.65) |
| Gabon | 25.13(12.97,52.30) | 6.17(3.18,12.83) |  | 14.48(9.14,24.31) | 2.26(1.43,3.80) |  | -42.39(-67.94,15.93) | -2.53(-2.91,-2.15) |
| Gambia | 48.00(21.16,111.24) | 10.40(4.59,24.12) |  | 57.50(35.59,105.39) | 5.79(3.58,10.61) |  | 19.79(-24.27,117.34) | -1.89(-2.25,-1.54) |
| Georgia | 43.20(32.87,57.22) | 3.16(2.40,4.18) |  | 10.84(8.52,13.47) | 1.47(1.16,1.83) |  | -74.92(-82.47,-64.57) | -0.93(-3.03,1.22) |
| Germany | 1245.99(1051.15,1471.49) | 9.62(8.12,11.37) |  | 432.28(367.55,494.89) | 3.61(3.07,4.14) |  | -65.31(-72.54,-56.86) | -2.41(-3.06,-1.76) |
| Ghana | 503.88(220.94,1273.70) | 7.50(3.29,18.96) |  | 370.09(195.71,707.32) | 2.87(1.52,5.49) |  | -26.55(-51.21,23.66) | -2.74(-2.91,-2.57) |
| Greece | 136.34(123.01,148.99) | 6.74(6.08,7.36) |  | 61.42(51.23,71.96) | 4.40(3.67,5.16) |  | -54.95(-63.01,-46.35) | -1.22(-1.76,-0.68) |
| Greenland | 10.23(2.62,16.44) | 71.95(18.43,115.57) |  | 1.20(0.64,1.74) | 10.24(5.47,14.83) |  | -88.24(-95.28,-44.30) | -6.40(-6.85,-5.94) |
| Grenada | 11.13(8.86,13.76) | 33.31(26.53,41.17) |  | 1.16(0.93,1.41) | 5.30(4.27,6.47) |  | -89.61(-92.08,-86.24) | -6.22(-6.72,-5.73) |
| Guam | 1.63(1.06,3.31) | 3.91(2.54,7.94) |  | 1.03(0.54,2.47) | 2.82(1.48,6.74) |  | -36.77(-60.67,-7.80) | 0.02(-0.37,0.40) |
| Guatemala | 608.40(509.11,773.70) | 14.98(12.54,19.05) |  | 129.22(101.95,164.18) | 2.62(2.07,3.33) |  | -78.76(-85.07,-70.82) | -6.31(-6.79,-5.83) |
| Guinea | 554.36(181.63,1340.31) | 20.14(6.60,48.71) |  | 677.38(390.85,1099.26) | 11.20(6.46,18.18) |  | 22.19(-29.06,170.24) | -1.25(-1.51,-0.99) |
| Guinea-Bissau | 79.32(26.79,189.34) | 16.44(5.55,39.25) |  | 70.27(39.24,119.77) | 7.82(4.37,13.34) |  | -11.40(-49.82,95.49) | -1.89(-2.35,-1.43) |
| Guyana | 54.72(44.77,66.18) | 18.62(15.23,22.51) |  | 28.18(21.20,37.38) | 13.21(9.94,17.52) |  | -48.49(-63.44,-28.15) | -2.93(-4.30,-1.54) |
| Haiti | 3138.38(811.76,6197.71) | 115.68(29.92,228.44) |  | 2358.40(751.17,4495.43) | 54.18(17.26,103.28) |  | -24.85(-52.46,39.96) | -2.15(-2.33,-1.96) |
| Honduras | 190.06(108.85,279.03) | 8.60(4.93,12.63) |  | 92.66(49.71,174.59) | 2.83(1.52,5.33) |  | -51.25(-72.48,28.73) | -3.57(-3.71,-3.44) |
| Hungary | 48.67(43.74,53.80) | 2.28(2.05,2.52) |  | 7.04(5.92,8.18) | 0.51(0.43,0.59) |  | -85.54(-88.01,-82.51) | -3.38(-4.29,-2.46) |
| Iceland | 4.37(3.83,4.93) | 6.88(6.04,7.77) |  | 2.02(1.66,2.42) | 2.99(2.46,3.58) |  | -53.69(-63.25,-42.89) | -3.45(-4.18,-2.71) |
| India | 62327.56(25053.01,91646.39) | 19.09(7.67,28.07) |  | 26219.41(15800.59,42144.66) | 7.16(4.31,11.50) |  | -57.93(-70.02,-32.47) | -2.87(-3.06,-2.69) |
| Indonesia | 4845.48(1961.77,14955.23) | 7.15(2.90,22.08) |  | 2995.17(1707.08,6399.95) | 4.45(2.54,9.51) |  | -38.19(-62.33,14.12) | -1.59(-1.73,-1.45) |
| Iran (Islamic Republic of) | 18662.03(10512.79,31740.21) | 73.52(41.41,125.04) |  | 1413.97(990.02,2589.25) | 7.01(4.91,12.83) |  | -92.42(-95.60,-76.20) | -5.17(-6.01,-4.32) |
| Iraq | 1810.49(739.68,2758.40) | 21.98(8.98,33.49) |  | 759.33(394.77,1182.96) | 5.64(2.93,8.79) |  | -58.06(-77.06,-11.51) | -4.04(-4.29,-3.79) |
| Ireland | 34.55(31.28,37.95) | 3.52(3.18,3.86) |  | 14.82(12.42,17.22) | 1.49(1.25,1.73) |  | -57.10(-64.35,-47.94) | -2.60(-3.59,-1.60) |
| Israel | 304.07(240.77,401.88) | 19.83(15.70,26.21) |  | 115.33(97.66,134.51) | 4.39(3.72,5.12) |  | -62.07(-72.99,-50.93) | -3.97(-4.50,-3.44) |
| Italy | 473.83(446.70,504.60) | 5.13(4.84,5.47) |  | 94.64(82.77,106.33) | 1.25(1.09,1.40) |  | -80.03(-82.90,-77.04) | -5.16(-5.75,-4.57) |
| Jamaica | 126.80(104.02,152.86) | 15.18(12.45,18.30) |  | 10.97(8.17,14.59) | 1.88(1.40,2.50) |  | -91.35(-93.96,-87.65) | -7.31(-7.70,-6.92) |
| Japan | 4303.19(4150.79,4463.22) | 18.64(17.98,19.33) |  | 1743.94(1559.37,1913.31) | 11.29(10.10,12.39) |  | -59.47(-63.70,-55.02) | -2.34(-2.95,-1.74) |
| Jordan | 93.98(49.20,335.74) | 5.75(3.01,20.55) |  | 137.80(61.41,348.37) | 3.79(1.69,9.59) |  | 46.63(-49.70,206.98) | -0.09(-0.71,0.54) |
| Kazakhstan | 19.32(13.63,26.95) | 0.37(0.26,0.52) |  | 9.38(7.33,11.72) | 0.17(0.14,0.22) |  | -51.44(-66.23,-29.99) | -3.31(-3.71,-2.91) |
| Kenya | 649.62(332.02,1475.08) | 5.82(2.97,13.21) |  | 488.21(266.75,964.64) | 2.62(1.43,5.17) |  | -24.85(-53.95,31.97) | -1.98(-2.22,-1.74) |
| Kiribati | 2.97(1.35,6.71) | 10.05(4.58,22.74) |  | 2.39(1.23,4.95) | 5.69(2.92,11.78) |  | -19.46(-48.35,31.39) | -1.64(-1.75,-1.52) |
| Kuwait | 51.00(43.83,59.29) | 9.20(7.91,10.70) |  | 102.82(81.30,127.47) | 12.16(9.62,15.08) |  | 101.62(54.39,161.46) | 3.00(1.17,4.87) |
| Kyrgyzstan | 19.11(15.17,23.47) | 1.14(0.90,1.40) |  | 4.85(4.03,5.86) | 0.21(0.18,0.26) |  | -74.63(-81.07,-65.77) | -5.65(-6.11,-5.20) |
| Lao People's Democratic Republic | 314.32(91.58,1002.02) | 17.05(4.97,54.37) |  | 200.90(104.86,436.37) | 8.75(4.57,19.00) |  | -36.08(-64.79,58.99) | -2.27(-2.37,-2.17) |
| Latvia | 6.41(5.57,7.36) | 1.13(0.98,1.29) |  | 2.09(1.76,2.50) | 0.70(0.59,0.84) |  | -67.45(-74.01,-59.74) | -0.70(-2.68,1.32) |
| Lebanon | 446.77(205.46,785.90) | 42.72(19.64,75.14) |  | 146.04(95.94,210.95) | 11.43(7.51,16.51) |  | -67.31(-85.79,-17.60) | -3.91(-4.25,-3.57) |
| Lesotho | 21.57(13.31,36.16) | 3.16(1.95,5.30) |  | 21.04(12.90,30.90) | 3.34(2.05,4.90) |  | -2.46(-42.07,60.90) | 0.89(0.57,1.22) |
| Liberia | 261.33(100.60,651.12) | 23.12(8.90,57.61) |  | 172.61(97.40,292.34) | 7.90(4.46,13.37) |  | -33.95(-69.16,70.05) | -2.89(-3.18,-2.59) |
| Libya | 1023.87(557.85,1926.82) | 56.54(30.80,106.40) |  | 583.45(236.08,1047.82) | 39.12(15.83,70.25) |  | -43.02(-74.55,4.80) | 0.10(-0.33,0.52) |
| Lithuania | 17.14(15.25,19.14) | 2.06(1.84,2.30) |  | 3.49(3.04,3.98) | 0.85(0.75,0.98) |  | -79.67(-82.72,-76.02) | -1.60(-3.23,0.06) |
| Luxembourg | 4.87(4.28,5.44) | 7.36(6.48,8.23) |  | 2.26(1.84,2.73) | 2.23(1.82,2.69) |  | -53.61(-63.87,-41.57) | -4.29(-4.67,-3.90) |
| Madagascar | 1474.46(615.68,3828.63) | 27.02(11.28,70.17) |  | 1427.27(668.76,2946.27) | 12.16(5.70,25.11) |  | -3.20(-39.52,78.81) | -2.21(-2.42,-2.00) |
| Malawi | 846.66(339.84,1959.86) | 18.61(7.47,43.08) |  | 427.14(239.88,742.09) | 5.26(2.95,9.13) |  | -49.55(-72.58,7.87) | -3.95(-4.17,-3.73) |
| Malaysia | 186.82(119.39,424.80) | 2.84(1.82,6.46) |  | 104.01(65.15,287.82) | 1.37(0.86,3.78) |  | -44.33(-68.57,-6.19) | -1.52(-2.27,-0.75) |
| Maldives | 37.61(16.05,56.10) | 35.80(15.28,53.41) |  | 7.76(5.69,10.59) | 7.74(5.68,10.57) |  | -79.38(-88.00,-50.44) | -3.99(-4.40,-3.58) |
| Mali | 568.01(186.66,1341.12) | 13.75(4.52,32.47) |  | 952.89(505.28,1718.34) | 8.23(4.37,14.84) |  | 67.76(3.67,227.28) | -1.37(-1.56,-1.17) |
| Malta | 2.63(2.25,3.02) | 3.00(2.57,3.45) |  | 1.66(1.31,2.04) | 2.59(2.05,3.19) |  | -36.81(-49.98,-20.16) | -0.41(-0.91,0.09) |
| Marshall Islands | 1.25(0.76,2.20) | 5.69(3.48,10.03) |  | 0.84(0.47,1.75) | 4.79(2.66,10.02) |  | -32.96(-59.63,8.43) | -0.61(-1.07,-0.16) |
| Mauritania | 72.97(27.47,174.52) | 7.89(2.97,18.88) |  | 75.74(45.73,129.44) | 4.09(2.47,6.99) |  | 3.79(-37.00,110.81) | -1.96(-2.42,-1.50) |
| Mauritius | 33.22(29.42,37.39) | 10.06(8.91,11.33) |  | 35.15(29.30,40.74) | 16.95(14.13,19.64) |  | 5.81(-13.15,29.59) | 8.26(5.66,10.91) |
| Mexico | 3271.48(2495.59,4148.22) | 9.79(7.47,12.41) |  | 879.03(698.66,1098.88) | 2.74(2.18,3.43) |  | -73.13(-80.82,-61.78) | -4.08(-4.78,-3.38) |
| Micronesia (Federated States of) | 3.22(1.88,6.05) | 7.01(4.10,13.17) |  | 1.07(0.65,2.27) | 3.49(2.12,7.43) |  | -66.80(-81.47,-43.30) | -2.19(-2.26,-2.12) |
| Monaco | 0.12(0.07,0.23) | 3.52(1.96,6.63) |  | 0.12(0.07,0.18) | 2.35(1.33,3.61) |  | -5.65(-68.65,95.90) | -2.82(-3.36,-2.28) |
| Mongolia | 119.50(57.74,221.91) | 13.28(6.42,24.66) |  | 47.81(32.57,68.05) | 4.40(3.00,6.26) |  | -59.99(-81.18,-10.49) | -2.64(-3.20,-2.08) |
| Montenegro | 1.15(0.46,8.12) | 0.71(0.28,5.02) |  | 0.29(0.10,2.02) | 0.26(0.09,1.82) |  | -74.68(-87.31,-55.01) | -3.43(-3.80,-3.05) |
| Morocco | 3590.61(1871.04,6567.77) | 36.69(19.12,67.12) |  | 1349.87(797.13,2151.23) | 13.79(8.14,21.97) |  | -62.41(-84.14,-32.60) | -2.22(-2.84,-1.59) |
| Mozambique | 1027.15(405.60,2152.05) | 16.56(6.54,34.69) |  | 828.99(466.16,1356.13) | 5.81(3.27,9.51) |  | -19.29(-55.36,75.82) | -3.14(-3.35,-2.92) |
| Myanmar | 2230.22(746.81,6749.31) | 15.09(5.05,45.68) |  | 1336.07(767.44,2761.68) | 8.56(4.91,17.69) |  | -40.09(-64.53,41.01) | -1.91(-2.14,-1.69) |
| Namibia | 21.54(13.94,32.76) | 3.58(2.32,5.45) |  | 23.57(15.45,36.87) | 2.86(1.87,4.47) |  | 9.46(-35.47,86.80) | 0.02(-0.41,0.46) |
| Nauru | 0.39(0.23,0.76) | 9.17(5.39,18.00) |  | 0.32(0.18,0.67) | 8.06(4.47,16.96) |  | -17.12(-47.43,28.63) | -0.43(-1.02,0.16) |
| Nepal | 2551.99(988.66,3972.96) | 30.29(11.73,47.16) |  | 793.49(440.67,1389.22) | 8.60(4.78,15.06) |  | -68.91(-85.05,-33.23) | -4.05(-4.21,-3.89) |
| Netherlands | 134.39(123.99,146.28) | 4.93(4.55,5.37) |  | 61.40(53.91,68.97) | 2.29(2.01,2.57) |  | -54.31(-60.07,-47.16) | -2.83(-3.54,-2.12) |
| New Zealand | 18.89(16.64,21.31) | 2.36(2.08,2.66) |  | 9.75(8.14,11.61) | 0.99(0.83,1.18) |  | -48.40(-57.97,-37.39) | -3.13(-4.27,-1.98) |
| Nicaragua | 214.87(91.27,316.28) | 11.80(5.01,17.37) |  | 29.93(16.31,88.04) | 1.51(0.82,4.45) |  | -86.07(-93.85,-43.70) | -6.71(-6.87,-6.54) |
| Niger | 702.31(247.65,1576.41) | 17.29(6.10,38.80) |  | 1172.88(566.72,2127.40) | 9.19(4.44,16.67) |  | 67.00(-16.51,249.83) | -2.17(-2.41,-1.92) |
| Nigeria | 6289.88(2338.04,16418.70) | 16.08(5.98,41.97) |  | 10570.52(6319.90,18078.60) | 10.41(6.22,17.80) |  | 68.06(6.96,247.69) | -1.06(-1.40,-0.72) |
| Niue | 0.05(0.03,0.10) | 6.11(3.99,12.21) |  | 0.07(0.04,0.15) | 18.56(11.34,38.70) |  | 45.83(-6.68,120.08) | 0.92(-0.05,1.89) |
| North Macedonia | 13.81(6.99,28.44) | 2.62(1.33,5.40) |  | 1.55(0.80,6.18) | 0.47(0.25,1.89) |  | -88.81(-94.83,-72.83) | -4.37(-4.96,-3.78) |
| Northern Mariana Islands | 0.24(0.12,0.58) | 1.99(1.01,4.76) |  | 0.12(0.07,0.28) | 1.10(0.65,2.49) |  | -48.76(-67.67,-20.13) | -1.79(-2.21,-1.37) |
| Norway | 112.21(105.45,119.73) | 14.06(13.21,15.00) |  | 12.09(10.77,13.56) | 1.31(1.17,1.47) |  | -89.22(-90.53,-87.82) | -8.30(-9.22,-7.37) |
| Oman | 60.21(29.80,162.45) | 7.16(3.55,19.33) |  | 48.99(15.82,80.99) | 4.01(1.29,6.62) |  | -18.64(-80.54,91.64) | 0.75(-1.08,2.61) |
| Pakistan | 15175.09(5971.36,23499.25) | 30.82(12.13,47.72) |  | 15403.36(7433.13,24780.73) | 18.03(8.70,29.00) |  | 1.50(-33.18,48.46) | -0.73(-1.20,-0.26) |
| Palau | 0.25(0.13,0.49) | 5.51(2.84,10.65) |  | 0.10(0.05,0.21) | 3.04(1.62,6.38) |  | -60.49(-77.33,-33.05) | -1.71(-1.91,-1.50) |
| Palestine | 245.72(98.06,407.97) | 25.38(10.13,42.13) |  | 128.56(50.40,267.15) | 6.89(2.70,14.31) |  | -47.68(-73.86,7.95) | -3.35(-3.70,-3.00) |
| Panama | 53.51(44.14,64.28) | 6.42(5.29,7.71) |  | 15.32(12.05,19.08) | 1.33(1.04,1.65) |  | -71.37(-78.81,-61.56) | -5.77(-6.48,-5.06) |
| Papua New Guinea | 334.67(178.75,582.09) | 19.69(10.51,34.24) |  | 703.91(396.66,1232.81) | 17.97(10.13,31.47) |  | 110.33(35.60,226.90) | -0.13(-0.30,0.03) |
| Paraguay | 62.20(41.52,123.32) | 3.73(2.49,7.39) |  | 35.20(18.73,95.63) | 1.75(0.93,4.76) |  | -43.41(-68.99,2.40) | -2.20(-2.49,-1.92) |
| Peru | 1794.18(860.63,2961.53) | 21.61(10.37,35.68) |  | 613.75(398.49,886.98) | 6.44(4.18,9.30) |  | -65.79(-84.05,-16.85) | -3.21(-3.41,-3.00) |
| Philippines | 1807.87(1104.84,3263.66) | 7.17(4.38,12.94) |  | 910.19(647.81,2291.21) | 2.68(1.91,6.74) |  | -49.65(-66.51,-22.53) | -2.84(-3.05,-2.63) |
| Poland | 204.00(166.17,244.31) | 2.13(1.74,2.55) |  | 42.50(37.61,47.37) | 0.72(0.64,0.80) |  | -79.17(-83.74,-74.06) | -3.58(-3.91,-3.25) |
| Portugal | 205.05(184.50,226.95) | 9.69(8.72,10.73) |  | 39.99(33.35,45.96) | 2.94(2.45,3.37) |  | -80.50(-83.78,-76.95) | -4.42(-5.02,-3.81) |
| Puerto Rico | 241.53(219.07,264.72) | 24.26(22.00,26.58) |  | 8.93(7.38,10.94) | 2.01(1.66,2.46) |  | -96.30(-96.99,-95.40) | -8.80(-9.40,-8.20) |
| Qatar | 11.30(6.94,22.41) | 9.04(5.55,17.92) |  | 17.67(8.24,28.21) | 3.58(1.67,5.71) |  | 56.36(-48.12,204.16) | -1.26(-2.16,-0.35) |
| Republic of Korea | 1195.53(773.80,1824.07) | 10.51(6.81,16.04) |  | 134.97(84.41,222.78) | 2.22(1.39,3.67) |  | -88.71(-94.58,-74.79) | -4.63(-4.87,-4.39) |
| Republic of Moldova | 3.51(2.92,4.17) | 0.28(0.24,0.34) |  | 2.21(1.76,2.80) | 0.42(0.34,0.54) |  | -37.11(-50.78,-17.83) | 2.57(0.72,4.45) |
| Romania | 310.98(262.17,382.57) | 5.59(4.71,6.87) |  | 82.42(70.18,96.94) | 2.74(2.33,3.22) |  | -73.50(-80.17,-66.56) | -1.96(-2.28,-1.64) |
| Russian Federation | 4531.57(4279.59,4776.69) | 13.06(12.33,13.77) |  | 469.36(424.36,508.25) | 1.80(1.63,1.95) |  | -89.64(-90.59,-88.70) | -6.00(-6.73,-5.26) |
| Rwanda | 698.34(311.71,1786.89) | 20.58(9.19,52.67) |  | 267.83(133.02,542.30) | 5.39(2.68,10.91) |  | -61.65(-79.97,-16.67) | -4.85(-5.19,-4.50) |
| Saint Kitts and Nevis | 0.91(0.72,1.20) | 6.47(5.11,8.53) |  | 0.13(0.10,0.17) | 1.35(1.05,1.74) |  | -85.47(-90.15,-79.90) | -5.86(-6.59,-5.12) |
| Saint Lucia | 13.03(10.41,15.93) | 25.28(20.20,30.90) |  | 1.52(1.11,2.10) | 5.12(3.75,7.06) |  | -88.33(-91.90,-83.18) | -5.95(-6.40,-5.50) |
| Saint Vincent and the Grenadines | 2.61(2.09,3.27) | 6.35(5.09,7.96) |  | 0.45(0.32,0.59) | 1.79(1.30,2.38) |  | -82.84(-88.32,-75.71) | -5.70(-6.77,-4.61) |
| Samoa | 4.74(2.88,8.93) | 6.65(4.04,12.53) |  | 3.42(1.78,7.16) | 4.27(2.22,8.96) |  | -27.88(-62.83,31.11) | -1.29(-1.41,-1.18) |
| San Marino | 0.10(0.06,0.17) | 2.47(1.52,4.11) |  | 0.03(0.01,0.05) | 0.60(0.33,1.21) |  | -73.83(-87.52,-45.19) | -4.14(-4.27,-4.01) |
| Sao Tome and Principe | 5.90(2.40,15.48) | 10.41(4.24,27.32) |  | 1.98(0.78,5.09) | 2.54(1.00,6.54) |  | -66.49(-84.48,-6.28) | -4.10(-4.54,-3.66) |
| Saudi Arabia | 676.49(373.10,1209.68) | 10.32(5.69,18.46) |  | 95.78(35.25,265.38) | 1.27(0.47,3.51) |  | -85.84(-95.94,-56.41) | -6.07(-6.63,-5.51) |
| Senegal | 550.44(233.89,1250.05) | 15.08(6.41,34.24) |  | 408.34(232.69,716.72) | 6.42(3.66,11.27) |  | -25.82(-59.08,53.16) | -2.31(-2.80,-1.81) |
| Serbia | 28.75(15.20,92.10) | 1.33(0.70,4.25) |  | 4.28(1.90,23.21) | 0.32(0.14,1.75) |  | -85.11(-94.34,-65.72) | -4.72(-5.15,-4.30) |
| Seychelles | 0.46(0.26,1.17) | 1.95(1.08,4.91) |  | 0.36(0.19,0.81) | 1.53(0.83,3.45) |  | -22.76(-52.42,33.31) | 0.15(-0.22,0.51) |
| Sierra Leone | 498.91(181.04,1222.92) | 27.52(9.99,67.47) |  | 511.08(292.41,954.80) | 14.29(8.18,26.70) |  | 2.44(-42.88,125.33) | -1.98(-2.19,-1.77) |
| Singapore | 149.85(133.16,167.32) | 23.08(20.51,25.77) |  | 37.20(30.50,44.82) | 4.58(3.76,5.52) |  | -75.17(-80.22,-69.65) | -4.34(-4.81,-3.88) |
| Slovakia | 16.52(9.58,58.38) | 1.25(0.72,4.40) |  | 4.29(2.34,17.39) | 0.50(0.27,2.03) |  | -74.01(-83.13,-58.57) | -2.30(-2.57,-2.02) |
| Slovenia | 0.88(0.68,1.09) | 0.21(0.17,0.26) |  | 0.45(0.34,0.57) | 0.14(0.11,0.18) |  | -49.12(-57.86,-38.64) | -1.50(-1.64,-1.35) |
| Solomon Islands | 11.08(5.69,19.24) | 7.12(3.66,12.36) |  | 10.80(5.67,21.44) | 4.15(2.18,8.24) |  | -2.52(-42.40,59.83) | -1.76(-1.93,-1.58) |
| Somalia | 885.86(317.22,1813.00) | 22.74(8.14,46.54) |  | 1167.39(417.63,2469.90) | 11.30(4.04,23.91) |  | 31.78(-35.30,148.12) | -1.96(-2.21,-1.71) |
| South Africa | 553.54(375.35,810.99) | 4.07(2.76,5.96) |  | 298.59(223.10,396.33) | 1.96(1.47,2.61) |  | -46.06(-64.33,-16.43) | -1.63(-1.95,-1.31) |
| South Sudan | 708.36(235.36,1713.76) | 26.99(8.97,65.31) |  | 730.49(356.08,1456.11) | 17.01(8.29,33.90) |  | 3.12(-36.15,125.38) | -1.38(-1.79,-0.97) |
| Spain | 562.60(510.29,620.74) | 7.18(6.51,7.92) |  | 206.78(176.38,234.13) | 3.19(2.72,3.61) |  | -63.25(-69.84,-56.75) | -2.56(-3.22,-1.90) |
| Sri Lanka | 861.09(604.31,1434.60) | 15.56(10.92,25.93) |  | 388.77(254.15,552.78) | 7.62(4.98,10.83) |  | -54.85(-80.37,-25.40) | -1.46(-2.09,-0.83) |
| Sudan | 4346.44(1806.96,9482.80) | 48.88(20.32,106.64) |  | 4800.53(2327.69,7183.65) | 28.94(14.03,43.30) |  | 10.45(-55.11,150.25) | -0.73(-1.34,-0.11) |
| Suriname | 83.62(36.06,114.63) | 64.19(27.68,87.99) |  | 23.30(14.88,35.10) | 16.27(10.39,24.50) |  | -72.13(-83.18,-32.14) | -4.95(-5.27,-4.62) |
| Sweden | 115.26(105.36,125.06) | 7.46(6.82,8.10) |  | 41.51(36.16,47.62) | 2.28(1.99,2.62) |  | -63.99(-69.01,-57.92) | -3.40(-3.89,-2.90) |
| Switzerland | 149.20(112.94,190.05) | 12.91(9.77,16.45) |  | 43.74(37.21,51.32) | 3.28(2.79,3.85) |  | -70.68(-78.43,-58.30) | -4.46(-4.65,-4.26) |
| Syrian Arab Republic | 588.00(270.46,1820.93) | 9.93(4.57,30.75) |  | 81.39(34.95,322.94) | 2.22(0.95,8.82) |  | -86.16(-94.81,-72.34) | -4.79(-5.36,-4.22) |
| Taiwan (Province of China) | 30.98(27.95,34.37) | 0.56(0.51,0.62) |  | 27.49(23.35,31.61) | 0.93(0.79,1.07) |  | -11.27(-26.96,5.66) | 4.90(3.52,6.28) |
| Tajikistan | 671.92(309.13,1088.06) | 28.94(13.31,46.86) |  | 555.66(359.63,798.29) | 15.50(10.03,22.27) |  | -17.30(-49.81,58.72) | -1.84(-2.10,-1.57) |
| Thailand | 791.75(452.41,1656.59) | 4.70(2.68,9.83) |  | 229.16(144.51,487.55) | 2.35(1.48,4.99) |  | -71.06(-84.45,-49.13) | -2.37(-2.73,-2.01) |
| Timor-Leste | 53.86(19.27,155.86) | 16.19(5.79,46.86) |  | 42.00(23.91,83.56) | 8.07(4.59,16.05) |  | -22.01(-58.61,80.99) | -2.46(-2.68,-2.23) |
| Togo | 195.70(77.17,445.47) | 11.10(4.38,25.27) |  | 196.30(122.00,307.53) | 5.93(3.69,9.29) |  | 0.31(-42.30,102.36) | -1.70(-2.02,-1.39) |
| Tokelau | 0.03(0.02,0.06) | 5.07(3.07,9.36) |  | 0.07(0.04,0.19) | 18.50(9.78,48.77) |  | 137.36(22.26,336.56) | -0.29(-2.03,1.49) |
| Tonga | 2.14(1.41,3.76) | 5.12(3.36,9.00) |  | 1.31(0.74,2.91) | 3.35(1.89,7.45) |  | -38.91(-64.49,-1.24) | -1.28(-1.49,-1.06) |
| Trinidad and Tobago | 109.63(91.55,129.04) | 26.98(22.53,31.76) |  | 14.85(11.05,19.36) | 5.45(4.06,7.11) |  | -86.45(-90.48,-81.27) | -5.51(-6.10,-4.92) |
| Tunisia | 616.37(350.81,1079.18) | 19.85(11.30,34.75) |  | 168.92(101.11,261.33) | 6.11(3.66,9.45) |  | -72.59(-89.13,-45.60) | -2.55(-3.04,-2.06) |
| Turkey | 23026.43(8402.12,41336.78) | 112.39(41.01,201.75) |  | 3298.87(2381.82,4447.68) | 17.81(12.86,24.01) |  | -85.67(-92.49,-63.12) | -5.25(-5.51,-4.99) |
| Turkmenistan | 38.26(20.68,56.75) | 2.55(1.38,3.78) |  | 17.02(13.68,20.74) | 1.12(0.90,1.36) |  | -55.51(-69.33,-23.19) | -3.27(-3.98,-2.55) |
| Tuvalu | 0.52(0.28,0.99) | 15.04(8.02,28.53) |  | 0.18(0.11,0.35) | 4.85(2.96,9.46) |  | -65.40(-81.73,-29.90) | -3.43(-3.63,-3.23) |
| Uganda | 1857.35(695.28,3806.24) | 22.06(8.26,45.21) |  | 1429.98(801.44,2798.23) | 7.21(4.04,14.11) |  | -23.01(-53.27,45.07) | -3.64(-3.84,-3.43) |
| Ukraine | 193.20(140.29,258.93) | 1.70(1.23,2.28) |  | 85.79(70.52,102.21) | 1.35(1.11,1.61) |  | -55.60(-68.48,-38.13) | -0.13(-0.50,0.24) |
| United Arab Emirates | 247.43(148.53,389.08) | 41.98(25.20,66.01) |  | 82.39(52.17,146.37) | 6.15(3.90,10.93) |  | -66.70(-81.46,-36.71) | -4.18(-4.80,-3.56) |
| United Kingdom | 1208.06(941.90,1520.54) | 11.06(8.63,13.92) |  | 364.41(314.02,406.72) | 3.09(2.67,3.45) |  | -69.84(-76.32,-61.11) | -3.32(-3.65,-2.99) |
| United Republic of Tanzania | 1877.63(890.82,4522.98) | 15.55(7.38,37.46) |  | 1842.33(1035.81,3590.38) | 7.55(4.24,14.71) |  | -1.88(-40.03,79.51) | -1.95(-2.14,-1.76) |
| United States of America | 5918.54(5306.81,6711.73) | 10.59(9.49,12.00) |  | 2873.97(2586.58,3188.08) | 4.84(4.35,5.36) |  | -51.44(-59.13,-43.42) | -2.53(-2.73,-2.33) |
| United States Virgin Islands | 6.07(3.42,8.45) | 18.99(10.70,26.44) |  | 0.32(0.19,0.53) | 2.41(1.44,3.97) |  | -94.69(-97.12,-83.59) | -6.59(-6.97,-6.22) |
| Uruguay | 102.12(89.11,117.37) | 12.48(10.89,14.34) |  | 16.15(13.01,19.64) | 2.45(1.97,2.98) |  | -84.19(-87.72,-79.27) | -5.36(-5.63,-5.09) |
| Uzbekistan | 619.83(503.42,776.75) | 7.24(5.88,9.08) |  | 340.00(280.15,416.21) | 3.37(2.78,4.12) |  | -45.15(-58.98,-24.79) | -1.01(-1.75,-0.26) |
| Vanuatu | 6.24(3.48,11.51) | 9.17(5.11,16.91) |  | 6.86(3.67,14.71) | 5.89(3.15,12.63) |  | 9.95(-30.78,80.22) | -1.36(-1.74,-0.99) |
| Venezuela (Bolivarian Republic of) | 425.35(360.63,503.01) | 6.00(5.08,7.09) |  | 121.66(85.17,169.66) | 1.84(1.29,2.56) |  | -71.40(-79.83,-60.68) | -4.21(-4.84,-3.58) |
| Viet Nam | 1262.23(786.23,2340.26) | 4.76(2.97,8.83) |  | 533.43(282.70,1095.61) | 2.15(1.14,4.42) |  | -57.74(-77.57,-22.32) | -1.68(-2.03,-1.32) |
| Yemen | 2699.72(1280.20,4942.57) | 38.05(18.05,69.67) |  | 3665.62(2055.18,5446.07) | 26.58(14.90,39.50) |  | 35.78(-36.11,149.70) | -0.35(-0.89,0.20) |
| Zambia | 476.43(207.98,1282.84) | 12.69(5.54,34.17) |  | 369.05(225.11,683.56) | 4.46(2.72,8.26) |  | -22.54(-59.32,64.64) | -3.06(-3.37,-2.74) |
| Zimbabwe | 127.56(75.91,201.41) | 2.65(1.58,4.18) |  | 257.52(163.88,406.46) | 4.09(2.60,6.46) |  | 101.88(23.49,221.28) | 2.68(2.11,3.25) |
